# Neutral Rhenadicarbaboranes with Re(CO)_2_(NO) Vertices: A Theoretical Study of Building Blocks for Rhenacarborane-Based Drug Delivery Agents

**DOI:** 10.3390/molecules25010110

**Published:** 2019-12-27

**Authors:** Amr A. A. Attia, Alexandru Lupan, Radu Silaghi-Dumitrescu, R. Bruce King

**Affiliations:** 1 Faculty of Chemistry and Chemical Engineering, Babeș-Bolyai University, Cluj-Napoca 400084, Romania; amrattia@chem.ubbcluj.ro (A.A.A.A.); rsilaghi@chem.ubbcluj.ro (R.S.-D.); 2 Department of Chemistry, University of Georgia, Athens, GA 30602, USA

**Keywords:** rhenium’ dicarbaboranes, metal carbonyls, metal nitrosyls, density functional theory

## Abstract

The rhenadicarbaborane carbonyl nitrosyls (C_2_B*_n_*_−3_H*_n_*_−1_)Re(CO)_2_(NO), (*n* = 8 to 12), of interest in drug delivery agents based on the experimentally known C_2_B_9_H_11_Re(CO)_2_(NO) and related species, have been investigated by density functional theory. The lowest energy structures of these rhenadicarbaboranes are all found to have central ReC_2_B*_n_*_−3_ most spherical *closo* deltahedra in accord with their 2*n* + 2 Wadean skeletal electrons. Carbon atoms are found to be located preferentially at degree 4 vertices in such structures. Furthermore, rhenium atoms are preferentially located at a highest degree vertex, typically a vertex of degree 5. Only for the 9-vertex C_2_B_6_H_8_Re(CO)_2_(NO) system are alternative *isocloso* deltahedral isomers found within ~8 kcal/mol of the lowest energy *closo* isomer. Such 9-vertex *isocloso* structures provide a degree 6 vertex for the rhenium atom flanked by degree 4 vertices for each carbon atom.

## 1. Introduction

Carboranes in general are accepted as synthons not unlike organic ones insofar as biological and medical applications are concerned [1]. The very common icosahedral C_2_B_10_ synthon is thus regarded as similar to rotating phenyl groups. This similarity is seen in terms of steric requirements, polarity/hydrophobicity, and availability of regioselective functionalization with substituents at the carbon atoms [1]. Such regioselective functionalization has been shown to work with all classes of bioactive substances, including aminoacids, lipids, and nucleosides, as well as incorporation into dendrimers, liposomes, and other biocompatible methods of nanoencapsulation [1]. In this connection carboranes also provide two specific advantages: the presence of boron, which allows for radiotherapeutical techniques such as boron neutron capture therapy (BNCT), and the availability of viable syntheses leading to endo-substitution with transition metal synthons to yield metallacarboranes [1,2,3,4,5,6,7,8,9].

With such potential, carboranes have been shown to act as agonists or, depending on the substitution, antagonists, of the estrogen receptor protein [4]. Thus the steric and hydrophobic similarity/compatibility of the carborane unit with the non-aromatic part of the estrogen allows it to compete efficiently with the latter, either as a substitute or as an inhibitor [1]. Carboranes as hydrophobic units can be incorporated into a host of other proteins. These include the androgen receptors (e.g., with testosterone as target, leading to carborane-containing derivatives shown to outperform currently used drugs against prostate cancer) [10], retinoic acid receptors (with successful tests performed on human promyelelocytic leukemia HL-60 cells—thus again with anti-cancer potential) [11]. Other such proteins include transthyretin (a target of non-steroidal anti-inflammatory drugs), NSAIDS, where carborane derivatives have the unique advantage of not showing concomitant affinity for cyclooxygenase enzymes, COX, and hence displaying reduced side-effects as anti-inflammatory agents compared to typical NSAIDS. Interestingly, the lack of inhibition in cyclooxygenase also arises from the analogy of carboranyl with phenyl. However, in this case COX inhibition would require a *non*-rotating phenyl ring) [1]. Other such proteins include HIV protease (in this case with ionic carborane derivatives but still with a key role for hydrophobic interactions) [12,13], α-thrombin (acting as anticoagulants) [11], as well as a range of derivatives aimed at accumulating boron in tumor cells for BNCT (e.g., with nucleosides). A more recent application involves BNCS (boron neutron capture synovectomy, specifically for removal of synovial tissue) [14,15], or coupled with cytotoxic metals such as platinum or tin, or coupled with light-sensitive moieties for use in photodynamic therapy (PDT) [1]. The range of metals of the metallacarboranes exhibiting biological reactivity as described above includes Fe, Co, V, Ta, Mo, Nb, Tc, Re [1]. A particular set of applications is the one regarding radioimaging and radiotherapy. For imaging, in addition to halogen radioactive isotopes, technetium is the most widely used [1]. Rhenium (and hence rhenacarboranes) has been proposed as a convenient substitute for technetium for preliminary laboratory and in vitro studies, since the two metals are reasonably similar in properties insofar their metallacarboranes are concerned. In addition, rhenium is much more readily available and non-radioactive [1,10,11].

Rhenium has also been shown to be useful independently of technetium, such as in the iodine-radioactive 3-NO-3,3-κ^2^-(2,2′-N_2_C_10_H_6_(Me){(CH_2_)_7_^131^I}-4,4′)-*closo*-3,1,2-ReC_2_B_9_H_11_ rhenacarborane and in subsequently reported members of its family, [3,3-(CO)_2_-3-NO-*closo*-Re(8-O(CH_2_)_2_O(CH_2_)_2_NH_3_-3,1,2-C_2_B_9_H_10_)]BF_4_ and [3,3-(CO)_2_-3-NO-*closo*-Re(8-O(CH_2_)_2_O(CH_2_)_2_OH-3,1,2-C_2_B_9_H_10_)] with a range of other compounds also available synthetically [1]. Such molecules have the remarkable property of passing the blood-brain barrier with the hydrophobicity of the carborane and the charge-neutral character of the nitrosyl/carbonyl-metal moiety being essential towards achieving this goal. Such observations suggest clear potential for therapeutic agents directed within the central nervous system either by delivering radioactive material via the substituents of the carborane system, or by delivering other conjugated therapeutic agents which would not otherwise pass the blood-brain barrier, such as peptides [1,7]. Also, when rhenacarboranes are compared to their technetium analogs, they complement their lack of radioactivity with a useful degree of luminescence with a potential for in vitro and in vivo imaging. The latter, correlated with the distinct affinity for biological targets such as the estrogen receptor, has fueled further synthetic interest into rhenacarboranes and into their biologically-relevant reactivity, structure and stability [1,2,3,4,5,6,7,8,9].

The chemistry of such rhenacarboranes dates back to the 1965 synthesis of the icosahedral dicarbaborane rhenium carbonyl monoanion [3,1,2-C_2_B_9_H_11_Re(CO)_3_]^−^ as its cesium salt [16,17]. Replacement of one of the carbonyl groups in [3,1,2-C_2_B_9_H_11_Re(CO)_3_]^−^ with NO^+^ gives the neutral 3,1,2-C_2_B_9_H_11_Re(CO)_2_(NO) originally synthesized by Stone and co-workers [18] but then later used by Jelliss and coworkers as a building block for their work on rhenacarborane-based drug delivery agents [1,7]. Related neutral rhenacarborane derivatives include the tricarbaborane derivatives (C_3_B*_n_*_−4_H*_n_*_−1_)Re(CO)_3_, which are direct analogues of the stable (η^5^-C_5_H_5_)Re(CO)_3_. However, suitable precursors for rhenatricarbaborane derivatives are less accessible synthetically than those for rhenadicarbaboranes since dicarbaborane precursors are readily accessible from boranes and alkynes. The chemical robustness of certain rhenacarboranes has additionally made them of interest as possible vehicles for drug delivery in therapeutic and diagnostic applications [19]. Thus suitably designed kinetically inert rhenacarborane polyhedra can survive reaction conditions required to introduce external functionalities for optimal introduction into biological systems. In addition, such rhenacarboranes can survive metabolic degradation.

**Figure 1:**
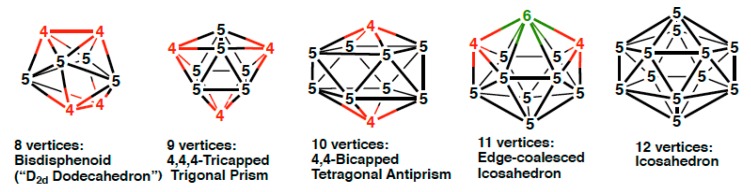
The most spherical (*closo*) deltahedra having from 8 to 12 vertices indicating their vertex degrees. Vertices of degree 4, 5, and 6 are also indicated in red, black, and green, respectively, in Figure 1 and Figure 2.

The central polyhedra in the three types of rhenacarboranes, namely [(C_2_B*_n_*_−3_H*_n_*_−1_)Re(CO)_3_]^−^, (C_2_B*_n_*_−3_H*_n_*_−1_)Re(CO)_2_(NO), and (C_3_B*_n_*_−4_H*_n_*_−1_)Re(CO)_3_, all have 2*n* + 2 skeletal electrons, where BH, CH, Re(CO)_3_, and Re(CO)_2_(NO) vertices contribute 2, 3, 1, and 2 skeletal electrons, respectively, assuming contributions of three internal orbitals from each vertex atom towards the skeletal bonding. Such rhenacarboranes therefore might be expected to exhibit most spherical *closo* deltahedral structures (Figure 1) in accord with the Wade-Mingos rules [20,21,22]. The *closo* deltahedra for the 8- through 12-vertex systems have only degree 4 and 5 vertices except for the 11-vertex system, which necessarily has one degree 6 vertex. However, density functional theory studies [23] show that the asymmetry in the C_3_B*_n_*_−4_Re polyhedra leads to deviation from the most spherical deltahedra to give lowest energy structures for the 8- and 10-vertex systems with two degree 6 vertices. We now report similar density theory functional studies on the neutral (C_2_B*_n_*_−3_H*_n_*_−1_)Re(CO)_2_(NO) systems (*n* = 9 to 12), which are the basis for the rhenacarborane drug delivery systems.

**Figure 2:**
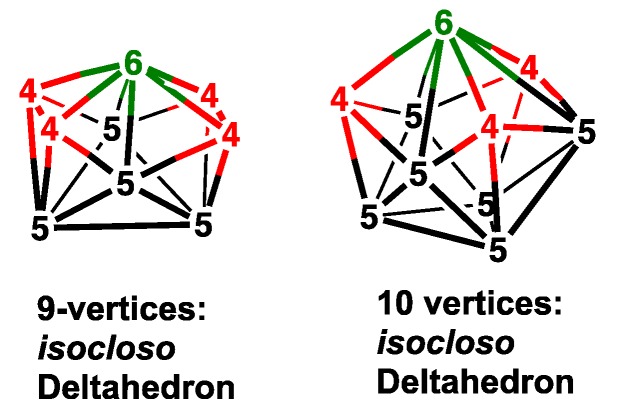
The *isocloso* deltahedra for the 9- and 10-vertex systems.

Alternative deltahedra to be considered for the 9- and 10-vertex rhenadicarboranes are the *isocloso* deltahedra, which, unlike the corresponding *closo* deltahedra, provide a degree 6 vertex for the rhenium atom (Figure 2) [24,25,26]. Conversion of a *closo* deltahedron to an *isocloso* deltahedron with the same number of vertices involves a diamond-square-diamond rearrangement converting a pair of degree 5 vertices to a degree 6 and a degree 4 vertex. Normally *isocloso* deltahedral metallaborane structures are found in systems with 2*n* skeletal electrons rather than 2*n* + 2 skeletal electrons. However, the *closo*→*isocloso* metallaborane conversion for the 9- and 10-vertex rhenacarboranes can provide a degree 6 vertex for the rhenium atom and an additional more favorable degree 4 rather than a degree 5 vertex for a carbon atom. For the 10-vertex rhenatricarbaborane C_3_B_6_H_9_Re(CO)_3_ the lowest energy structures are found to be *isocloso* rather than *closo* deltahedra (compare Figure 1 and Figure 2) thereby providing a favorable degree 4 vertex for each of the three carbon atoms in addition to a degree 6 vertex for the rhenium atom.

## 2. Results and Discussion

### 2.1. Eight-Vertex C_2_B_5_H_7_Re(CO)_2_(NO) Structures

**Figure 3:**
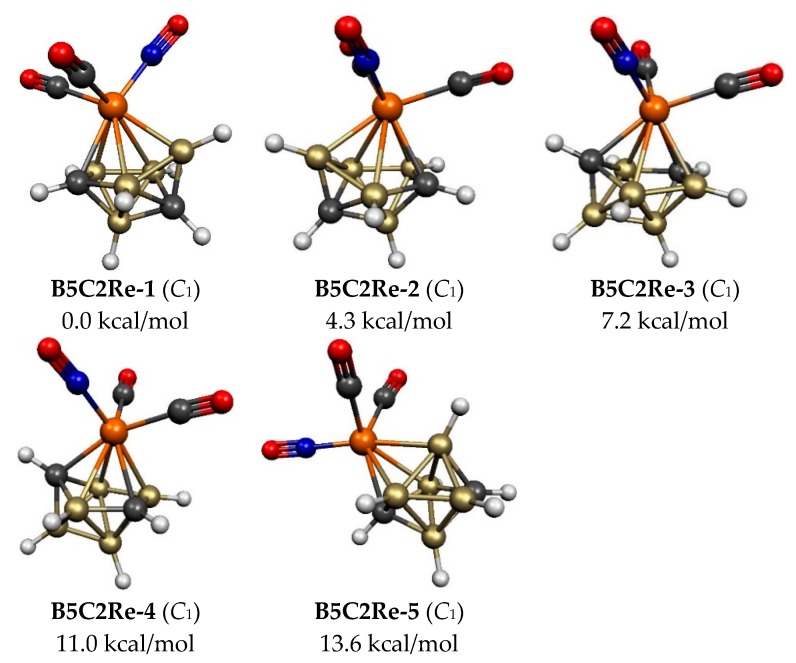
The five optimized lowest energy 8-vertex C_2_B_5_H_7_Re(CO)_2_(NO) structures.

Five 8-vertex C_2_B_5_H_7_Re(CO)_2_(NO) structures were found within 26 kcal/mol of the lowest energy structure **B5C2Re-1** (Figure 3 and Table 1). All of these five structures were found to have the expected central ReC_2_B_5_ bisdisphenoid, which is the most spherical *closo* deltahedron consistent with the 2*n* + 2 Wadean skeletal electrons in this system. The four lowest-energy structures have the rhenium atom located at a degree 5 vertex and both carbon atoms located at non-adjacent degree 4 vertices separated by a single boron atom. This corresponds to C…C distances in the range 2.6 to 2.7 Å. The lowest energy structure **B5C2Re-1** as well as **B5C2Re-2**, lying 4.3 kcal/mol in energy above **B5C2Re-1**, have one Re–C edge and differ by rotation of the Re(CO)_2_(NO) moiety. The next higher energy C_2_B_5_H_7_Re(CO)_2_(NO) structures, namely **B5C2Re-3** and **B5C2Re-4**, lying 7.2 and 11.0 kcal/mol, respectively, in energy above **B5C2Re-1**, have two Re–C edges and again differ mainly by rotation of the Re(CO)_2_(NO) moiety. Structure **B5C2Re-5**, lying 13.6 kcal/mol in energy above **B5C2Re-1**, has the rhenium atom located at a degree 4 vertex and one Re–C edge.

### 2.2. Nine-Vertex C_2_B_6_H_8_Re(CO)_2_(NO) Structures

**Figure 4:**
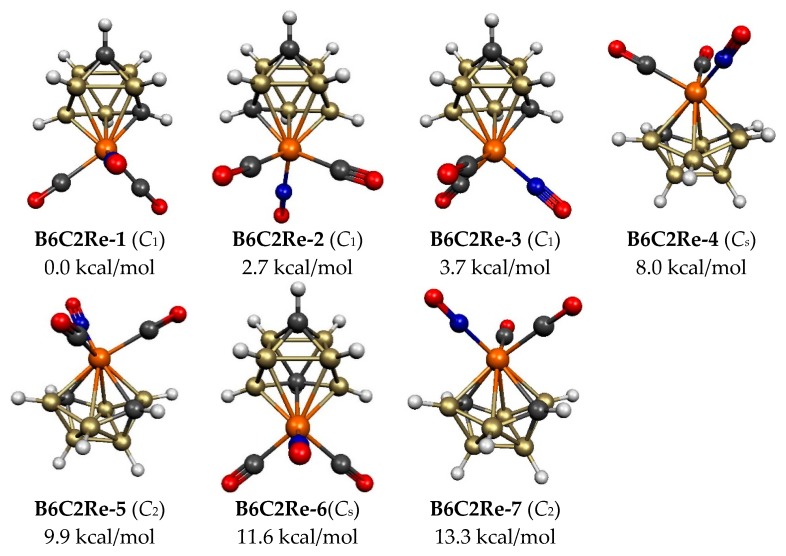
The seven optimized lowest energy 9-vertex C_2_B_6_H_8_Re(CO)_2_(NO) structures.

The lowest energy C_2_B_6_H_8_Re(CO)_2_(NO) structure **B6C2Re-1**, as well as the next two higher energy structures **B6C2Re-2** and **B6C2Re-3**, lying 2.7 and 3.7 kcal/mol, respectively, in energy above **B6C2Re-1**, all have a central ReC_2_B_6_ tricapped trigonal prism, which is the most spherical *closo* 9-vertex deltahedron in accord with the Wade–Mingos rules for this 2*n* + 2 skeletal electron system (Figure 4 and Table 2). All three of these structures have the only possible arrangement of a degree 5 rhenium vertex, and two degree 4 carbon vertices. They differ only in the rotation of the Re(CO)_2_(NO) unit relative to the ReC_2_B_6_ cage. The higher energy C_2_B_6_H_8_Re(CO)_2_(NO) structure **B6C2Re-6**, lying 11.6 kcal/mol in energy above C_2_B_6_H_8_Re(CO)_2_(NO) structure **B6C2Re-1**, also has a central ReC_2_B_6_ tricapped trigonal prism but with one of the carbon atoms located at a less favorable degree 5 vertex.

In addition to these four C_2_B_6_H_8_Re(CO)_2_(NO) structures with a central ReC_2_B_6_ tricapped trigonal prism, three C_2_B_6_H_8_Re(CO)_2_(NO) structures, namely **B6C2Re-4**, **B6C2Re-5**, and **B6C2Re-7**, lying 8.0, 9.9 and 13.3 kcal/mol in energy above **B6C2Re-1**, are found with a central ReC_2_B_6_ *isocloso* deltahedron, thereby providing a degree 6 vertex for the rhenium atom (Figure 2 and Figure 4 and Table 2). All three isocloso C_2_B_6_H_8_Re(CO)_2_(NO) structures have both carbon atoms located at degree 4 vertices necessarily adjacent to the rhenium vertex thus leading to two Re–C edges. Structure **B6C2Re-4** with *C_s_* symmetry has the two carbon vertices located on a side of the rectangle of the four degree 4 vertices. However, structures **B6C2Re-5** and **B6C2Re-7**, each with C_2_ symmetry, have their two carbon vertices located on a diagonal of the rectangle of the four degree 4 vertices. Structures **B6C2Re-5** and **B6C2Re-7** differ in the rotation of the Re(CO)_2_(NO) unit relative to the ReC_2_B_6_ isocloso deltahedron.

### 2.3. Ten-Vertex C_2_B_7_H_9_Re(CO)_2_(NO) Structures

**Figure 5:**
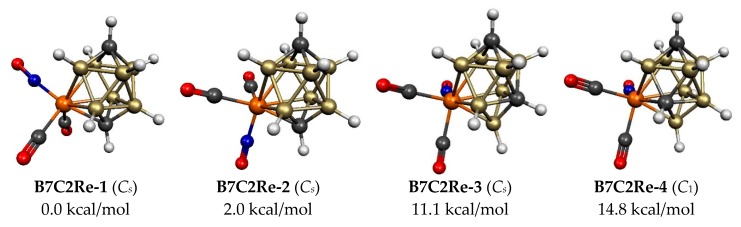
The four optimized lowest energy 10-vertex C_2_B_7_H_9_Re(CO)_2_(NO) structures.

Four 10-vertex C_2_B_7_H_9_Re(CO)_2_(NO) structures were found within 16 kcal/mol of the lowest energy structure **B7C2Re-1** (Figure 5 and Table 3). All of these structures have a central ReC_2_B_7_ bicapped tetragonal antiprism, which is the 10-vertex *closo* deltahedron consistent with the 2*n* + 2 = 22 skeletal electrons for this system. The lowest energy C_2_B_7_H_9_Re(CO)_2_(NO) structure **B7C2Re-1** as well as the slightly higher energy structure **B7C2Re-2**, lying only 2.0 kcal/mol in energy above **B7C2Re-1**, have the only possible arrangement with the two carbon atoms located at the two degree 4 vertices necessarily leading to a single Re–C edge. The antipodal positions of the two carbon vertices in **B7C2Re-1** and **B7C2Re-2** lead to relative long C…C distances of 3.42 Å. Structures **B7C2Re-1** and **B7C2Re-2** differ only in the orientation of the Re(CO)_2_(NO) unit. The two next higher energy C_2_B_7_H_9_Re(CO)_2_(NO) structures, namely **B7C2Re-3** and **B7C2Re-4**, lying 11.1 and 14.8 kcal/mol in energy, respectively, above **B7C2Re-1**, have the energetically less desirable feature of one of the carbon atoms located at a degree 5 rather than a degree 4 vertex. In **B7C2Re-3**, neither carbon vertex is adjacent to the rhenium vertex so there are no Re–C edges in the ReC_2_B_7_ deltahedron. However, in **B7C2Re-4**, the degree 5 carbon vertex is adjacent to the rhenium vertex so that there is an Re–C edge.

The clear energetic preference of the 10-vertex rhenadicarbaborane C_2_B_7_H_9_Re(CO)_2_(NO) for the *closo* bicapped tetragonal antiprism structures (Figure 1) contrasts with the previously discovered [23] energetic preference of the 10-vertex rhenatricarbaborane C_3_B_6_H_9_Re(CO)_3_ for *isocloso* deltahedral structures (Figure 2). This difference may relate to the number of carbon vertices in the central deltahedron. For the rhenadicarbaboranes C_2_B_7_H_9_Re(CO)_2_(NO), the *closo* structure provides the two degree 4 vertices required for the two carbon atoms. However, for the rhenatricarbaboranes C_3_B_6_H_9_Re(CO)_3_, an *isocloso* structure is required to provide the three degree 4 vertices to accommodate all three carbon atoms.

### 2.4. Eleven-Vertex C_2_B_8_H_10_Re(CO)_2_(NO) Structures

**Figure 6:**
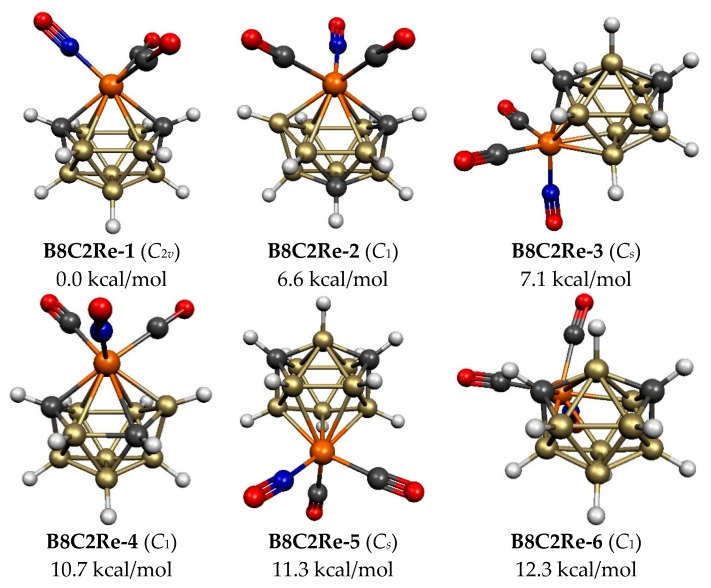
The six optimized lowest energy 11-vertex C_2_B_8_H_10_Re(CO)_2_(NO) structures.

The 11-vertex *closo* deltahedron (Figure 1) has a single degree 6 vertex and thus also can function as an *isocloso* metallaborane with a metal atom at the degree 6 vertex. The six lowest energy C_2_B_8_H_10_Re(CO)_2_(NO) structures are all based on this deltahedron (Figure 6 and Table 4). The lowest energy such structure **B8C2Re-1** has the ideal arrangement with the rhenium atom located at the lone degree 6 vertex and the carbon atoms located at the two degree 4 vertices, thereby leading to two Re-C edges. The next highest energy C_2_B_8_H_10_Re(CO)_2_(NO) structure **B8C2Re-2**, lying 6.6 kcal/mol in energy above **B8C2Re-1**, has the rhenium atom still located at the degree 6 vertex but one of the carbon atoms has moved from a degree 4 vertex to a degree 5 vertex not adjacent to the other carbon atom. Structure **B8C2Re-4**, lying 10.7 kcal/mol in energy above **B8C2Re-1**, also has the rhenium atom located at the degree 6 vertex, one carbon atom located at a degree 4 vertex, and the other carbon atom located at a degree 5 vertex different from the degree 5 carbon vertex in **B8C2Re-2**.

The three remaining C_2_B_8_H_10_Re(CO)_2_(NO) structures, namely **B8C2Re-3**, **B8C2Re-5**, and **B8C2Re-6**, lying 7.1, 11.3, and 12.3 kcal/mol, respectively, in energy above **B8C2Re-1**, each have the two carbon atoms located at their two degree 4 vertices and the rhenium atom located at a degree 5 vertex (Figure 6 and Table 4). These three structures differ in the location of the degree 5 rhenium vertex relative to the degree 6 and degree 4 vertices of the underlying 11-vertex *closo* deltahedron. Thus, in **B8C2Re-5**, the rhenium atom is located at the vertex furthest from the degree 6 vertex leading to an Re–B (deg 6) distance of 3.54 Å. In **B8C2Re-6**, the rhenium atom is located at a degree 5 vertex adjacent to the degree 6 vertex with a Re–B (deg 6) edge of length 2.65 Å. However, in **B8C2Re-3**, the rhenium atom is located at a degree 5 vertex adjacent to a degree 4 vertex but not adjacent to the degree 6 vertex leading to an an Re–B (deg 6) distance of 3.40 Å.

### 2.5. Twelve-Vertex C_2_B_9_H_11_Re(CO)_2_(NO) Structures

**Figure 7:**
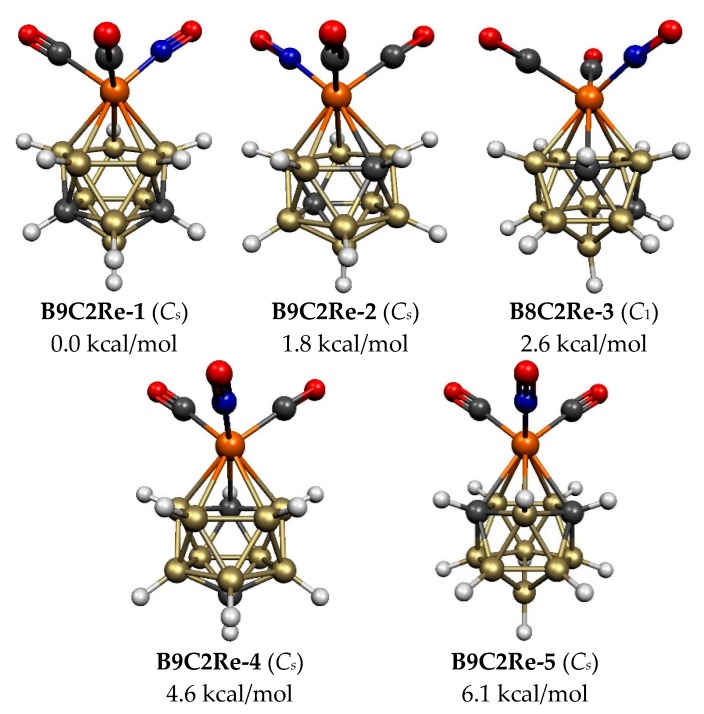
The five optimized lowest energy 12-vertex C_2_B_9_H_11_Re(CO)_2_(NO) structures.

The five lowest energy 12-vertex C_2_B_9_H_11_Re(CO)_2_(NO) structures are all based on the regular icosahedron with all degree 5 vertices consistent with their 2*n* + 2 skeletal electrons for this *closo* deltahedron (Figure 1 and Figure 7, Table 5). None of these five icosahedral C_2_B_9_H_11_Re(CO)_2_(NO) have a C–C edge consistent with the pattern of the smaller (C_2_B*_n_*_−3_H*_n_*_−1_)Re(CO)_2_(NO) structures discussed above. The lowest energy C_2_B_9_H_11_Re(CO)_2_(NO) structure **B9C2Re-1** is the unique structure not only without a C-C edge but also without any Re–C edges. The nest lowest energy C_2_B_9_H_11_Re(CO)_2_(NO) structure **B9C2Re-2**, lying only 1.8 kcal/mol in energy above **B9C2Re-1**, is the unique icosahedral structure with the carbon atoms in antipodal positions leading to a C…C distance of 3.05 Å. The next three C_2_B_9_H_11_Re(CO)_2_(NO) structures, namely **B9C2Re-3**, **B9C2Re-4**, and **B9C2Re-5**, lying 2.6, 4.6, and 6.1 kcal/mol in energy above **B9C2Re-1**, have the pair of carbon atoms located in non-adjacent non-antipodal positions separated by a single boron vertex in one direction leading to C–C distances of ~2.6 Å. Structures **B9C2Re-3** and **B9C2Re-4** have one Re–C edge whereas, **B9C2Re-5** is the unique possible icosahedral structure with two Re–C edges.

## 3. Theoretical Methods

The rhenadicarbaborane structures investigated in this study are derived from the borane dianions B*_n_*H*_n_*^2−^ by substituting a BH vertex with an Re(CO)_2_(NO) unit followed by the replacement of two additional boron atoms with two carbon atoms. A total of 48 polyhedral frameworks ranging from 8 to 12 vertices were generated in this way leading to 2426 initial structures of the type C_2_B*_n_*_−3_H*_n_*_−1_Re(CO)_2_(NO) (*n* = 8 to 12) (see Supporting Information).

Geometry optimizations of these initial structures were performed using the B3LYP DFT functional coupled with the SDD (Stuttgart Dresden ECP plus DZ) basis set for rhenium and the double zeta 6-31G(d) basis set for the lighter atoms as implemented in the Gaussian09 suite of programs [27]. All optimized structures were characterized by harmonic vibrational frequencies. Saddle point structures with imaginary vibrational frequencies were reoptimized by following the normal modes in order to obtain genuine minima. The energetically most stable isomers were further optimized by employing the M06L DFT functional and the 6-311G(d,p)//SDD basis sets. All of the resulting structures were found to have substantial HOMO-LUMO gaps ranging from 2.2 to 3.0 eV (see the Supporting Information).

The shorthand notation **B(n-3)C2Re-x** was assigned to all structures discussed in this work where **n** is the total number of polyhedral vertices, and **x** is the energy ranking of the structure on the potential energy surface. Additional information on higher energy structures and connectivities can be viewed in the Supporting Information.

## 4. Summary

The lowest energy (C_2_B*_n_*_−3_H*_n_*_−1_)Re(CO)_2_(NO) structures are all found to have central ReC_2_B*_n_*_−3_ most spherical *closo* deltahedra in accord with their 2*n* + 2 Wadean skeletal electrons. Carbon atoms are preferentially located at degree 4 vertices whereas rhenium atoms are preferentially located at a highest degree vertex, typically a vertex of degree 5. Only for the 9-vertex C_2_B_6_H_8_Re(CO)_2_(NO) system are alternative *isocloso* deltahedral isomers found within 8 kcal/mol of the lowest energy *closo* isomer. Such 9-vertex *isocloso* structures provide a degree 6 vertex for the rhenium atom with adjacent degree 4 vertices for both carbon atoms.

## Figures and Tables

**Table 1:** The five optimized 8-vertex C_2_B_5_H_7_Re(CO)_2_(NO) structures within 26 kcal/mol of the lowest energy structure.

		Vertex Degrees		Re–C	C–C	
Structure	∆*E*	Re	C	Edges	Distance, Å	Comments
**B5C2Re-1**	0.0	5	4,4	1	2.60	bisdisphenoid
**B5C2Re-2**	4.3	5	4,4	1	2.59	bisdisphenoid
**B5C2Re-3**	7.2	5	4,4	2	2.70	bisdisphenoid
**B5C2Re-4**	11.0	5	4,4	2	2.70	bisdisphenoid
**B5C2Re-5**	13.6	4	4,4	1	2.59	bisdisphenoid

**Table 2:** The seven optimized 9-vertex C_2_B_6_H_8_Re(CO)_2_(NO) structures within 17 kcal/mol of the lowest energy structure.

Structure		Vertex Degrees		Re–C	C–C	
(Symmetry)	∆*E*	Re	C	Edges	Distance, Å	Comments
**B6C2Re-1 (*C*_1_)**	0.0	5	4.4	1	2.56	tricap trig prism
**B6C2Re-2 (*C*_1_)**	2.7	5	4,4	1	2.57	tricap trig prism
**B6C2Re-3 (*C*_1_)**	3.7	5	4,4	1	2.55	tricap trig prism
**B6C2Re-4 (*C_s_*)**	8.0	6	4,4	2	2.77(m)	9-vertex *isocloso*
**B6C2Re-5 (*C*_2_)**	9.9	6	4,4	2	3.17(p)	9-vertex *isocloso*
**B6C2Re-6 (*C_s_*)**	11.6	5	5,4	1	2.61	tricap trig prism
**B6C2Re-7 (*C*_2_)**	13.3	6	4,4	2	3.18(p)	9-vertex *isocloso*

**Table 3:** The four optimized 10-vertex C_2_B_7_H_9_Re(CO)_2_(NO) structures within 16 kcal/mol of the lowest energy structure.

Structure		Vertex Degrees		Re–C	C–C	
(Symmetry)	∆*E*	Re	C	Edges	Distance, Å	Comments
**B7C2Re-1 (*C*_s_)**	0.0	5	4,4	1	3.42	Bicap tetrag antipr
**B7C2Re-2 (*C_s_*)**	2.0	5	4,4	1	3.42	Bicap tetrag antipr
**B7C2Re-3 (*C_s_*)**	11.1	5	5,4	0	2.60	Bicap tetrag antipr
**B7C2Re-4 (*C*_1_)**	14.8	5	5,4	1	2.64	Bicap tetrag antipr

**Table 4:** The six optimized 11-vertex C_2_B_8_H_10_Re(CO)_2_(NO) structures within 15 kcal/mol of the lowest energy structure.

Structure		Vertex Degrees		Re–C	C–C	
(Symmetry)	∆*E*	Re	C	Edges	Distance, Å	Comments
**B8C2Re-1 (*C*_2*v*_)**	0.0	6	4.4	2	3.36	11-vertex *closo*
**B8C2Re-2 (*C*_1_)**	6.6	6	5.4	1	2.60	11-vertex *closo*
**B8C2Re-3 (*C_s_*)**	7.1	5	4,4	1	3.04	11-vertex *closo*
**B8C2Re-4 (*C*_1_)**	10.7	6	5,4	1	2.85	11-vertex *closo*
**B8C2Re-5 (*C_s_*)**	11.3	5	4,4	0	3.01	11-vertex *closo*
**B8C2Re-6 (*C*_1_)**	12.3	5	4,4	1	3.02	11-vertex *closo*

**Table 5:** The five optimized 12-vertex C_2_B_9_H_11_Re(CO)_2_(NO) structures within 15 kcal/mol of the lowest energy structure. All of these structures have central C_2_B_9_Re icosahedra with exclusively degree 5 vertices.

		Re–C	C–C
Structure (Symmetry)	∆*E*	Edges	Distance, Å
**B9C2Re-1 (*C*_1_)**	0.0	0	2.58
**B9C2Re-2 (*C*_1_)**	1.8	1	3.05
**B9C2Re-3 (*C*_1_)**	2.6	1	2.60
**B9C2Re-4 (*C*_1_)**	4.6	1	2.60
**B9C2Re-5 (*C*_1_)**	6.1	2	2.67

## Supplementary Materials

Table S1A–C. Initial models, distance matrices and energy rankings for the C_2_B_5_H_7_Re(CO)_2_NO structures. Table S2A–C. Initial models, distance matrices and energy rankings for the C_2_B_6_H_8_Re(CO)_2_NO structures. Table S3A–C. Initial models, distance matrices and energy rankings for the C_2_B_7_H_9_Re(CO)_2_NO structures. Table S4A–C. Initial models, distance matrices and energy rankings for the C_2_B_8_H_10_Re(CO)_2_NO structures. Table S5A–C. Initial models, distance matrices and energy rankings for the C_2_B_9_H_11_Re(CO)_2_NO structures. Table S6. Orbital energies and HOMO-LUMO gaps. Complete Gaussian09 reference.
