# Supplementary material for: Neutral Rhenadicarbaboranes with Re(CO)2(NO) Vertices: A Theoretical Study of Building Blocks for Rhenacarborane-Based Drug Delivery Agents
Source: Molecules. 2019 Dec 27;25(1):110. doi: 10.3390/molecules25010110 (PMC6983249; doi:10.3390/molecules25010110)
Supplement: Supplementary file 1 [file molecules-25-00110-s001.pdf]

Neutral Rhenadicarbaboranes with $Re(CO)_2(NO)$ Vertices: A Theoretical Study of Building Blocks for Rhenacarborane-based Drug Delivery Agents

Amr A. A. Attia,[1] Alexandru Lupan,*[1] Radu Silaghi-Dumitrescu,[1] and R. Bruce King*[2]

[1]*Faculty of Chemistry and Chemical Engineering, Babes-Bolyai University, ClujNapoca, Romania*

[2]*Department of Chemistry, University of Georgia, Athens, Georgia, 30602*

Supporting Information

Complete Gaussian09 Reference (reference 18).

Tables S1A-S1C. Initial models, distance matrices and energy rankings for the $C_2B_5H_7Re(CO)_2NO$ structures

Tables S2A-S2C. Initial models, distance matrices and energy rankings for the $C_2B_6H_8Re(CO)_2NO$ structures

Tables S3A-S3C. Initial models, distance matrices and energy rankings for the $C_2B_7H_9Re(CO)_2NO$ structures

Tables S4A-S4C. Initial models, distance matrices and energy rankings for the $C_2B_8H_{10}Re(CO)_2NO$ structures

Tables S5A-S5C. Initial models, distance matrices and energy rankings for the $C_2B_9H_{11}Re(CO)_2NO$ structures

Table S6. Orbital energies and HOMO-LUMO gaps.

Complete Gaussian09 Reference.

Gaussian 09, Revision E.01, M. J. Frisch, G. W. Trucks, H. B. Schlegel, G. E. Scuseria, M. A. Robb, J. R. Cheeseman, G. Scalmani, V. Barone, B. Mennucci, G. A. Petersson, H. Nakatsuji, M. Caricato, X. Li, H. P. Hratchian, A. F. Izmaylov, J. Bloino, G. Zheng, J. L. Sonnenberg, M. Hada, M. Ehara, K. Toyota, R. Fukuda, J. Hasegawa, M. Ishida, T. Nakajima, Y. Honda, O. Kitao, H. Nakai, T. Vreven, J. A. Montgomery, Jr., J. E. Peralta, F. Ogliaro, M. Bearpark, J. J. Heyd, E. Brothers, K. N. Kudin, V. N. Staroverov, R. Kobayashi, J. Normand, K. Raghavachari, A. Rendell, J. C. Burant, S. S. Iyengar, J. Tomasi, M. Cossi, N. Rega, J. M. Millam, M. Klene, J. E. Knox, J. B. Cross, V. Bakken, C. Adamo, J. Jaramillo, R. Gomperts, R. E. Stratmann, O. Yazyev, A. J. Austin, R. Cammi, C. Pomelli, J. W. Ochterski, R. L. Martin, K. Morokuma, V. G. Zakrzewski, G. A. Voth, P. Salvador, J. J. Dannenberg, S. Dapprich, A. D. Daniels, O. Farkas, J. B. Foresman, J. V. Ortiz, J. Cioslowski, and D. J. Fox, Gaussian, Inc., Wallingford CT, 2009.

Table 1A. Initial $(CO)_2(NO)ReC_2B_5H_7$ structures (one example from each family), a total of 414 structures:

**Initial structures**	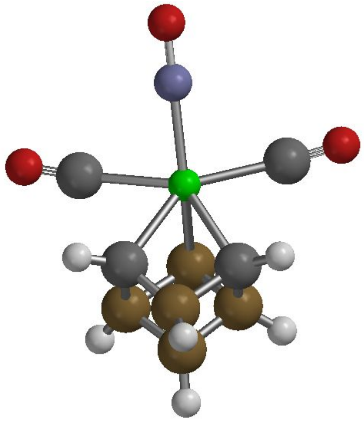<br>1. Cube 18	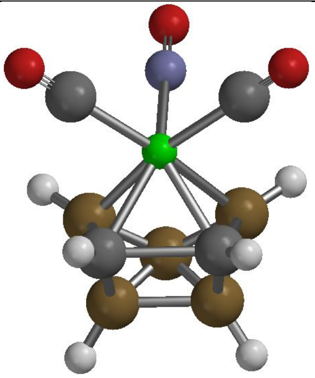<br>2. Antiprism 18
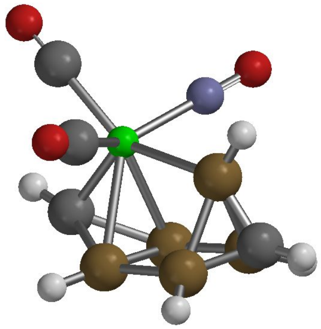<br>3. Trigonal Prism 30	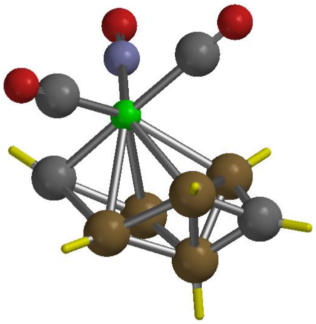<br>4. Trigonal Antiprism 30	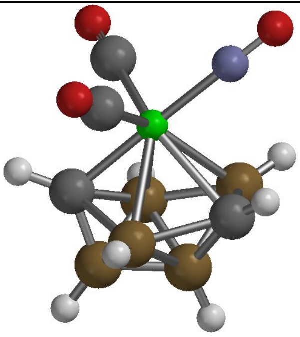<br>5. Bisdisphenoid 54
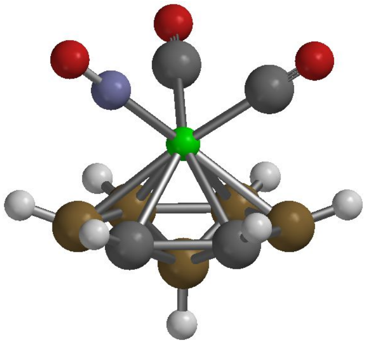<br>6. Hexagonal bipyramid 18	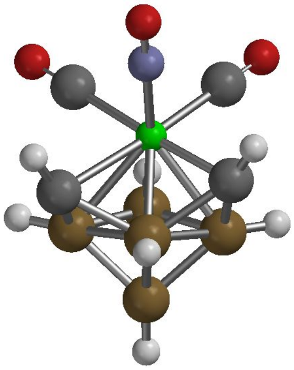<br>7. All-capped tetrahedron 24	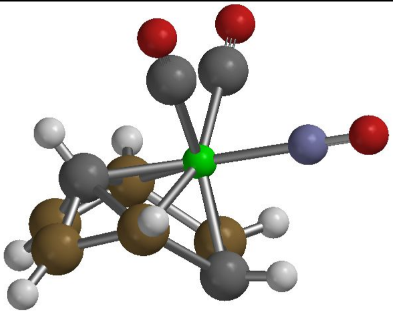<br>8. Nido structure 24

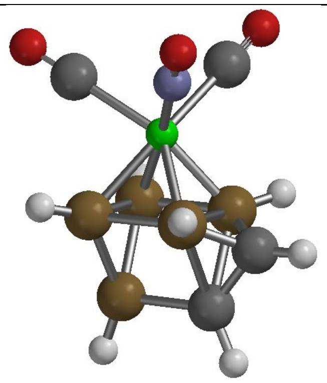 9. Dicapped trigonal prism (C_s) 78	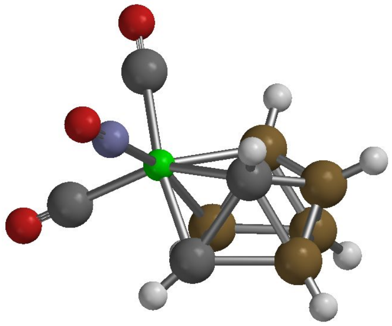 10. Dicapped trigonal prism (C_{2v}) 60	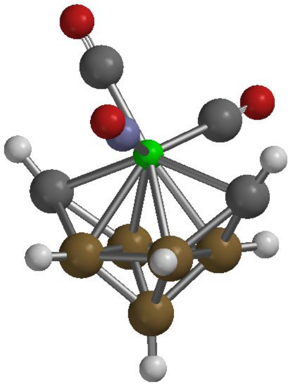 11. Dicapped octahedron 60

Table 1B. Distances table for the lowest-lying $(CO)_2(NO)ReC_2B_5H_7$ structures after M06L/6-311G(d,p)//SDD optimization. Included are the ZPcorrected E (a.u.), relative energy (kcal/mol) and symmetry.

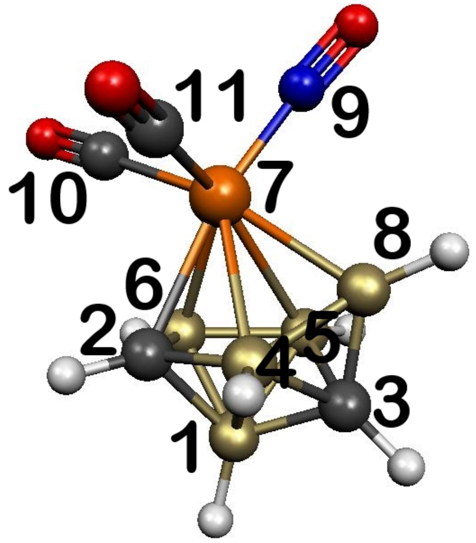

1. -639.7933868 0.0 C_1

		1	2	3	4	5
1	B	0.000000				
2	C	1.662868	0.000000			
3	C	1.579571	2.596306	0.000000		
4	B	1.862540	1.607041	1.676319	0.000000	
5	B	1.843895	2.522485	1.687585	2.507566	0.000000
6	B	1.764105	1.554551	2.732890	2.628332	1.708130
7	Re	3.153242	2.229384	3.112291	2.400846	2.465428
8	B	2.642697	2.861645	1.504509	1.839810	1.855438
9	N	4.658683	4.036178	4.178545	3.886854	3.369701
10	C	4.215713	3.007260	4.758328	4.009969	3.694294
11	C	4.429144	3.096906	4.351054	3.007762	4.280193

		6	7	8	9	10
6	B	0.000000				
7	Re	2.294307	0.000000			
8	B	2.977610	2.211991	0.000000		
9	N	3.763752	1.833465	2.848197	0.000000	
10	C	2.674165	2.025590	4.150087	2.900759	0.000000
11	C	3.912823	1.988705	3.415013	2.850481	2.912291

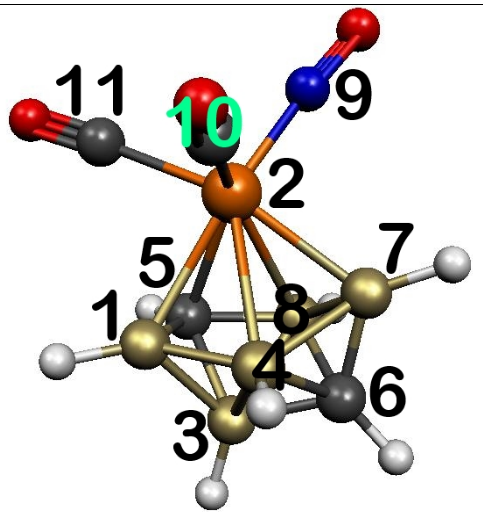

		1	2	3	4	5
1	B	0.000000				
2	Re	2.307573	0.000000			
3	B	1.759236	3.162291	0.000000		
4	B	1.715930	2.441014	1.846480	0.000000	
5	C	1.552925	2.239088	1.671357	2.516001	0.000000
6	C	2.738255	3.110677	1.582396	1.690299	2.594419
7	B	2.992620	2.214439	2.651180	1.840813	2.869155
8	B	2.625537	2.426983	1.854594	2.504552	1.591219
9	N	4.120061	1.839405	4.662494	3.995819	3.657720

2. -639.7864854 +4.3 C_1

10	C	3.235520	1.975203	4.444218	2.984773	3.931672
11	C	2.882940	2.026454	4.234208	3.942755	2.813511

		6	7	8	9	10
6	C	0.000000				
7	B	1.503642	0.000000			
8	B	1.675185	1.873716	0.000000		
9	N	4.211207	2.941715	3.303419	0.000000	
10	C	4.245209	3.201514	4.182454	2.815561	0.000000
11	C	4.785339	4.191950	3.802279	2.938269	2.893907

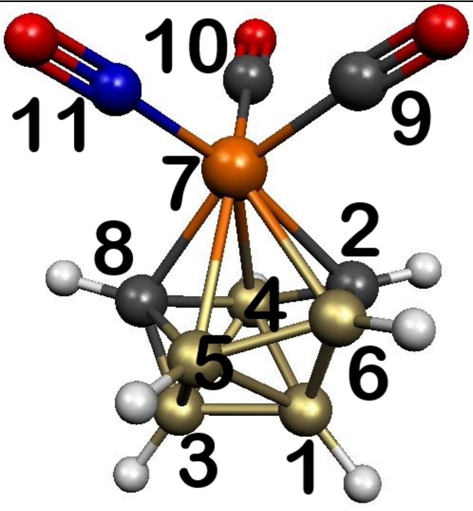

3. -639.7818707 +7.2 C_1

		1	2	3	4	5
1	B	0.000000				
2	C	1.666707	0.000000			
3	B	1.674141	2.740979	0.000000		
4	B	1.865514	1.603793	1.794258	0.000000	
5	B	1.918630	2.519965	1.823176	2.485724	0.000000
6	B	1.783711	1.547802	2.877096	2.617789	1.721329
7	Re	3.174765	2.227064	3.291670	2.410841	2.396656
8	C	2.492710	2.695548	1.506311	1.698391	1.690879
9	C	4.279281	3.009059	4.972813	3.998520	3.733781
10	C	4.459243	3.149764	4.469647	3.000254	4.199199
11	N	4.693261	4.034710	4.382748	3.950541	3.281767

		6	7	8	9	10
6	B	0.000000				
7	Re	2.271702	0.000000			
8	C	2.818945	2.165021	0.000000		
9	C	2.745983	2.008025	4.096619	0.000000	
10	C	3.939388	1.991417	3.325465	2.877790	0.000000
11	N	3.703543	1.834874	2.984288	2.812614	2.845048

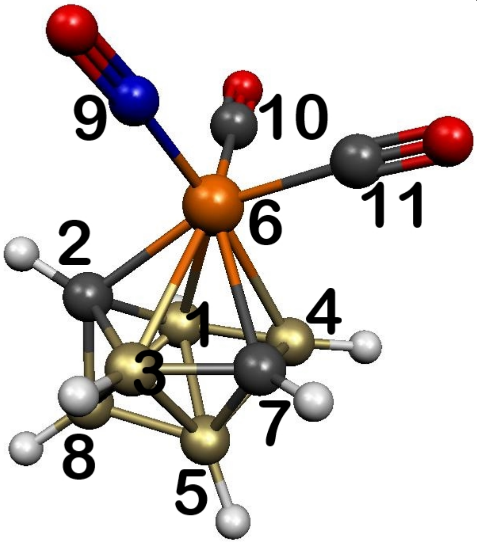

4. -639.7758567 +11.0 C_1

		1	2	3	4	5
1	B	0.000000				
2	C	1.682887	0.000000			
3	B	2.483510	1.709424	0.000000		
4	B	1.723115	2.826828	2.617249	0.000000	
5	B	1.914551	2.493823	1.873074	1.777295	0.000000
6	Re	2.400973	2.170123	2.407454	2.299700	3.188320
7	C	2.509908	2.697121	1.598915	1.541161	1.674550
8	B	1.825962	1.504922	1.802480	2.879380	1.673557
9	N	3.949024	3.003740	3.310373	4.121193	4.704523
10	C	2.940278	3.252961	4.168150	3.192694	4.464027
11	C	3.967107	4.116443	3.803130	2.972744	4.317002

		6	7	8	9	10
6	Re	0.000000				
7	C	2.228816	0.000000			
8	B	3.298654	2.742135	0.000000		
9	N	1.843054	3.676466	4.391875	0.000000	
10	C	1.972619	3.897413	4.426757	2.816874	0.000000
11	C	2.006175	2.864416	5.001228	2.844715	2.835205

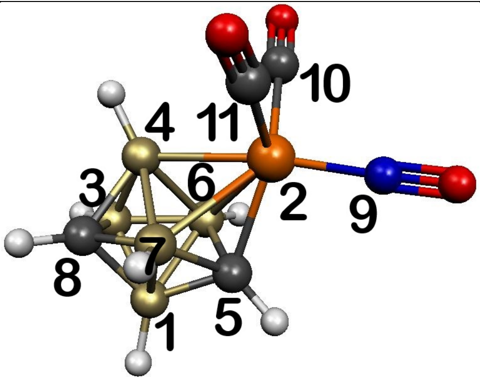

5. -639.7717321 +13.6 C_1

		1	2	3	4	5
1	B	0.000000				
2	Re	3.334754	0.000000			
3	B	1.779317	3.563515	0.000000		
4	B	2.487402	2.296187	1.775147	0.000000	
5	C	1.600117	2.083570	2.734113	2.518806	0.000000
6	B	1.854252	2.393255	1.691891	1.921475	1.676875
7	B	1.835134	2.380470	2.614615	1.896842	1.661050
8	C	1.654887	3.431570	1.523603	1.665201	2.587838
9	N	4.616720	1.851510	5.197108	4.133926	3.050258
10	C	4.668976	1.971532	4.162318	2.906215	3.728206

11	C	4.652035	1.963185	4.754363	3.061401	3.651762

		6	7	8	9	10
6	B	0.000000				
7	B	2.477772	0.000000			
8	C	2.471624	1.599108	0.000000		
9	N	3.734513	3.816396	5.103824	0.000000	
10	C	3.128156	3.996790	4.505449	2.893042	0.000000
11	C	4.092490	3.059469	4.147241	2.859780	2.756876

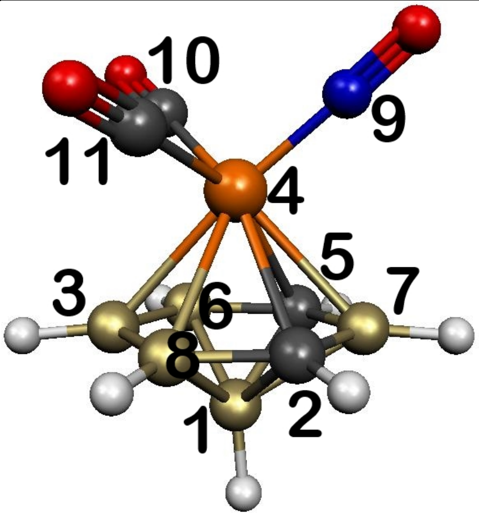

6. -639.7494726 +27.6 C_S

		1	2	3	4	5
1	B	0.000000				
2	C	1.793564	0.000000			
3	B	1.868487	2.679580	0.000000		
4	Re	2.778705	2.333890	2.366865	0.000000	
5	C	1.794398	2.552089	2.679190	2.334837	0.000000
6	B	1.876632	3.096621	1.646715	2.410060	1.529465
7	B	1.921961	1.530791	3.200534	2.395833	1.530410
8	B	1.875795	1.530026	1.646773	2.408833	3.096574
9	N	4.230703	3.323241	4.205103	1.846175	3.323826
10	C	4.069501	4.219893	3.051107	1.978398	3.254704
11	C	4.072773	3.256134	3.057112	1.978168	4.222067

		6	7	8	9	10
6	B	0.000000				
7	B	2.760852	0.000000			
8	B	2.840337	2.761398	0.000000		
9	N	3.956453	2.985325	3.955926	0.000000	
10	C	2.706483	4.028092	3.918117	2.847725	0.000000
11	C	3.922723	4.029008	2.710315	2.843523	2.828640

Table 1C. Energy ranking for $(CO)_2(NO)ReC_2B_5H_7$ obtained after B3LYP/6-31G(d)//SDD optimization:

No	Initial structure	Final energy (a.u.)	ΔE (kcal/mol)
1	02-Antipr---ReC2---20	-639.6086841	0.00
2	10-DicapTrPr-C2v---ReC2---328	-639.6086835	0.00
3	07-Tdallcap---ReC2---181	-639.6086695	0.01
4	07-Tdallcap---ReC2---192	-639.6086695	0.01
5	11-DicapOh---ReC2---400	-639.6086692	0.01
6	11-DicapOh---ReC2---411	-639.6086692	0.01
7	10-DicapTrPr-C2v---ReC2---302	-639.6086691	0.01
8	10-DicapTrPr-C2v---ReC2---333	-639.6086680	0.01
9	11-DicapOh---ReC2---374	-639.6086680	0.01
10	07-Tdallcap---ReC2---169	-639.6086678	0.01
11	10-DicapTrPr-C2v---ReC2---314	-639.6086678	0.01
12	04-AntiprTrig---ReC2---92	-639.6086671	0.01
13	08-Nido---ReC2---202	-639.6086671	0.01
14	10-DicapTrPr-C2v---ReC2---332	-639.6086629	0.01
15	11-DicapOh---ReC2---368	-639.6086606	0.01
16	11-DicapOh---ReC2---412	-639.6086597	0.02
17	05-Bisdisph---ReC2---138	-639.6086553	0.02
18	10-DicapTrPr-C2v---ReC2---306	-639.6086530	0.02
19	10-DicapTrPr-C2v---ReC2---308	-639.6086530	0.02
20	10-DicapTrPr-C2v---ReC2---346	-639.6086493	0.02
21	06-Bipirhex---ReC2---165	-639.6086481	0.02
22	06-Bipirhex---ReC2---158	-639.6086480	0.02
23	11-DicapOh---ReC2---394	-639.6086461	0.02
24	01-Cub---ReC2---11	-639.6086458	0.02
25	11-DicapOh---ReC2---399	-639.6086445	0.02
26	10-DicapTrPr-C2v---ReC2---350	-639.6086407	0.03

27	07-Tdallcap---ReC2---170	-639.6086403	0.03
28	04-AntiprTrig---ReC2---76	-639.6086392	0.03
29	01-Cub---ReC2---15	-639.6086384	0.03
30	10-DicapTrPr-C2v---ReC2---304	-639.6086358	0.03
31	05-Bisdisph---ReC2---148	-639.6086303	0.03
32	02-Antipr---ReC2---33	-639.6086223	0.04
33	05-Bisdisph---ReC2---109	-639.6086197	0.04
34	05-Bisdisph---ReC2---122	-639.6086197	0.04
35	09-DicapTrPr-Cs---ReC2---232	-639.6086176	0.04
36	11-DicapOh---ReC2---362	-639.6086163	0.04
37	10-DicapTrPr-C2v---ReC2---298	-639.6086004	0.05
38	11-DicapOh---ReC2---387	-639.6083232	0.23
39	05-Bisdisph---ReC2---106	-639.6083119	0.23
40	05-Bisdisph---ReC2---119	-639.6083030	0.24
41	01-Cub---ReC2---18	-639.6082997	0.24
42	01-Cub---ReC2---5	-639.6082997	0.24
43	05-Bisdisph---ReC2---120	-639.6082887	0.25
44	05-Bisdisph---ReC2---98	-639.6082887	0.25
45	08-Nido---ReC2---210	-639.6082880	0.25
46	09-DicapTrPr-Cs---ReC2---289	-639.6082863	0.25
47	01-Cub---ReC2---9	-639.6082853	0.25
48	10-DicapTrPr-C2v---ReC2---319	-639.6082790	0.25
49	09-DicapTrPr-Cs---ReC2---287	-639.6082666	0.26
50	10-DicapTrPr-C2v---ReC2---307	-639.6082663	0.26
51	09-DicapTrPr-Cs---ReC2---272	-639.6082650	0.26
52	09-DicapTrPr-Cs---ReC2---221	-639.6082644	0.26
53	09-DicapTrPr-Cs---ReC2---266	-639.6063620	1.46
54	09-DicapTrPr-Cs---ReC2---285	-639.6063620	1.46

55	08-Nido---ReC2---216	-639.6063606	1.46
56	08-Nido---ReC2---200	-639.6063585	1.46
57	04-AntiprTrig---ReC2---75	-639.6063558	1.46
58	04-AntiprTrig---ReC2---82	-639.6063558	1.46
59	04-AntiprTrig---ReC2---85	-639.6063526	1.46
60	06-Bipirhex---ReC2---153	-639.6063455	1.47
61	05-Bisdisph---ReC2---144	-639.6063449	1.47
62	10-DicapTrPr-C2v---ReC2---340	-639.6032211	3.43
63	09-DicapTrPr-Cs---ReC2---226	-639.6032120	3.43
64	09-DicapTrPr-Cs---ReC2---231	-639.6032120	3.43
65	10-DicapTrPr-C2v---ReC2---353	-639.6031834	3.45
66	11-DicapOh---ReC2---402	-639.6031819	3.45
67	05-Bisdisph---ReC2---105	-639.6031817	3.45
68	05-Bisdisph---ReC2---111	-639.6031817	3.45
69	05-Bisdisph---ReC2---112	-639.6031720	3.46
70	04-AntiprTrig---ReC2---77	-639.6031676	3.46
71	04-AntiprTrig---ReC2---95	-639.6031676	3.46
72	11-DicapOh---ReC2---392	-639.6031572	3.47
73	02-Antipr---ReC2---36	-639.6031042	3.50
74	02-Antipr---ReC2---24	-639.5960724	7.91
75	10-DicapTrPr-C2v---ReC2---339	-639.5960696	7.92
76	01-Cub---ReC2---12	-639.5960508	7.93
77	01-Cub---ReC2---1	-639.5960508	7.93
78	10-DicapTrPr-C2v---ReC2---347	-639.5960327	7.94
79	09-DicapTrPr-Cs---ReC2---224	-639.5960325	7.94
80	10-DicapTrPr-C2v---ReC2---320	-639.5960316	7.94
81	05-Bisdisph---ReC2---129	-639.5960245	7.94
82	07-Tdallcap---ReC2---171	-639.5960238	7.94

83	10-DicapTrPr-C2v---ReC2---295	-639.5960222	7.95
84	09-DicapTrPr-Cs---ReC2---273	-639.5960115	7.95
85	09-DicapTrPr-Cs---ReC2---293	-639.5960115	7.95
86	08-Nido---ReC2---194	-639.5960079	7.95
87	04-AntiprTrig---ReC2---72	-639.5960013	7.96
88	04-AntiprTrig---ReC2---96	-639.5960013	7.96
89	05-Bisdisph---ReC2---126	-639.5953245	8.38
90	05-Bisdisph---ReC2---97	-639.5953245	8.38
91	11-DicapOh---ReC2---397	-639.5953194	8.39
92	09-DicapTrPr-Cs---ReC2---219	-639.5953041	8.40
93	06-Bipirhex---ReC2---168	-639.5952952	8.40
94	10-DicapTrPr-C2v---ReC2---348	-639.5952943	8.40
95	05-Bisdisph---ReC2---125	-639.5952925	8.40
96	05-Bisdisph---ReC2---127	-639.5952792	8.41
97	05-Bisdisph---ReC2---133	-639.5952792	8.41
98	08-Nido---ReC2---208	-639.5952784	8.41
99	11-DicapOh---ReC2---404	-639.5906128	11.34
100	10-DicapTrPr-C2v---ReC2---341	-639.5906120	11.34
101	09-DicapTrPr-Cs---ReC2---217	-639.5906064	11.34
102	08-Nido---ReC2---206	-639.5905894	11.35
103	05-Bisdisph---ReC2---107	-639.5905834	11.36
104	06-Bipirhex---ReC2---164	-639.5905822	11.36
105	04-AntiprTrig---ReC2---74	-639.5905704	11.37
106	08-Nido---ReC2---213	-639.5902934	11.54
107	09-DicapTrPr-Cs---ReC2---267	-639.5902727	11.55
108	09-DicapTrPr-Cs---ReC2---291	-639.5902727	11.55
109	09-DicapTrPr-Cs---ReC2---228	-639.5902438	11.57
110	05-Bisdisph---ReC2---130	-639.5902432	11.57

111	05-Bisdisph---ReC2---139	-639.5902432	11.57
112	03-PrTrig---ReC2---41	-639.5902430	11.57
113	05-Bisdisph---ReC2---101	-639.5902150	11.59
114	09-DicapTrPr-Cs---ReC2---270	-639.5824022	16.49
115	09-DicapTrPr-Cs---ReC2---280	-639.5824022	16.49
116	11-DicapOh---ReC2---410	-639.5823954	16.50
117	08-Nido---ReC2---193	-639.5823940	16.50
118	08-Nido---ReC2---212	-639.5823940	16.50
119	05-Bisdisph---ReC2---141	-639.5823574	16.52
120	02-Antipr---ReC2---28	-639.5819236	16.79
121	10-DicapTrPr-C2v---ReC2---297	-639.5819088	16.80
122	10-DicapTrPr-C2v---ReC2---317	-639.5819079	16.80
123	11-DicapOh---ReC2---409	-639.5818982	16.81
124	10-DicapTrPr-C2v---ReC2---323	-639.5818772	16.82
125	10-DicapTrPr-C2v---ReC2---329	-639.5818772	16.82
126	06-Bipirhex---ReC2---152	-639.5818751	16.82
127	05-Bisdisph---ReC2---137	-639.5818659	16.83
128	05-Bisdisph---ReC2---146	-639.5818659	16.83
129	02-Antipr---ReC2---22	-639.5818531	16.84
130	02-Antipr---ReC2---26	-639.5818531	16.84
131	01-Cub---ReC2---3	-639.5786855	18.82
132	09-DicapTrPr-Cs---ReC2---281	-639.5786811	18.83
133	07-Tdallcap---ReC2---191	-639.5786670	18.84
134	11-DicapOh---ReC2---383	-639.5786632	18.84
135	11-DicapOh---ReC2---377	-639.5786550	18.84
136	09-DicapTrPr-Cs---ReC2---248	-639.5786268	18.86
137	04-AntiprTrig---ReC2---70	-639.5786062	18.87
138	11-DicapOh---ReC2---378	-639.5786007	18.88

139	11-DicapOh---ReC2---384	-639.5786007	18.88
140	03-PrTrig---ReC2---38	-639.5785965	18.88
141	11-DicapOh---ReC2---403	-639.5785869	18.89
142	08-Nido---ReC2---195	-639.5785824	18.89
143	08-Nido---ReC2---196	-639.5785794	18.89
144	08-Nido---ReC2---207	-639.5785794	18.89
145	07-Tdallcap---ReC2---186	-639.5785725	18.90
146	09-DicapTrPr-Cs---ReC2---282	-639.5785631	18.90
147	09-DicapTrPr-Cs---ReC2---276	-639.5785564	18.91
148	07-Tdallcap---ReC2---184	-639.5785560	18.91
149	07-Tdallcap---ReC2---189	-639.5785543	18.91
150	04-AntiprTrig---ReC2---67	-639.5762471	20.35
151	04-AntiprTrig---ReC2---90	-639.5762471	20.35
152	11-DicapOh---ReC2---386	-639.5762400	20.36
153	06-Bipirhex---ReC2---161	-639.5762396	20.36
154	09-DicapTrPr-Cs---ReC2---275	-639.5761900	20.39
155	09-DicapTrPr-Cs---ReC2---249	-639.5742527	21.61
156	04-AntiprTrig---ReC2---80	-639.5733369	22.18
157	03-PrTrig---ReC2---49	-639.5733247	22.19
158	11-DicapOh---ReC2---381	-639.5732889	22.21
159	09-DicapTrPr-Cs---ReC2---225	-639.5728677	22.48
160	05-Bisdisph---ReC2---135	-639.5728634	22.48
161	09-DicapTrPr-Cs---ReC2---279	-639.5728591	22.48
162	09-DicapTrPr-Cs---ReC2---294	-639.5728591	22.48
163	10-DicapTrPr-C2v---ReC2---337	-639.5728586	22.48
164	10-DicapTrPr-C2v---ReC2---345	-639.5728586	22.48
165	11-DicapOh---ReC2---401	-639.5728454	22.49
166	11-DicapOh---ReC2---405	-639.5728454	22.49

167	02-Antipr---ReC2---23	-639.5728442	22.49
168	05-Bisdisph---ReC2---136	-639.5728429	22.49
169	05-Bisdisph---ReC2---140	-639.5728429	22.49
170	10-DicapTrPr-C2v---ReC2---299	-639.5728373	22.49
171	10-DicapTrPr-C2v---ReC2---301	-639.5728243	22.50
172	10-DicapTrPr-C2v---ReC2---342	-639.5724829	22.72
173	11-DicapOh---ReC2---398	-639.5724767	22.72
174	08-Nido---ReC2---205	-639.5724630	22.73
175	08-Nido---ReC2---214	-639.5724630	22.73
176	09-DicapTrPr-Cs---ReC2---222	-639.5724496	22.74
177	11-DicapOh---ReC2---388	-639.5724483	22.74
178	03-PrTrig---ReC2---48	-639.5724438	22.74
179	03-PrTrig---ReC2---40	-639.5724418	22.74
180	09-DicapTrPr-Cs---ReC2---269	-639.5724411	22.74
181	09-DicapTrPr-Cs---ReC2---274	-639.5724411	22.74
182	10-DicapTrPr-C2v---ReC2---324	-639.5724048	22.77
183	05-Bisdisph---ReC2---102	-639.5715084	23.33
184	11-DicapOh---ReC2---373	-639.5713124	23.45
185	11-DicapOh---ReC2---389	-639.5713124	23.45
186	10-DicapTrPr-C2v---ReC2---327	-639.5659984	26.79
187	10-DicapTrPr-C2v---ReC2---334	-639.5659984	26.79
188	02-Antipr---ReC2---25	-639.5659947	26.79
189	02-Antipr---ReC2---34	-639.5659947	26.79
190	02-Antipr---ReC2---27	-639.5659903	26.79
191	06-Bipirhex---ReC2---167	-639.5659892	26.79
192	10-DicapTrPr-C2v---ReC2---315	-639.5659852	26.79
193	11-DicapOh---ReC2---414	-639.5659768	26.80
194	05-Bisdisph---ReC2---150	-639.5659759	26.80

195	10-DicapTrPr-C2v---ReC2---325	-639.5659731	26.80
196	09-DicapTrPr-Cs---ReC2---239	-639.5638904	28.11
197	09-DicapTrPr-Cs---ReC2---263	-639.5638904	28.11
198	07-Tdallcap---ReC2---182	-639.5625954	28.92
199	09-DicapTrPr-Cs---ReC2---254	-639.5625887	28.93
200	07-Tdallcap---ReC2---187	-639.5625880	28.93
201	02-Antipr---ReC2---35	-639.5625613	28.94
202	01-Cub---ReC2---8	-639.5625604	28.94
203	09-DicapTrPr-Cs---ReC2---271	-639.5625476	28.95
204	09-DicapTrPr-Cs---ReC2---284	-639.5625465	28.95
205	09-DicapTrPr-Cs---ReC2---290	-639.5625465	28.95
206	05-Bisdisph---ReC2---121	-639.5625440	28.95
207	07-Tdallcap---ReC2---180	-639.5625439	28.95
208	07-Tdallcap---ReC2---185	-639.5625434	28.95
209	05-Bisdisph---ReC2---115	-639.5625433	28.95
210	11-DicapOh---ReC2---407	-639.5625081	28.98
211	11-DicapOh---ReC2---413	-639.5625081	28.98
212	04-AntiprTrig---ReC2---87	-639.5610890	29.87
213	10-DicapTrPr-C2v---ReC2---338	-639.5610571	29.89
214	10-DicapTrPr-C2v---ReC2---351	-639.5610571	29.89
215	01-Cub---ReC2---7	-639.5610327	29.90
216	08-Nido---ReC2---203	-639.5610317	29.90
217	08-Nido---ReC2---209	-639.5610317	29.90
218	09-DicapTrPr-Cs---ReC2---234	-639.5610022	29.92
219	02-Antipr---ReC2---19	-639.5598803	30.63
220	02-Antipr---ReC2---31	-639.5598803	30.63
221	05-Bisdisph---ReC2---134	-639.5598761	30.63
222	08-Nido---ReC2---198	-639.5598737	30.63

223	10-DicapTrPr-C2v---ReC2---343	-639.5598572	30.64
224	10-DicapTrPr-C2v---ReC2---349	-639.5598572	30.64
225	10-DicapTrPr-C2v---ReC2---303	-639.5598483	30.65
226	09-DicapTrPr-Cs---ReC2---278	-639.5598477	30.65
227	09-DicapTrPr-Cs---ReC2---288	-639.5598477	30.65
228	09-DicapTrPr-Cs---ReC2---277	-639.5598431	30.65
229	10-DicapTrPr-C2v---ReC2---312	-639.5598418	30.65
230	10-DicapTrPr-C2v---ReC2---300	-639.5598413	30.65
231	08-Nido---ReC2---197	-639.5598276	30.66
232	08-Nido---ReC2---201	-639.5598276	30.66
233	06-Bipirhex---ReC2---155	-639.5589710	31.20
234	11-DicapOh---ReC2---375	-639.5589628	31.20
235	11-DicapOh---ReC2---376	-639.5589361	31.22
236	11-DicapOh---ReC2---382	-639.5589241	31.23
237	09-DicapTrPr-Cs---ReC2---220	-639.5583408	31.59
238	09-DicapTrPr-Cs---ReC2---229	-639.5583408	31.59
239	10-DicapTrPr-C2v---ReC2---354	-639.5583239	31.60
240	09-DicapTrPr-Cs---ReC2---230	-639.5583212	31.60
241	05-Bisdisph---ReC2---114	-639.5583155	31.61
242	05-Bisdisph---ReC2---99	-639.5583155	31.61
243	05-Bisdisph---ReC2---113	-639.5583125	31.61
244	04-AntiprTrig---ReC2---73	-639.5582986	31.62
245	04-AntiprTrig---ReC2---88	-639.5582986	31.62
246	04-AntiprTrig---ReC2---79	-639.5577768	31.95
247	04-AntiprTrig---ReC2---86	-639.5577768	31.95
248	10-DicapTrPr-C2v---ReC2---311	-639.5577661	31.95
249	08-Nido---ReC2---199	-639.5577570	31.96
250	08-Nido---ReC2---215	-639.5577570	31.96

251	09-DicapTrPr-Cs---ReC2---265	-639.5577563	31.96
252	09-DicapTrPr-Cs---ReC2---227	-639.5577440	31.97
253	05-Bisdisph---ReC2---104	-639.5577432	31.97
254	05-Bisdisph---ReC2---117	-639.5577432	31.97
255	05-Bisdisph---ReC2---118	-639.5577211	31.98
256	09-DicapTrPr-Cs---ReC2---235	-639.5526149	35.18
257	09-DicapTrPr-Cs---ReC2---256	-639.5526149	35.18
258	09-DicapTrPr-Cs---ReC2---247	-639.5525967	35.20
259	09-DicapTrPr-Cs---ReC2---253	-639.5525967	35.20
260	09-DicapTrPr-Cs---ReC2---241	-639.5520859	35.52
261	09-DicapTrPr-Cs---ReC2---258	-639.5520395	35.55
262	05-Bisdisph---ReC2---124	-639.5520341	35.55
263	05-Bisdisph---ReC2---103	-639.5520234	35.56
264	05-Bisdisph---ReC2---123	-639.5520234	35.56
265	09-DicapTrPr-Cs---ReC2---261	-639.5514792	35.90
266	05-Bisdisph---ReC2---100	-639.5508780	36.27
267	05-Bisdisph---ReC2---108	-639.5508780	36.27
268	10-DicapTrPr-C2v---ReC2---296	-639.5501045	36.76
269	10-DicapTrPr-C2v---ReC2---305	-639.5501045	36.76
270	11-DicapOh---ReC2---406	-639.5500968	36.76
271	10-DicapTrPr-C2v---ReC2---352	-639.5499502	36.86
272	11-DicapOh---ReC2---363	-639.5499176	36.88
273	01-Cub---ReC2---2	-639.5499088	36.88
274	09-DicapTrPr-Cs---ReC2---283	-639.5485028	37.76
275	05-Bisdisph---ReC2---110	-639.5484722	37.78
276	05-Bisdisph---ReC2---116	-639.5484722	37.78
277	10-DicapTrPr-C2v---ReC2---330	-639.5484073	37.82
278	05-Bisdisph---ReC2---132	-639.5484059	37.83

279	09-DicapTrPr-Cs---ReC2---244	-639.5483736	37.85
280	10-DicapTrPr-C2v---ReC2---313	-639.5481382	37.99
281	10-DicapTrPr-C2v---ReC2---336	-639.5481182	38.01
282	02-Antipr---ReC2---30	-639.5481127	38.01
283	02-Antipr---ReC2---29	-639.5481080	38.01
284	05-Bisdisph---ReC2---131	-639.5480879	38.03
285	05-Bisdisph---ReC2---145	-639.5480879	38.03
286	09-DicapTrPr-Cs---ReC2---268	-639.5477111	38.26
287	10-DicapTrPr-C2v---ReC2---316	-639.5477087	38.26
288	10-DicapTrPr-C2v---ReC2---326	-639.5477082	38.26
289	10-DicapTrPr-C2v---ReC2---321	-639.5476967	38.27
290	10-DicapTrPr-C2v---ReC2---335	-639.5476967	38.27
291	05-Bisdisph---ReC2---143	-639.5476854	38.28
292	05-Bisdisph---ReC2---149	-639.5476854	38.28
293	08-Nido---ReC2---204	-639.5474897	38.40
294	11-DicapOh---ReC2---367	-639.5474594	38.42
295	09-DicapTrPr-Cs---ReC2---243_r-211	-639.5468776	38.78
296	02-Antipr---ReC2---21	-639.5468695	38.79
297	10-DicapTrPr-C2v---ReC2---344	-639.5468113	38.83
298	01-Cub---ReC2---10	-639.5463563	39.11
299	01-Cub---ReC2---13	-639.5463563	39.11
300	09-DicapTrPr-Cs---ReC2---233	-639.5462240	39.19
301	06-Bipirhex---ReC2---156	-639.5453751	39.73
302	06-Bipirhex---ReC2---151	-639.5453633	39.74
303	09-DicapTrPr-Cs---ReC2---243_i-211	-639.5452113	39.83
304	08-Nido---ReC2---211	-639.5440913	40.53
305	09-DicapTrPr-Cs---ReC2---218	-639.5440852	40.54
306	09-DicapTrPr-Cs---ReC2---223	-639.5440852	40.54

307	10-DicapTrPr-C2v---ReC2---331	-639.5432526	41.06
308	02-Antipr---ReC2---32	-639.5426748	41.42
309	04-AntiprTrig---ReC2---81	-639.5426675	41.43
310	10-DicapTrPr-C2v---ReC2---310	-639.5426557	41.43
311	10-DicapTrPr-C2v---ReC2---309	-639.5418945	41.91
312	10-DicapTrPr-C2v---ReC2---318	-639.5418922	41.91
313	05-Bisdisph---ReC2---147	-639.5418644	41.93
314	07-Tdallcap---ReC2---188	-639.5403109	42.91
315	03-PrTrig---ReC2---42	-639.5362880	45.43
316	03-PrTrig---ReC2---66	-639.5362880	45.43
317	09-DicapTrPr-Cs---ReC2---236	-639.5355257	45.91
318	09-DicapTrPr-Cs---ReC2---250	-639.5355257	45.91
319	07-Tdallcap---ReC2---190	-639.5348751	46.32
320	03-PrTrig---ReC2---56	-639.5347937	46.37
321	03-PrTrig---ReC2---52	-639.5347708	46.38
322	03-PrTrig---ReC2---57	-639.5347708	46.38
323	11-DicapOh---ReC2---372	-639.5347653	46.39
324	11-DicapOh---ReC2---359	-639.5347597	46.39
325	11-DicapOh---ReC2---371	-639.5347597	46.39
326	01-Cub---ReC2---4	-639.5338364	46.97
327	04-AntiprTrig---ReC2---94	-639.5332223	47.35
328	07-Tdallcap---ReC2---174	-639.5332126	47.36
329	07-Tdallcap---ReC2---183	-639.5332119	47.36
330	04-AntiprTrig---ReC2---68	-639.5300272	49.36
331	04-AntiprTrig---ReC2---71	-639.5294939	49.69
332	04-AntiprTrig---ReC2---78	-639.5294939	49.69
333	10-DicapTrPr-C2v---ReC2---322	-639.5285039	50.31
334	11-DicapOh---ReC2---408	-639.5268551	51.35

335	01-Cub---ReC2---6	-639.5254378	52.24
336	07-Tdallcap---ReC2---173	-639.5254199	52.25
337	04-AntiprTrig---ReC2---89	-639.5254145	52.25
338	04-AntiprTrig---ReC2---91	-639.5254145	52.25
339	05-Bisdisph---ReC2---142	-639.5253079	52.32
340	03-PrTrig---ReC2---61	-639.5194845	55.97
341	09-DicapTrPr-Cs---ReC2---246	-639.5166929	57.73
342	09-DicapTrPr-Cs---ReC2---264	-639.5166929	57.73
343	03-PrTrig---ReC2---37	-639.5157045	58.35
344	11-DicapOh---ReC2---361	-639.5154256	58.52
345	09-DicapTrPr-Cs---ReC2---257	-639.5152949	58.60
346	09-DicapTrPr-Cs---ReC2---259	-639.5152949	58.60
347	09-DicapTrPr-Cs---ReC2---262	-639.5152519	58.63
348	09-DicapTrPr-Cs---ReC2---251	-639.5141872	59.30
349	09-DicapTrPr-Cs---ReC2---260	-639.5141872	59.30
350	09-DicapTrPr-Cs---ReC2---242	-639.5132109	59.91
351	09-DicapTrPr-Cs---ReC2---252	-639.5132109	59.91
352	03-PrTrig---ReC2---59	-639.5130386	60.02
353	03-PrTrig---ReC2---65	-639.5130386	60.02
354	11-DicapOh---ReC2---357	-639.5123448	60.45
355	11-DicapOh---ReC2---360	-639.5122064	60.54
356	03-PrTrig---ReC2---62	-639.5108493	61.39
357	11-DicapOh---ReC2---369	-639.5108489	61.39
358	06-Bipirhex---ReC2---163	-639.5107590	61.45
359	05-Bisdisph---ReC2---128	-639.5107461	61.46
360	06-Bipirhex---ReC2---162	-639.5103929	61.68
361	06-Bipirhex---ReC2---166	-639.5103928	61.68
362	06-Bipirhex---ReC2---157	-639.5103545	61.70

363	03-PrTrig---ReC2---44	-639.5093014	62.36
364	11-DicapOh---ReC2---379	-639.5082270	63.04
365	11-DicapOh---ReC2---390	-639.5082270	63.04
366	09-DicapTrPr-Cs---ReC2---292	-639.5074078	63.55
367	09-DicapTrPr-Cs---ReC2---238	-639.5053759	64.83
368	07-Tdallcap---ReC2---176	-639.5048562	65.15
369	07-Tdallcap---ReC2---172	-639.5048462	65.16
370	07-Tdallcap---ReC2---177	-639.5048462	65.16
371	04-AntiprTrig---ReC2---83	-639.5048387	65.17
372	04-AntiprTrig---ReC2---93	-639.5048387	65.17
373	01-Cub---ReC2---17	-639.5048351	65.17
374	03-PrTrig---ReC2---53	-639.5035825	65.95
375	03-PrTrig---ReC2---63	-639.5035825	65.95
376	11-DicapOh---ReC2---364	-639.5035741	65.96
377	11-DicapOh---ReC2---370	-639.5035741	65.96
378	06-Bipirhex---ReC2---159	-639.5030267	66.30
379	03-PrTrig---ReC2---39	-639.5021792	66.83
380	03-PrTrig---ReC2---54	-639.5021792	66.83
381	04-AntiprTrig---ReC2---69	-639.5021775	66.84
382	04-AntiprTrig---ReC2---84	-639.5021775	66.84
383	01-Cub---ReC2---14	-639.5021448	66.86
384	09-DicapTrPr-Cs---ReC2---286	-639.5018662	67.03
385	11-DicapOh---ReC2---391	-639.5013939	67.33
386	11-DicapOh---ReC2---380	-639.5013720	67.34
387	11-DicapOh---ReC2---395	-639.5013720	67.34
388	11-DicapOh---ReC2---358	-639.5001805	68.09
389	11-DicapOh---ReC2---365	-639.5001805	68.09
390	07-Tdallcap---ReC2---178	-639.5000059	68.20

391	07-Tdallcap---ReC2---179	-639.4999283	68.25
392	09-DicapTrPr-Cs---ReC2---240	-639.4989436	68.86
393	09-DicapTrPr-Cs---ReC2---245	-639.4989436	68.86
394	03-PrTrig---ReC2---47	-639.4989023	68.89
395	03-PrTrig---ReC2---64	-639.4989023	68.89
396	06-Bipirhex---ReC2---154	-639.4979405	69.49
397	06-Bipirhex---ReC2---160	-639.4979405	69.49
398	11-DicapOh---ReC2---356	-639.4976477	69.68
399	03-PrTrig---ReC2---55	-639.4976404	69.68
400	09-DicapTrPr-Cs---ReC2---237	-639.4970863	70.03
401	01-Cub---ReC2---16	-639.4954722	71.04
402	11-DicapOh---ReC2---355	-639.4949305	71.38
403	11-DicapOh---ReC2---366	-639.4949262	71.39
404	03-PrTrig---ReC2---45	-639.4913457	73.63
405	03-PrTrig---ReC2---51	-639.4913457	73.63
406	03-PrTrig---ReC2---46	-639.4907272	74.02
407	03-PrTrig---ReC2---58	-639.4907272	74.02
408	07-Tdallcap---ReC2---175	-639.4906962	74.04
409	03-PrTrig---ReC2---60	-639.4900794	74.43
410	09-DicapTrPr-Cs---ReC2---255	-639.4884377	75.46
411	11-DicapOh---ReC2---393	-639.4805940	80.38
412	03-PrTrig---ReC2---50	-639.4689934	87.66
413	11-DicapOh---ReC2---385	-639.4430826	103.92
414	11-DicapOh---ReC2---396	-639.4430826	103.92
415	03-PrTrig---ReC2---43	-639.4426531	104.19

Table 2A. Initial $(CO)_2(NO)ReC_2B_6H_8$ structures (one example from each family), a total of 294 structures:

**Initial structures**
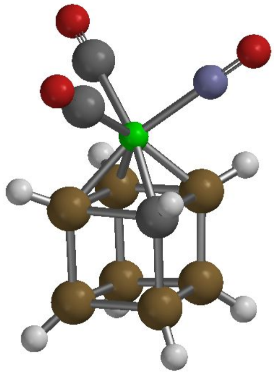 1. Capped cube 49 structures
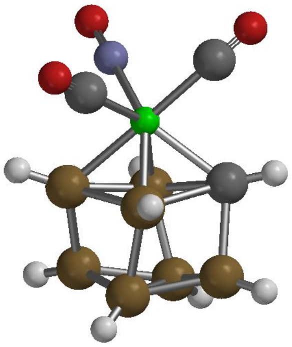 2. Pressed capped cube 77 structures
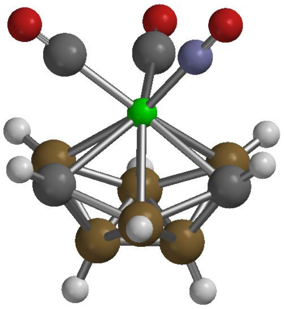 3. Tl_9^{9-}-like structure 105 structures
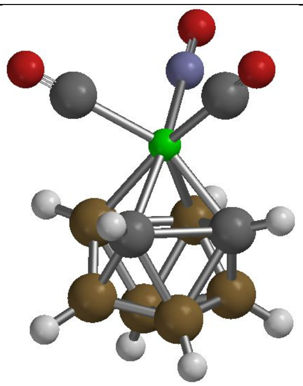

4. Tricapped trigonal prism 63 structures

Table 2B. Distances table for the lowest-lying $(CO)_2(NO)ReC_2B_6H_8$ structures after M06L/6-311G(d,p)//SDD optimization. Included are the ZPcorrected E (a.u.), relative energy (kcal/mol) and symmetry.

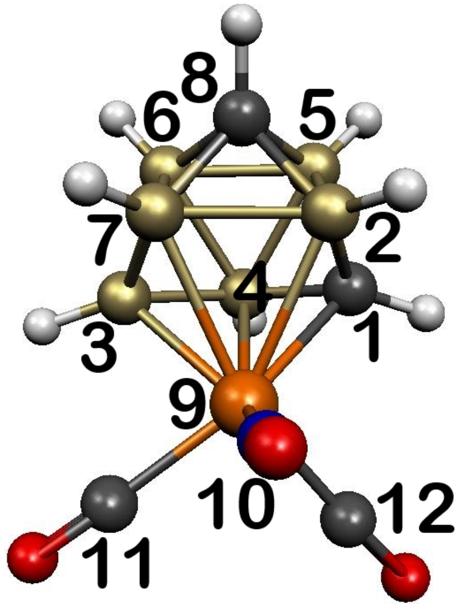

1. -665.2652111 0.0 C_1

		1	2	3	4	5
1	C	0.000000				
2	B	1.606510	0.000000			
3	B	2.799569	2.959078	0.000000		
4	B	1.611148	2.618407	1.751054	0.000000	
5	B	1.610930	1.792801	2.891095	1.893936	0.000000
6	B	2.745634	2.628913	1.697136	1.919718	1.861891
7	B	2.824806	1.995382	1.730235	2.659429	2.627637
8	C	2.557175	1.595226	2.708853	2.727654	1.584502
9	Re	2.159203	2.425438	2.205465	2.372767	3.307733
10	N	3.456295	2.991535	3.656792	4.105598	4.516741
11	C	3.952132	4.278308	2.583934	3.455690	4.899822
12	C	2.827558	3.756665	3.860297	3.312657	4.393324

		6	7	8	9	10
6	B	0.000000				
7	B	1.781243	0.000000			
8	C	1.602554	1.591045	0.000000		
9	Re	3.293521	2.447208	3.358028	0.000000	
10	N	4.586680	3.137886	4.126246	1.841724	0.000000
11	C	4.234546	3.478327	4.803002	1.992354	2.858668
12	C	4.879940	4.391109	4.978607	2.005854	2.888837

		11	12
12	C	2.885432	0.000000

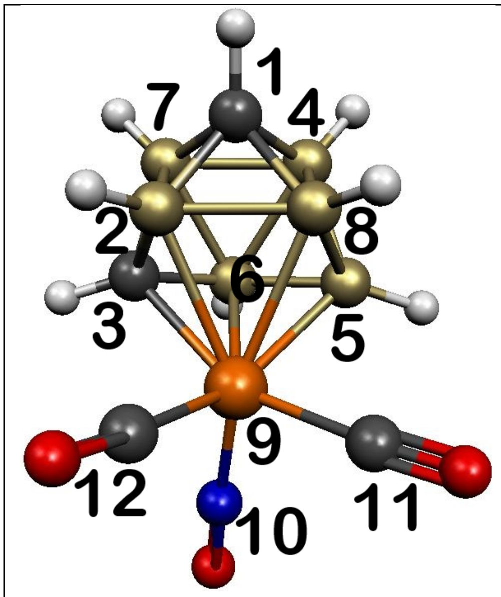

2. -665.2609054 +2.7 C_1

		1	2	3	4	5
1	C	0.000000				
2	B	1.598456	0.000000			
3	C	2.568295	1.632095	0.000000		
4	B	1.591596	2.617000	2.765820	0.000000	
5	B	2.724131	2.951991	2.813538	1.683016	0.000000
6	B	2.748762	2.620526	1.607122	1.964897	1.736347
7	B	1.582413	1.796541	1.594178	1.898788	2.898436
8	B	1.595220	1.913971	2.800340	1.783360	1.769968
9	Re	3.403337	2.448621	2.165614	3.294519	2.209873
10	N	5.070886	4.090231	3.207848	4.667302	3.365337
11	C	4.428008	3.852102	4.072996	4.182215	2.760159
12	C	4.299786	2.857404	3.092510	4.834190	4.088591

		6	7	8	9	10
6	B	0.000000				
7	B	1.923272	0.000000			
8	B	2.655242	2.612904	0.000000		
9	Re	2.310526	3.305969	2.452620	0.000000	
10	N	3.114521	4.587100	4.198428	1.833312	0.000000
11	C	3.840696	4.879526	2.932502	1.997776	2.824336
12	C	4.033374	4.345045	3.580609	2.022181	2.899708

		11	12
12	C	2.911689	0.000000

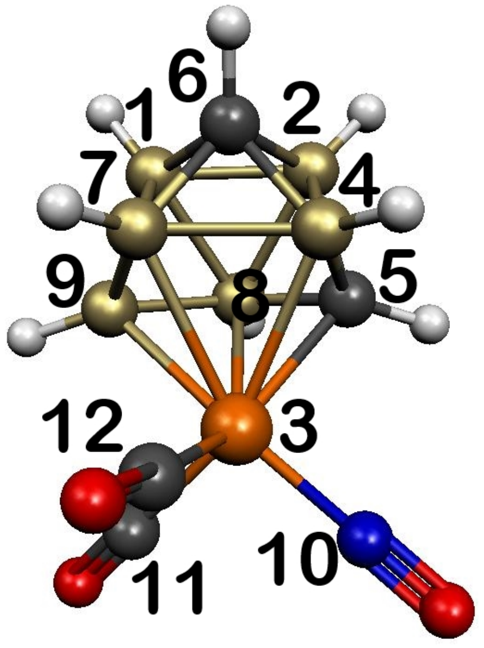

3. -665.2593055 +3.7 C_1

		1	2	3	4	5
1	B	0.000000				
2	B	1.875376	0.000000			
3	Re	3.311815	3.303404	0.000000		
4	B	2.643288	1.796093	2.428069	0.000000	
5	C	2.742085	1.599399	2.143796	1.617963	0.000000
6	C	1.608591	1.584962	3.371127	1.599931	2.553760
7	B	1.776246	2.627358	2.484614	2.002641	2.826972
8	B	1.927514	1.906159	2.363583	2.631138	1.602165
9	B	1.688757	2.899499	2.239462	2.967056	2.804212
10	N	4.974244	4.415275	1.855231	3.415068	2.877741
11	C	4.217756	4.750714	1.978789	4.302804	3.715832
12	C	4.398329	4.719803	1.983720	3.331320	3.852549

		6	7	8	9	10
6	C	0.000000				
7	B	1.586004	0.000000			
8	B	2.734130	2.662625	0.000000		
9	B	2.702444	1.719388	1.763672	0.000000	
10	N	4.790043	4.196009	3.655331	4.043748	0.000000
11	C	4.874555	3.709396	3.150334	2.617345	2.881422
12	C	4.140407	2.865455	4.138263	3.289340	2.832400

		11	12
11	C	0.000000	
12	C	2.840498	0.000000

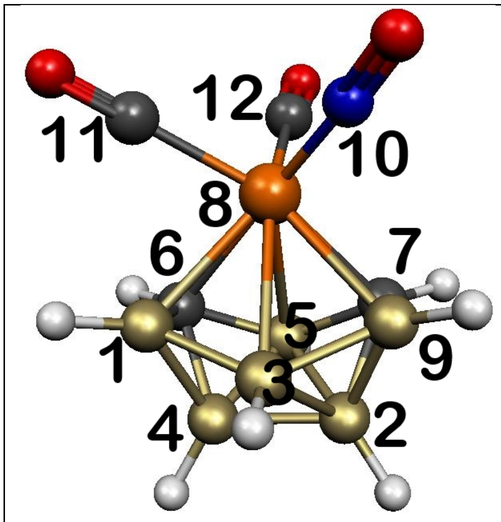

4. -665.2524353 +8.0 (C_s)

		1	2	3	4	5
1	B	0.000000				
2	B	2.887782	0.000000			
3	B	1.728528	1.835907	0.000000		
4	B	1.743943	1.736287	1.835083	0.000000	
5	B	2.698944	1.806797	2.735002	1.807111	0.000000
6	C	1.538428	2.746525	2.587321	1.658435	1.616428
7	C	3.292522	1.662259	2.590832	2.756047	1.628648
8	Re	2.305775	3.087779	2.495121	3.094228	2.573353
9	B	3.059001	1.738221	1.737776	2.888947	2.694941
10	N	3.776424	4.241431	3.470670	4.669848	4.197879
11	C	2.612492	4.765948	3.634312	4.134466	4.100893
12	C	3.901866	4.343096	4.353877	4.390381	3.129386

		6	7	8	9	10
6	C	0.000000				
7	C	2.771922	0.000000			
8	Re	2.249303	2.238242	0.000000		
9	B	3.287653	1.524546	2.297037	0.000000	
10	N	4.073514	3.239776	1.839058	2.755877	0.000000
11	C	2.973544	4.220971	2.022457	4.096280	2.743490
12	C	3.096135	2.992147	2.006154	3.809071	2.872436

		11	12
11	C	0.000000	
12	C	2.910567	0.000000

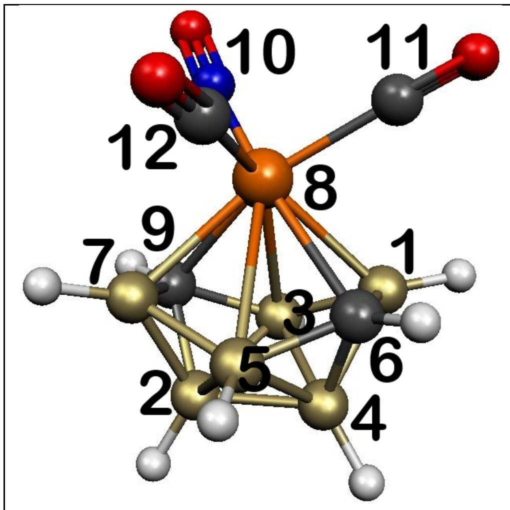

5. -665.2495105 +9.9 (C_2)

		1	2	3	4	5
1	B	0.000000				
2	B	2.900804	0.000000			
3	B	1.747508	1.840978	0.000000		
4	B	1.757072	1.737285	1.840539	0.000000	
5	B	2.705235	1.809371	2.746685	1.813615	0.000000
6	C	1.538615	2.733925	2.591326	1.647616	1.604864
7	B	3.427349	1.765933	2.698575	2.906284	1.764206
8	Re	2.273530	3.073650	2.451864	3.086620	2.601835
9	C	2.950425	1.638192	1.626312	2.757434	2.601485
10	N	3.698533	4.295417	3.426907	4.667867	4.280657
11	C	2.630351	4.775092	3.738144	4.115861	4.003095
12	C	3.921302	4.195758	4.262103	4.364482	3.064508

		6	7	8	9	10
6	C	0.000000				
7	B	2.904249	0.000000			
8	Re	2.277717	2.302565	0.000000		
9	C	3.168627	1.518994	2.218828	0.000000	
10	N	4.088867	3.348686	1.835257	2.852999	0.000000
11	C	2.877482	4.261863	2.027253	4.116432	2.776405
12	C	3.165986	2.778439	1.998078	3.590948	2.860234

		11	12
11	C	0.000000	
12	C	2.883205	0.000000

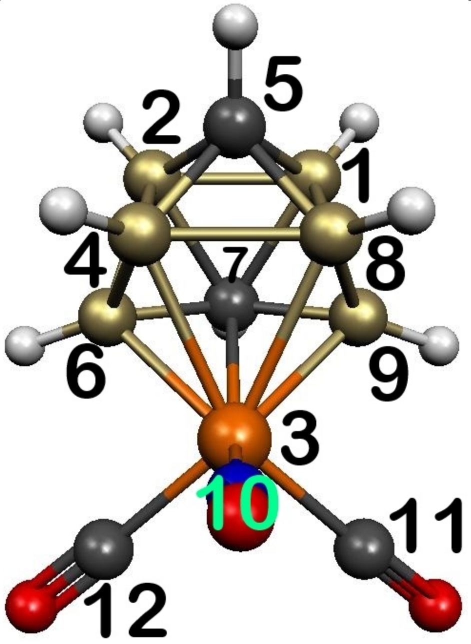

6. -665.2467502 +11.6 C_S

		1	2	3	4	5
1	B	0.000000				
2	B	1.849832	0.000000			
3	Re	3.287030	3.286922	0.000000		
4	B	2.620192	1.776175	2.441403	0.000000	
5	C	1.584541	1.584606	3.361079	1.597922	0.000000
6	B	2.860479	1.691805	2.195841	1.728483	2.699754
7	C	1.819661	1.818606	2.316973	2.531214	2.613346
8	B	1.776514	2.620453	2.442055	2.005961	1.597712
9	B	1.691391	2.859322	2.195457	2.958407	2.699311
10	N	4.544696	4.542908	1.829660	3.074549	4.141146
11	C	4.273261	4.864404	2.003321	4.345539	4.882819
12	C	4.864530	4.276097	2.003546	3.615000	4.886423

		6	7	8	9	10
6	B	0.000000				
7	C	1.642916	0.000000			
8	B	2.959604	2.532665	0.000000		
9	B	2.874600	1.643145	1.729480	0.000000	
10	N	3.573500	4.030213	3.077530	3.577013	0.000000
11	C	3.913462	3.351418	3.609740	2.630070	2.878976
12	C	2.633402	3.349002	4.347981	3.910148	2.878830

		11	12
11	C	0.000000	
12	C	2.913876	0.000000

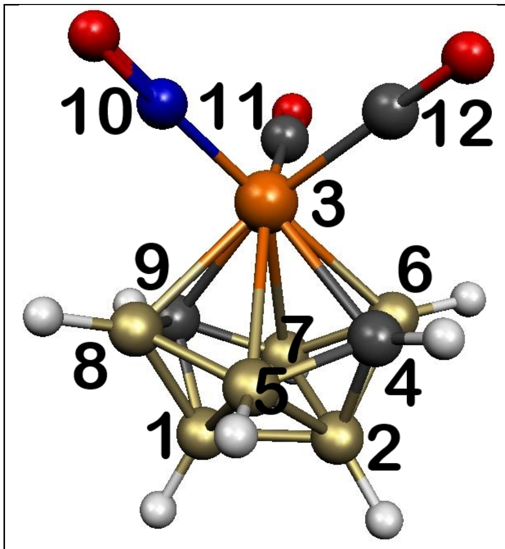

7. -665.2440586 +13.3 (C_2)

		1	2	3	4	5
1	B	0.000000				
2	B	1.734478	0.000000			
3	Re	3.085560	3.101448	0.000000		
4	C	2.748200	1.645179	2.257090	0.000000	
5	B	1.832956	1.834620	2.495408	1.614215	0.000000
6	B	2.896282	1.760864	2.326527	1.525845	2.694106
7	B	1.819928	1.816192	2.570702	2.592384	2.740842
8	B	1.749241	2.903182	2.277119	2.945191	1.761055
9	C	1.652861	2.754069	2.255746	3.176483	2.598988
10	N	4.270048	4.724836	1.845141	3.813125	3.564218
11	C	4.266292	4.283547	1.985652	3.777871	4.296586
12	C	4.782586	4.177094	2.019361	2.714534	3.656875

		6	7	8	9	10
6	B	0.000000				
7	B	1.746950	0.000000			
8	B	3.427822	2.695374	0.000000		
9	C	2.924199	1.624125	1.518136	0.000000	
10	N	4.167628	4.183490	2.775690	3.236133	0.000000
11	C	2.998527	3.001121	3.766645	2.956959	2.856585
12	C	2.974867	4.115008	4.086726	4.240406	2.753798

		11	12
11	C	0.000000	
12	C	2.879871	0.000000

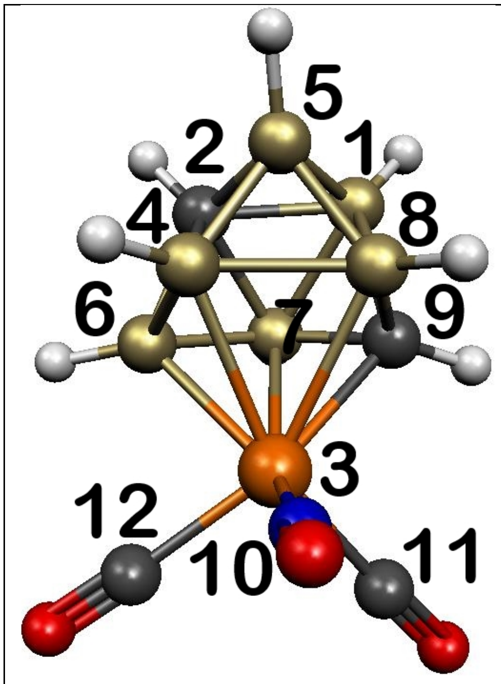

8. -665.2363115 +18.1 C_1

		1	2	3	4	5
1	B	0.000000				
2	C	1.778187	0.000000			
3	Re	3.317060	3.147275	0.000000		
4	B	2.611806	1.690083	2.429827	0.000000	
5	B	1.676874	1.625435	3.521975	1.679185	0.000000
6	B	2.885467	1.612903	2.193252	1.729137	2.828746
7	B	1.884131	1.809784	2.361971	2.638030	2.860708
8	B	1.776669	2.491764	2.476084	1.977840	1.692329
9	C	1.606726	2.600542	2.170326	2.793971	2.696303
10	N	4.530010	4.440329	1.841953	3.110601	4.256293
11	C	4.397824	4.742790	2.006121	4.368524	5.137118
12	C	4.921102	4.161345	1.985587	3.514628	4.980740

		6	7	8	9	10
6	B	0.000000				
7	B	1.747391	0.000000			
8	B	2.970054	2.615043	0.000000		
9	C	2.791764	1.597950	1.612146	0.000000	
10	N	3.635797	4.092784	3.045466	3.470265	0.000000
11	C	3.869505	3.321098	3.781611	2.834100	2.875965
12	C	2.619592	3.466698	4.335633	3.961782	2.838549

		11	12
11	C	0.000000	
12	C	2.868874	0.000000

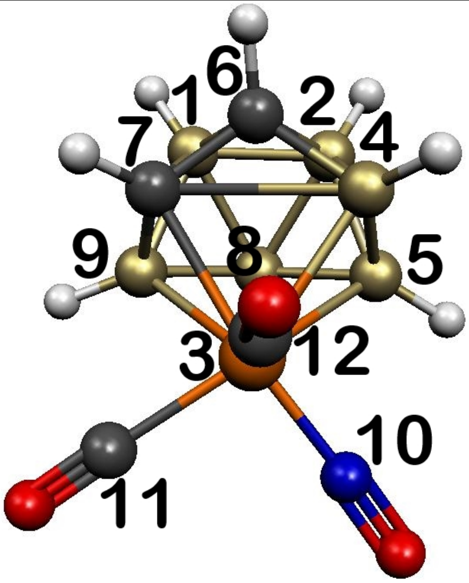

9. -665.2236182 +26.1 (C_2)

		1	2	3	4	5
1	B	0.000000				
2	B	1.779959	0.000000			
3	Re	3.205965	3.173981	0.000000		
4	B	2.798004	1.756702	2.401010	0.000000	
5	B	2.888237	1.692770	2.263847	1.659980	0.000000
6	C	1.666241	1.683038	2.910645	1.616873	2.652455
7	C	1.664929	2.659243	2.341357	2.542793	3.143276
8	B	1.843295	1.844787	2.449737	2.722317	1.758616
9	B	1.722615	2.869229	2.262305	3.263815	3.017892
10	N	4.755143	4.270411	1.829009	3.434957	2.748272
11	C	4.212346	4.841091	2.027206	4.338522	4.121277
12	C	4.395064	4.302502	1.989186	2.848884	3.633481

		6	7	8	9	10
6	C	0.000000				
7	C	1.518766	0.000000			
8	B	2.668210	2.559848	0.000000		
9	B	2.626241	1.564154	1.718581	0.000000	
10	N	4.474037	4.153334	3.444199	3.805347	0.000000
11	C	4.374367	3.161716	3.615375	2.618418	2.853042
12	C	3.406173	3.018306	4.231472	3.761142	2.889708

		11	12
11	C	0.000000	
12	C	2.961786	0.000000

Table 2C. Energy ranking for $(CO)_2(NO)ReC_2B_6H_8$ obtained after B3LYP/6-31G(d)//SDD optimization:

No	Initial structure	Final energy (a.u.)	ΔE (kcal/mol)
1	01-CapCube---ReC2---7	-665.0680266	0.00
2	02-PressCapCube---ReC2---104	-665.0680076	0.01
3	02-PressCapCube---ReC2---81	-665.0680049	0.01
4	02-PressCapCube---ReC2---117	-665.0679898	0.02
5	03-Tl99---ReC2---181	-665.0679732	0.03
6	04-TrTrPr---ReC2---232	-665.0667938	0.77
7	04-TrTrPr---ReC2---246	-665.0667938	0.77
8	01-CapCube---ReC2---11	-665.0667851	0.78
9	02-PressCapCube---ReC2---103	-665.0667670	0.79
10	01-CapCube---ReC2---49	-665.0667654	0.79
11	01-CapCube---ReC2---20	-665.0667622	0.79
12	02-PressCapCube---ReC2---86	-665.0667113	0.83
13	02-PressCapCube---ReC2---95	-665.0624525	3.50
14	04-TrTrPr---ReC2---247	-665.0616402	4.01
15	02-PressCapCube---ReC2---82	-665.0616040	4.03
16	02-PressCapCube---ReC2---98	-665.0616040	4.03
17	03-Tl99---ReC2---195	-665.0558072	7.67
18	03-Tl99---ReC2---174	-665.0537095	8.98
19	03-Tl99---ReC2---196	-665.0537072	8.99
20	01-CapCube---ReC2---10	-665.0536961	8.99
21	04-TrTrPr---ReC2---233	-665.0536862	9.00
22	01-CapCube---ReC2---21	-665.0525907	9.69
23	03-Tl99---ReC2---224	-665.0490144	11.93
24	04-TrTrPr---ReC2---245	-665.0489678	11.96
25	04-TrTrPr---ReC2---259	-665.0489678	11.96
26	02-PressCapCube---ReC2---88	-665.0481517	12.47

27	02-PressCapCube---ReC2---80	-665.0439853	15.09
28	02-PressCapCube---ReC2---79	-665.0351045	20.66
29	02-PressCapCube---ReC2---89	-665.0288120	24.61
30	02-PressCapCube---ReC2---96	-665.0288120	24.61
31	03-Tl99---ReC2---172	-665.0286116	24.73
32	04-TrTrPr---ReC2---237	-665.0227972	28.38
33	04-TrTrPr---ReC2---240	-665.0227972	28.38
34	02-PressCapCube---ReC2---101	-665.0224139	28.62
35	01-CapCube---ReC2---6	-665.0213111	29.31
36	03-Tl99---ReC2---188	-665.0212431	29.36
37	04-TrTrPr---ReC2---243	-665.0212367	29.36
38	03-Tl99---ReC2---173	-665.0204075	29.88
39	03-Tl99---ReC2---203	-665.0204075	29.88
40	03-Tl99---ReC2---128	-665.0204004	29.89
41	03-Tl99---ReC2---134	-665.0204004	29.89
42	01-CapCube---ReC2---5	-665.0194386	30.49
43	04-TrTrPr---ReC2---258	-665.0194370	30.49
44	04-TrTrPr---ReC2---255	-665.0194005	30.51
45	02-PressCapCube---ReC2---120	-665.0183088	31.20
46	02-PressCapCube---ReC2---113	-665.0183078	31.20
47	04-TrTrPr---ReC2---270	-665.0183049	31.20
48	04-TrTrPr---ReC2---277	-665.0183049	31.20
49	03-Tl99---ReC2---226	-665.0183040	31.20
50	02-PressCapCube---ReC2---51	-665.0182956	31.21
51	03-Tl99---ReC2---133	-665.0182639	31.23
52	02-PressCapCube---ReC2---87	-665.0161007	32.58
53	02-PressCapCube---ReC2---102	-665.0156019	32.90
54	02-PressCapCube---ReC2---93	-665.0156009	32.90

55	03-Tl99---ReC2---155	-665.0155346	32.94
56	03-Tl99---ReC2---175	-665.0143568	33.68
57	02-PressCapCube---ReC2---58	-665.0137806	34.04
58	03-Tl99---ReC2---197	-665.0137763	34.04
59	02-PressCapCube---ReC2---77	-665.0137741	34.04
60	01-CapCube---ReC2---17	-665.0136041	34.15
61	01-CapCube---ReC2---28	-665.0136041	34.15
62	02-PressCapCube---ReC2---94	-665.0135811	34.17
63	02-PressCapCube---ReC2---112	-665.0125850	34.79
64	03-Tl99---ReC2---208	-665.0125635	34.80
65	03-Tl99---ReC2---230	-665.0125635	34.80
66	03-Tl99---ReC2---130	-665.0125449	34.82
67	04-TrTrPr---ReC2---269	-665.0120485	35.13
68	01-CapCube---ReC2---4	-665.0114380	35.51
69	01-CapCube---ReC2---8	-665.0114380	35.51
70	02-PressCapCube---ReC2---92	-665.0113383	35.57
71	01-CapCube---ReC2---41	-665.0103923	36.17
72	03-Tl99---ReC2---129	-665.0103843	36.17
73	02-PressCapCube---ReC2---71	-665.0103841	36.17
74	04-TrTrPr---ReC2---266	-665.0103784	36.18
75	04-TrTrPr---ReC2---294	-665.0103784	36.18
76	02-PressCapCube---ReC2---116	-665.0093855	36.80
77	04-TrTrPr---ReC2---276	-665.0087019	37.23
78	03-Tl99---ReC2---213	-665.0086966	37.23
79	03-Tl99---ReC2---222	-665.0086966	37.23
80	01-CapCube---ReC2---34	-665.0086656	37.25
81	01-CapCube---ReC2---13	-665.0086559	37.26
82	04-TrTrPr---ReC2---285	-665.0067331	38.46

83	01-CapCube---ReC2---36	-665.0066877	38.49
84	04-TrTrPr---ReC2---293	-665.0063827	38.68
85	02-PressCapCube---ReC2---109	-665.0063734	38.69
86	02-PressCapCube---ReC2---85	-665.0063728	38.69
87	03-Tl99---ReC2---217	-665.0063643	38.69
88	03-Tl99---ReC2---178	-665.0054212	39.29
89	01-CapCube---ReC2---22	-665.0054129	39.29
90	03-Tl99---ReC2---167	-665.0054107	39.29
91	03-Tl99---ReC2---148	-665.0053861	39.31
92	03-Tl99---ReC2---165	-665.0053861	39.31
93	03-Tl99---ReC2---171	-665.0040887	40.12
94	03-Tl99---ReC2---182	-665.0040887	40.12
95	03-Tl99---ReC2---169	-665.0037024	40.36
96	02-PressCapCube---ReC2---63	-665.0036760	40.38
97	01-CapCube---ReC2---42	-665.0036329	40.41
98	01-CapCube---ReC2---19	-665.0036292	40.41
99	04-TrTrPr---ReC2---281	-665.0007811	42.20
100	01-CapCube---ReC2---3	-664.9986214	43.55
101	04-TrTrPr---ReC2---239	-664.9985804	43.58
102	04-TrTrPr---ReC2---251	-664.9985804	43.58
103	04-TrTrPr---ReC2---242	-664.9985242	43.61
104	02-PressCapCube---ReC2---100	-664.9983748	43.71
105	02-PressCapCube---ReC2---52	-664.9973726	44.34
106	02-PressCapCube---ReC2---62	-664.9973726	44.34
107	02-PressCapCube---ReC2---90	-664.9973554	44.35
108	03-Tl99---ReC2---205	-664.9973399	44.36
109	03-Tl99---ReC2---179	-664.9969568	44.60
110	03-Tl99---ReC2---199	-664.9969568	44.60

111	02-PressCapCube---ReC2---110	-664.9969397	44.61
112	02-PressCapCube---ReC2---97	-664.9969366	44.61
113	03-Tl99---ReC2---207	-664.9969258	44.62
114	03-Tl99---ReC2---127	-664.9969256	44.62
115	03-Tl99---ReC2---141	-664.9969108	44.63
116	03-Tl99---ReC2---166	-664.9969108	44.63
117	01-CapCube---ReC2---27	-664.9968684	44.65
118	02-PressCapCube---ReC2---108	-664.9968431	44.67
119	04-TrTrPr---ReC2---260	-664.9968287	44.68
120	02-PressCapCube---ReC2---114	-664.9968187	44.68
121	02-PressCapCube---ReC2---57	-664.9967110	44.75
122	01-CapCube---ReC2---2	-664.9966109	44.81
123	03-Tl99---ReC2---153	-664.9965817	44.83
124	02-PressCapCube---ReC2---118	-664.9965569	44.85
125	04-TrTrPr---ReC2---265	-664.9964919	44.89
126	04-TrTrPr---ReC2---256	-664.9956995	45.39
127	04-TrTrPr---ReC2---252	-664.9948409	45.93
128	04-TrTrPr---ReC2---253	-664.9948409	45.93
129	01-CapCube---ReC2---12	-664.9948405	45.93
130	03-Tl99---ReC2---161	-664.9944987	46.14
131	03-Tl99---ReC2---206	-664.9942589	46.29
132	03-Tl99---ReC2---223	-664.9942589	46.29
133	02-PressCapCube---ReC2---50	-664.9940077	46.45
134	02-PressCapCube---ReC2---68	-664.9938108	46.57
135	03-Tl99---ReC2---136	-664.9932149	46.95
136	04-TrTrPr---ReC2---241	-664.9931530	46.98
137	02-PressCapCube---ReC2---76	-664.9931252	47.00
138	02-PressCapCube---ReC2---53	-664.9931202	47.01

139	02-PressCapCube---ReC2---56	-664.9925949	47.33
140	04-TrTrPr---ReC2---238	-664.9922798	47.53
141	04-TrTrPr---ReC2---254	-664.9922798	47.53
142	03-Tl99---ReC2---170	-664.9911796	48.22
143	03-Tl99---ReC2---189	-664.9911796	48.22
144	04-TrTrPr---ReC2---257	-664.9911469	48.24
145	04-TrTrPr---ReC2---244	-664.9911244	48.26
146	03-Tl99---ReC2---231	-664.9884655	49.93
147	03-Tl99---ReC2---187	-664.9877582	50.37
148	03-Tl99---ReC2---191	-664.9877582	50.37
149	03-Tl99---ReC2---211	-664.9872022	50.72
150	03-Tl99---ReC2---212	-664.9871986	50.72
151	02-PressCapCube---ReC2---119	-664.9868223	50.96
152	04-TrTrPr---ReC2---272	-664.9868089	50.97
153	04-TrTrPr---ReC2---292	-664.9868089	50.97
154	04-TrTrPr---ReC2---250	-664.9861440	51.38
155	04-TrTrPr---ReC2---249	-664.9861039	51.41
156	04-TrTrPr---ReC2---234	-664.9853883	51.86
157	03-Tl99---ReC2---131	-664.9840638	52.69
158	03-Tl99---ReC2---221	-664.9840282	52.71
159	03-Tl99---ReC2---227	-664.9840282	52.71
160	03-Tl99---ReC2---183	-664.9840096	52.72
161	03-Tl99---ReC2---138	-664.9839875	52.74
162	03-Tl99---ReC2---202	-664.9825746	53.62
163	03-Tl99---ReC2---220	-664.9825415	53.64
164	04-TrTrPr---ReC2---282	-664.9821329	53.90
165	01-CapCube---ReC2---32	-664.9821235	53.91
166	01-CapCube---ReC2---37	-664.9821235	53.91

167	03-Tl99---ReC2---156	-664.9818083	54.10
168	04-TrTrPr---ReC2---236	-664.9800771	55.19
169	04-TrTrPr---ReC2---261	-664.9790318	55.85
170	04-TrTrPr---ReC2---287	-664.9790318	55.85
171	03-Tl99---ReC2---150	-664.9790149	55.86
172	01-CapCube---ReC2---39	-664.9790057	55.86
173	04-TrTrPr---ReC2---262	-664.9787158	56.04
174	03-Tl99---ReC2---149	-664.9751860	58.26
175	04-TrTrPr---ReC2---286	-664.9749646	58.40
176	04-TrTrPr---ReC2---289	-664.9749646	58.40
177	02-PressCapCube---ReC2---70	-664.9749415	58.41
178	01-CapCube---ReC2---38	-664.9749284	58.42
179	01-CapCube---ReC2---25	-664.9749282	58.42
180	03-Tl99---ReC2---132	-664.9749222	58.42
181	04-TrTrPr---ReC2---278	-664.9748968	58.44
182	02-PressCapCube---ReC2---123	-664.9748942	58.44
183	01-CapCube---ReC2---35	-664.9744678	58.71
184	01-CapCube---ReC2---16	-664.9744667	58.71
185	03-Tl99---ReC2---162	-664.9737164	59.18
186	03-Tl99---ReC2---163	-664.9737033	59.19
187	01-CapCube---ReC2---43	-664.9731632	59.53
188	03-Tl99---ReC2---137	-664.9731249	59.55
189	01-CapCube---ReC2---9	-664.9716929	60.45
190	03-Tl99---ReC2---194	-664.9712155	60.75
191	03-Tl99---ReC2---201	-664.9712155	60.75
192	01-CapCube---ReC2---31	-664.9704469	61.23
193	03-Tl99---ReC2---219	-664.9701306	61.43
194	03-Tl99---ReC2---180	-664.9693740	61.91

195	03-Tl99---ReC2---192	-664.9693740	61.91
196	03-Tl99---ReC2---135	-664.9692199	62.00
197	03-Tl99---ReC2---186	-664.9692040	62.01
198	03-Tl99---ReC2---198	-664.9692040	62.01
199	03-Tl99---ReC2---164	-664.9688035	62.26
200	03-Tl99---ReC2---200	-664.9687900	62.27
201	03-Tl99---ReC2---214	-664.9687699	62.29
202	04-TrTrPr---ReC2---248	-664.9687518	62.30
203	03-Tl99---ReC2---215	-664.9687211	62.32
204	03-Tl99---ReC2---229	-664.9687211	62.32
205	02-PressCapCube---ReC2---126	-664.9687173	62.32
206	01-CapCube---ReC2---26	-664.9682792	62.59
207	01-CapCube---ReC2---45	-664.9682774	62.59
208	03-Tl99---ReC2---218	-664.9678954	62.83
209	02-PressCapCube---ReC2---111	-664.9678697	62.85
210	02-PressCapCube---ReC2---54	-664.9664330	63.75
211	03-Tl99---ReC2---228	-664.9657118	64.20
212	04-TrTrPr---ReC2---288	-664.9648422	64.75
213	02-PressCapCube---ReC2---78	-664.9643901	65.03
214	01-CapCube---ReC2---48	-664.9643566	65.05
215	03-Tl99---ReC2---190	-664.9638411	65.38
216	04-TrTrPr---ReC2---267	-664.9636503	65.50
217	03-Tl99---ReC2---204	-664.9635943	65.53
218	01-CapCube---ReC2---47	-664.9635749	65.55
219	01-CapCube---ReC2---40	-664.9635739	65.55
220	03-Tl99---ReC2---154	-664.9632473	65.75
221	03-Tl99---ReC2---160	-664.9631942	65.78
222	04-TrTrPr---ReC2---268	-664.9625202	66.21

223	04-TrTrPr---ReC2---284	-664.9625202	66.21
224	04-TrTrPr---ReC2---279	-664.9616718	66.74
225	04-TrTrPr---ReC2---291	-664.9616718	66.74
226	03-Tl99---ReC2---142	-664.9610963	67.10
227	03-Tl99---ReC2---143	-664.9610604	67.12
228	01-CapCube---ReC2---1	-664.9608622	67.25
229	03-Tl99---ReC2---176	-664.9607650	67.31
230	03-Tl99---ReC2---210	-664.9607547	67.32
231	04-TrTrPr---ReC2---235	-664.9606411	67.39
232	02-PressCapCube---ReC2---64	-664.9603647	67.56
233	02-PressCapCube---ReC2---60	-664.9601507	67.69
234	03-Tl99---ReC2---140	-664.9598849	67.86
235	03-Tl99---ReC2---168	-664.9595997	68.04
236	03-Tl99---ReC2---139	-664.9595007	68.10
237	04-TrTrPr---ReC2---290	-664.9591224	68.34
238	02-PressCapCube---ReC2---121	-664.9586877	68.61
239	02-PressCapCube---ReC2---105	-664.9585236	68.72
240	04-TrTrPr---ReC2---274	-664.9568149	69.79
241	01-CapCube---ReC2---15	-664.9560105	70.29
242	03-Tl99---ReC2---209	-664.9552375	70.78
243	03-Tl99---ReC2---216	-664.9552375	70.78
244	02-PressCapCube---ReC2---106	-664.9551882	70.81
245	02-PressCapCube---ReC2---122	-664.9551882	70.81
246	04-TrTrPr---ReC2---264	-664.9551531	70.83
247	04-TrTrPr---ReC2---273	-664.9551531	70.83
248	02-PressCapCube---ReC2---74	-664.9538183	71.67
249	02-PressCapCube---ReC2---107	-664.9526285	72.41
250	02-PressCapCube---ReC2---115	-664.9526285	72.41

251	02-PressCapCube---ReC2---84	-664.9526127	72.42
252	03-Tl99---ReC2---225	-664.9514217	73.17
253	04-TrTrPr---ReC2---263	-664.9514145	73.18
254	04-TrTrPr---ReC2---280	-664.9514145	73.18
255	01-CapCube---ReC2---18	-664.9503735	73.83
256	01-CapCube---ReC2---29	-664.9493813	74.45
257	02-PressCapCube---ReC2---67	-664.9493143	74.49
258	02-PressCapCube---ReC2---75	-664.9493143	74.49
259	04-TrTrPr---ReC2---271	-664.9492063	74.56
260	03-Tl99---ReC2---185	-664.9482906	75.14
261	02-PressCapCube---ReC2---83	-664.9479302	75.36
262	02-PressCapCube---ReC2---91	-664.9479302	75.36
263	01-CapCube---ReC2---46	-664.9456517	76.79
264	04-TrTrPr---ReC2---275_i-33	-664.9450656	77.16
265	04-TrTrPr---ReC2---283_i-33	-664.9450656	77.16
266	04-TrTrPr---ReC2---275_r-33	-664.9449769	77.22
267	04-TrTrPr---ReC2---283_r-33	-664.9449769	77.22
268	03-Tl99---ReC2---177	-664.9445507	77.48
269	03-Tl99---ReC2---184	-664.9445507	77.48
270	01-CapCube---ReC2---33	-664.9431800	78.34
271	01-CapCube---ReC2---44	-664.9431800	78.34
272	03-Tl99---ReC2---193	-664.9427271	78.63
273	03-Tl99---ReC2---144	-664.9396421	80.56
274	03-Tl99---ReC2---159	-664.9396421	80.56
275	01-CapCube---ReC2---14	-664.9395974	80.59
276	03-Tl99---ReC2---147	-664.9395711	80.61
277	02-PressCapCube---ReC2---65_r-21	-664.9391580	80.87
278	02-PressCapCube---ReC2---99	-664.9390747	80.92

279	02-PressCapCube---ReC2---65_i-21	-664.9390658	80.93
280	02-PressCapCube---ReC2---125	-664.9370515	82.19
281	02-PressCapCube---ReC2---61	-664.9348241	83.59
282	02-PressCapCube---ReC2---66	-664.9348241	83.59
283	02-PressCapCube---ReC2---124	-664.9343333	83.90
284	02-PressCapCube---ReC2---69	-664.9340910	84.05
285	02-PressCapCube---ReC2---55	-664.9340829	84.05
286	03-Tl99---ReC2---146	-664.9336043	84.35
287	01-CapCube---ReC2---24	-664.9335980	84.36
288	03-Tl99---ReC2---145	-664.9335729	84.37
289	03-Tl99---ReC2---152	-664.9335729	84.37
290	01-CapCube---ReC2---23	-664.9300583	86.58
291	01-CapCube---ReC2---30	-664.9300583	86.58
292	02-PressCapCube---ReC2---72	-664.9294673	86.95
293	03-Tl99---ReC2---151	-664.9280019	87.87
294	03-Tl99---ReC2---158	-664.9280019	87.87
295	03-Tl99---ReC2---157	-664.9250228	89.74
296	02-PressCapCube---ReC2---59	-664.9117183	98.09
297	02-PressCapCube---ReC2---73	-664.9117183	98.09

Table 3A. Initial $(CO)_2(NO)ReC_2B_7H_9$ structures (one example from each family), a total of 384 structures:

**Initial structures**	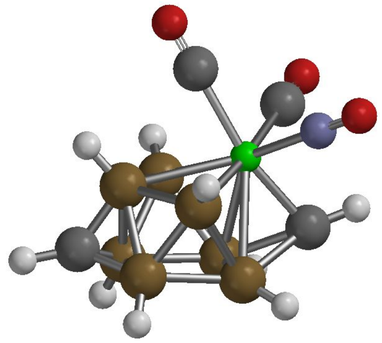 1. Tetracapped trigonal prism (C_{2v}) 60 structures
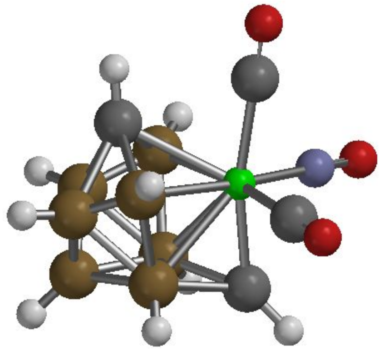 2. Tetracapped trigonal prism (C_{3v}) 36 structures	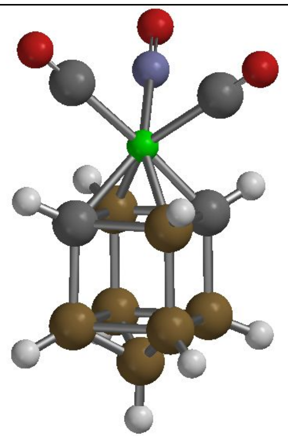 3. Bicapped cube 80 structures
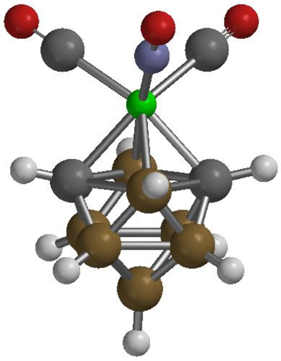 4. Bicapped square antiprism 96 structures	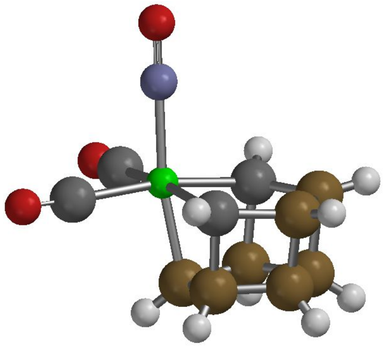 5. Pentagonal prism 8
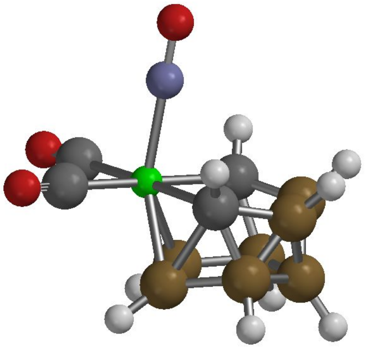	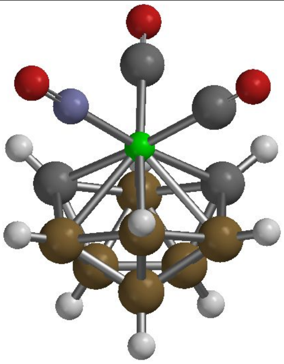

6. Pentagonal antiprism 8	7. Isocloso structure 96 structures

Table 3B. Distances table for the lowest-lying $(CO)_2(NO)ReC_2B_7H_9$ structures after M06L/6-311G(d,p)//SDD optimization. Included are the ZPcorrected E (a.u.), relative energy (kcal/mol) and symmetry:

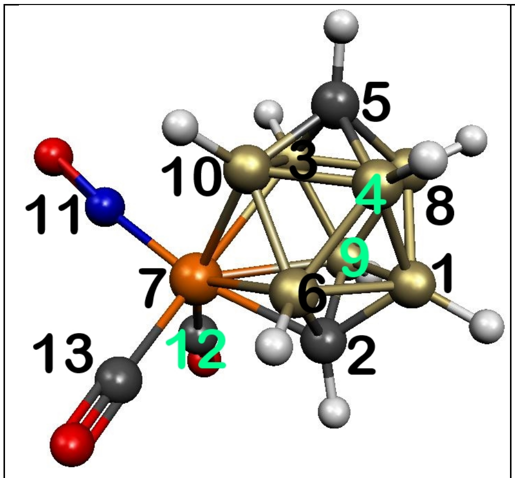

1. -690.75877310 0.0 C_S

		1	2	3	4	5
1	B	0.000000				
2	C	1.580629	0.000000			
3	B	2.821373	2.855417	0.000000		
4	B	1.773228	2.771699	2.586321	0.000000	
5	C	2.734593	3.420458	1.590471	1.585752	0.000000
6	B	1.837128	1.618751	2.851894	1.810770	2.764301
7	Re	3.291099	2.154031	2.351429	3.538536	3.406904
8	B	1.774190	2.772037	1.807941	1.811518	1.585780
9	B	1.838265	1.619678	1.807321	2.820214	2.764977
10	B	2.822444	2.857276	1.888386	1.808568	1.589672
11	N	4.894585	3.970934	2.976851	4.659127	4.018784
12	C	4.364294	2.999961	3.569430	5.171416	4.976949
13	C	4.368315	3.012275	4.248897	4.637883	4.964454
		6	7	8	9	10
6	B	0.000000				
7	Re	2.370851	0.000000			
8	B	2.819813	3.538560	0.000000		
9	B	2.579838	2.371790	1.810861	0.000000	
10	B	1.808533	2.352951	2.586928	2.854358	0.000000
11	N	3.832053	1.841605	4.653436	3.823190	2.986351
12	C	4.009381	1.988667	4.648125	2.959914	4.255556
13	C	2.952435	1.988933	5.170409	4.019853	3.552547
		11	12	13		
11	N	0.000000				
12	C	2.846261	0.000000			
13	C	2.846516	2.860102	0.000000		

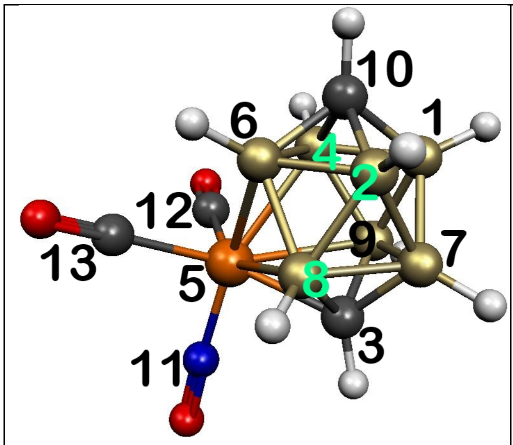

2. -690.75555840 +2.0 C_S

		1	2	3	4	5
1	B	0.000000				
2	B	1.817468	0.000000			
3	C	2.761138	2.762701	0.000000		
4	B	1.811534	2.574131	2.845486	0.000000	
5	Re	3.545775	3.547147	2.132084	2.365639	0.000000
6	B	2.574232	1.809699	2.846532	1.842166	2.368863
7	B	1.769295	1.770508	1.581521	2.820409	3.290112
8	B	2.819288	1.802702	1.614730	2.847719	2.384006
9	B	1.804486	2.822005	1.612692	1.830903	2.380919
10	C	1.585262	1.585636	3.421431	1.590942	3.446384
11	N	5.071521	5.056584	2.829151	4.114496	1.843442
12	C	4.392132	4.953473	3.782161	2.765543	1.987125
13	C	4.937756	4.400643	3.814142	3.558154	1.989079

		6	7	8	9	10
6	B	0.000000				
7	B	2.820090	0.000000			
8	B	1.829090	1.832029	0.000000		
9	B	2.849962	1.833330	2.587719	0.000000	
10	C	1.591718	2.733658	2.778572	2.779827	0.000000
11	N	4.096477	4.374535	3.512456	3.542371	5.228482
12	C	3.602253	4.759819	4.242224	3.243808	4.241081
13	C	2.754523	4.780126	3.285161	4.234251	4.213440

		11	12	13
11	N	0.000000		
12	C	2.814605	0.000000	
13	C	2.806479	2.824173	0.000000

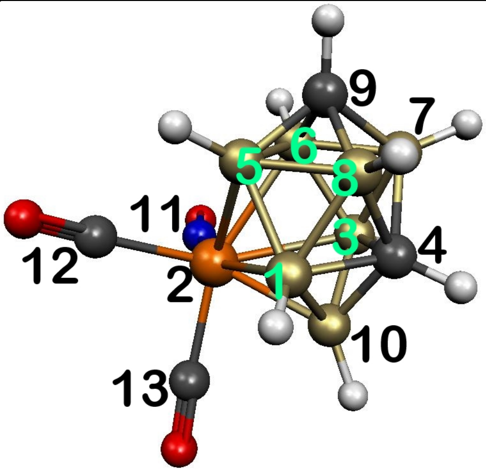

3. -690.74107820 +11.1 (C_S)

		1	2	3	4	5
1	B	0.000000				
2	Re	2.383522	0.000000			
3	B	2.594325	2.365801	0.000000		
4	C	1.721289	3.118289	1.725598	0.000000	
5	B	1.806586	2.349798	2.842950	2.669635	0.000000
6	B	2.859702	2.365307	1.811972	2.675154	1.872188
7	B	2.803810	3.506757	1.759802	1.690374	2.584260
8	B	1.767588	3.518054	2.803201	1.690091	1.808096
9	C	2.751074	3.422269	2.750436	2.603545	1.602637
10	B	1.744790	2.188115	1.734110	1.611781	3.000978
11	N	4.153352	1.837991	3.094625	4.475156	3.737756
12	C	3.327096	2.006456	4.234192	4.645086	2.747059
13	C	3.263981	1.990204	3.632652	4.101095	4.026783

		6	7	8	9	10
6	B	0.000000				
7	B	1.792450	0.000000			
8	B	2.580114	1.830552	0.000000		
9	C	1.590303	1.587129	1.580369	0.000000	
10	B	3.013044	2.892064	2.901597	3.581833	0.000000
11	N	2.933048	4.400683	4.980144	4.418014	3.569503
12	C	3.556546	4.924309	4.407032	4.201811	3.914390
13	C	4.273743	5.054431	4.878707	5.222429	2.590037

		11	12	13
11	N	0.000000		
12	C	2.877581	0.000000	
13	C	2.880775	2.885229	0.000000

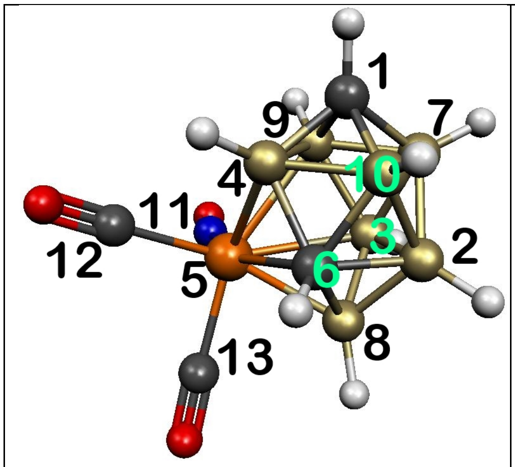

4. -690.73514420 +14.8 C_1

		1	2	3	4	5
1	C	0.000000				
2	B	2.712319	0.000000			
3	B	2.775285	1.819388	0.000000		
4	B	1.592708	2.775682	2.835387	0.000000	
5	Re	3.404838	3.276575	2.401485	2.310331	0.000000
6	C	2.641257	1.731324	2.481817	1.711318	2.339873
7	B	1.597504	1.759423	1.793613	2.576290	3.536002
8	B	3.557119	1.679218	1.726338	2.930967	2.189549
9	B	1.590854	2.796645	1.819584	1.872450	2.363428
10	B	1.579178	1.741421	2.810259	1.821358	3.538815
11	N	4.423290	4.613607	3.118682	3.723998	1.824163
12	C	4.170899	4.825913	4.270791	2.746293	2.006580
13	C	5.213110	4.234210	3.675262	3.980813	1.996249

		6	7	8	9	10
6	C	0.000000				
7	B	2.688816	0.000000			
8	B	1.661669	2.902006	0.000000		
9	B	2.735259	1.793545	2.981519	0.000000	
10	B	1.720531	1.804028	2.887874	2.578847	0.000000
11	N	4.077889	4.438601	3.550047	2.953067	4.998559
12	C	3.382810	4.944192	3.937297	3.549763	4.455863
13	C	3.282846	5.101601	2.625259	4.278922	4.906508

		11	12	13
11	N	0.000000		
12	C	2.844103	0.000000	
13	C	2.851285	2.893969	0.000000

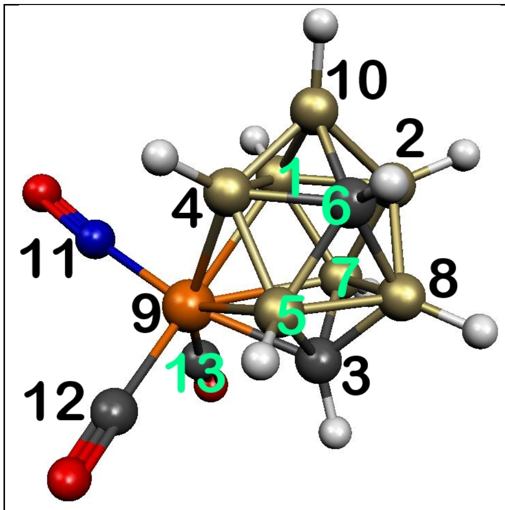

5. -690.73102860 +17.4 C_1

	1	2	3	4	5
1 B	0.000000				
2 B	1.803692	0.000000			
3 C	2.876098	2.756147	0.000000		
4 B	1.862860	2.565347	2.843394	0.000000	
5 B	2.849437	2.803752	1.609564	1.798020	0.000000
6 C	2.446100	1.716444	2.642121	1.711062	1.723571
7 B	1.828054	1.791398	1.621140	2.831941	2.567499
8 B	2.819999	1.747862	1.580099	2.804536	1.841207
9 Re	2.365886	3.525580	2.160435	2.334304	2.355011
10 B	1.685025	1.682254	3.579907	1.686705	2.899795
11 N	2.981983	4.641991	3.974094	2.957883	3.804058
12 C	4.265789	5.160384	3.011541	3.562724	2.955152
13 C	3.564609	4.622733	3.024695	4.229996	4.009702

	6	7	8	9	10
7 B	2.667684	0.000000			
8 B	1.684378	1.822557	0.000000		
9 Re	3.390082	2.377966	3.288193	0.000000	
10 B	1.620634	2.907145	2.856305	3.547019	0.000000
11 N	4.513494	3.834142	4.883732	1.839595	4.100267
12 C	4.536339	4.018161	4.373817	1.986452	5.114214
13 C	5.014886	2.959279	4.369504	1.992348	5.102677

	11	12	13
11 N	0.000000		
12 C	2.840871	0.000000	
13 C	2.846186	2.869385	0.000000

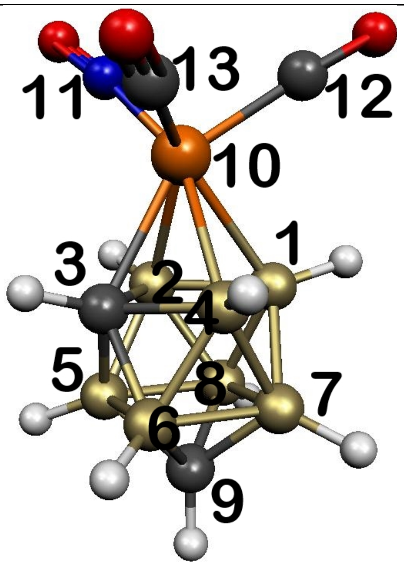

6. -690.72717660 +19.8 (C_S)

		1	2	3	4	5
1	B	0.000000				
2	B	1.812042	0.000000			
3	C	2.477390	1.717003	0.000000		
4	B	1.808578	2.506438	1.715087	0.000000	
5	B	2.820530	1.763263	1.685601	2.776886	0.000000
6	B	2.821493	2.774940	1.681101	1.767157	1.831181
7	B	1.793564	2.776399	2.665418	1.775038	2.577381
8	B	1.798062	1.774737	2.666443	2.777945	1.816501
9	C	2.759498	2.719881	2.599362	2.718444	1.590654
10	Re	2.278138	2.255311	2.291356	2.266147	3.594354
11	N	3.761742	3.027381	3.408778	4.007378	4.483712
12	C	2.914813	3.689822	4.115183	3.450296	5.212543
13	C	3.878133	4.019107	3.248390	2.971771	4.898732

		6	7	8	9	10
6	B	0.000000				
7	B	1.822757	0.000000			
8	B	2.577938	1.820296	0.000000		
9	C	1.588613	1.592310	1.593973	0.000000	
10	Re	3.599773	3.604685	3.600715	4.294722	0.000000
11	N	5.013866	5.219860	4.716733	5.585947	1.837080
12	C	5.093308	4.469361	4.607610	5.565343	1.965044
13	C	4.317727	4.710876	5.245565	5.501935	1.983414

		11	12	13
11	N	0.000000		
12	C	2.806147	0.000000	
13	C	2.867304	2.839522	0.000000

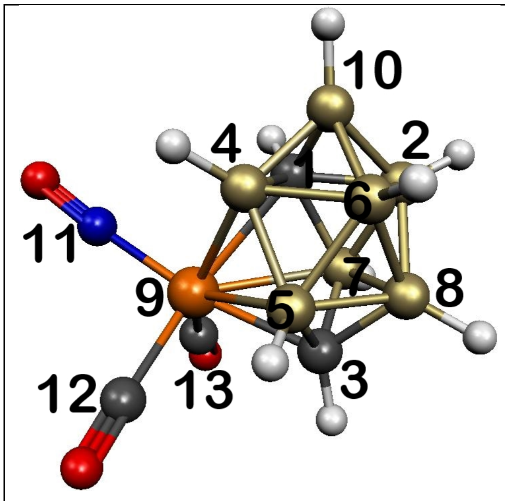

7. -690.72650270 +20.3 C_1

		1	2	3	4	5
1	C	0.000000				
2	B	1.702968	0.000000			
3	C	2.729363	2.761103	0.000000		
4	B	1.750850	2.548685	2.795700	0.000000	
5	B	2.728303	2.815429	1.612936	1.798406	0.000000
6	B	2.446767	1.809762	2.785308	1.804424	1.824051
7	B	1.700987	1.780159	1.609685	2.761400	2.559656
8	B	2.675697	1.760532	1.593305	2.778231	1.827402
9	Re	2.353613	3.564993	2.156983	2.304987	2.381451
10	B	1.618210	1.683571	3.577919	1.695295	2.925355
11	N	3.139974	4.742301	3.977060	2.962383	3.817459
12	C	4.243706	5.203114	2.988978	3.633261	3.035292
13	C	3.509778	4.607707	3.041566	4.163404	4.045497

		6	7	8	9	10
6	B	0.000000				
7	B	2.798343	0.000000			
8	B	1.778476	1.826644	0.000000		
9	Re	3.558019	2.399878	3.314878	0.000000	
10	B	1.682657	2.872804	2.871790	3.562462	0.000000
11	N	4.678090	3.907347	4.932857	1.842209	4.166313
12	C	4.732620	4.001769	4.394198	1.970620	5.190445
13	C	5.170589	2.955924	4.392709	1.988569	5.051100

		11	12	13
11	N	0.000000		
12	C	2.787485	0.000000	
13	C	2.842043	2.825747	0.000000

Table 3C. Energy ranking for $(CO)_2(NO)ReC_2B_7H_9$ obtained after B3LYP/6-31G(d)//SDD optimization:

No	Initial structure	Final energy (a.u.)	ΔE (kcal/mol)
1	01-TetrcTriPri1----ReC2----66	-690.54353730	0.00
2	02-TetrcTriPri2----ReC2----227	-690.54352780	0.01
3	03-BicapCub----ReC2----366	-690.54351350	0.01
4	07-Isocloso----ReC2----524	-690.54351010	0.02
5	01-TetrcTriPri1----ReC2----51	-690.54350690	0.02
6	02-TetrcTriPri2----ReC2----186	-690.54350470	0.02
7	01-TetrcTriPri1----ReC2----36	-690.54350160	0.02
8	01-TetrcTriPri1----ReC2----11	-690.54349860	0.02
9	01-TetrcTriPri1----ReC2----79	-690.54049350	1.91
10	02-TetrcTriPri2----ReC2----278	-690.54047130	1.92
11	07-Isocloso----ReC2----499	-690.54045670	1.93
12	07-Isocloso----ReC2----494	-690.54045630	1.93
13	04-BicapSqAntipr----ReC2----432	-690.54044520	1.94
14	04-BicapSqAntipr----ReC2----472	-690.54044520	1.94
15	02-TetrcTriPri2----ReC2----266	-690.54043980	1.94
16	02-TetrcTriPri2----ReC2----198	-690.54043700	1.95
17	03-BicapCub----ReC2----328	-690.54043260	1.95
18	03-BicapCub----ReC2----352	-690.54043260	1.95
19	01-TetrcTriPri1----ReC2----57	-690.54041060	1.96
20	02-TetrcTriPri2----ReC2----256	-690.53839320	3.23
21	02-TetrcTriPri2----ReC2----291	-690.53839320	3.23
22	03-BicapCub----ReC2----329	-690.53832320	3.27
23	04-BicapSqAntipr----ReC2----467	-690.52046070	14.48
24	04-BicapSqAntipr----ReC2----447	-690.52046060	14.48
25	02-TetrcTriPri2----ReC2----273	-690.52042530	14.50
26	07-Isocloso----ReC2----565	-690.52041160	14.51

27	01-TetrcTriPri1----ReC2----149	-690.51946200	15.11
28	07-Isocloso----ReC2----498	-690.51945350	15.11
29	02-TetrcTriPri2----ReC2----196	-690.51945020	15.12
30	07-Isocloso----ReC2----534	-690.51944900	15.12
31	02-TetrcTriPri2----ReC2----264	-690.51944070	15.12
32	02-TetrcTriPri2----ReC2----294	-690.51944070	15.12
33	07-Isocloso----ReC2----517	-690.51943070	15.13
34	02-TetrcTriPri2----ReC2----232	-690.51942230	15.13
35	02-TetrcTriPri2----ReC2----180	-690.51938080	15.16
36	02-TetrcTriPri2----ReC2----168	-690.51937910	15.16
37	01-TetrcTriPri1----ReC2----111	-690.51937400	15.16
38	07-Isocloso----ReC2----521	-690.51771420	16.20
39	02-TetrcTriPri2----ReC2----202	-690.51766020	16.24
40	02-TetrcTriPri2----ReC2----276	-690.51729880	16.47
41	02-TetrcTriPri2----ReC2----229	-690.51729680	16.47
42	03-BicapCub----ReC2----308	-690.51729280	16.47
43	01-TetrcTriPri1----ReC2----122	-690.51729030	16.47
44	01-TetrcTriPri1----ReC2----153	-690.51729030	16.47
45	02-TetrcTriPri2----ReC2----282	-690.51727730	16.48
46	01-TetrcTriPri1----ReC2----47	-690.51725040	16.50
47	01-TetrcTriPri1----ReC2----74	-690.51724200	16.50
48	01-TetrcTriPri1----ReC2----156	-690.51511580	17.84
49	01-TetrcTriPri1----ReC2----107	-690.51511550	17.84
50	07-Isocloso----ReC2----525	-690.51511380	17.84
51	02-TetrcTriPri2----ReC2----231	-690.51511200	17.84
52	02-TetrcTriPri2----ReC2----162	-690.51510900	17.84
53	02-TetrcTriPri2----ReC2----193	-690.51510630	17.84
54	02-TetrcTriPri2----ReC2----274	-690.51510540	17.84

55	02-TetrcTriPri2----ReC2----250	-690.51510310	17.84
56	02-TetrcTriPri2----ReC2----249	-690.51510010	17.84
57	07-Isocloso----ReC2----503	-690.51509570	17.85
58	05-PentagPrism----ReC2----479	-690.51507330	17.86
59	07-Isocloso----ReC2----527	-690.51507050	17.86
60	01-TetrcTriPri1----ReC2----23	-690.51445870	18.25
61	01-TetrcTriPri1----ReC2----46	-690.51445600	18.25
62	02-TetrcTriPri2----ReC2----295	-690.51444470	18.26
63	02-TetrcTriPri2----ReC2----161	-690.51441000	18.28
64	01-TetrcTriPri1----ReC2----135	-690.51440180	18.28
65	01-TetrcTriPri1----ReC2----150	-690.51440180	18.28
66	04-BicapSqAntipr----ReC2----439	-690.51439960	18.28
67	04-BicapSqAntipr----ReC2----466	-690.51439960	18.28
68	02-TetrcTriPri2----ReC2----272	-690.51439500	18.29
69	02-TetrcTriPri2----ReC2----296	-690.51439190	18.29
70	02-TetrcTriPri2----ReC2----239	-690.51438870	18.29
71	02-TetrcTriPri2----ReC2----195	-690.51431620	18.34
72	02-TetrcTriPri2----ReC2----243	-690.51223010	19.65
73	02-TetrcTriPri2----ReC2----189	-690.51222110	19.65
74	07-Isocloso----ReC2----516	-690.51221120	19.66
75	02-TetrcTriPri2----ReC2----192	-690.51220360	19.66
76	02-TetrcTriPri2----ReC2----184	-690.51220250	19.66
77	02-TetrcTriPri2----ReC2----218	-690.51218760	19.67
78	02-TetrcTriPri2----ReC2----262	-690.51023760	20.90
79	02-TetrcTriPri2----ReC2----263	-690.51020770	20.91
80	02-TetrcTriPri2----ReC2----270	-690.51019880	20.92
81	04-BicapSqAntipr----ReC2----430	-690.51016920	20.94
82	04-BicapSqAntipr----ReC2----456	-690.51016920	20.94

83	04-BicapSqAntipr----ReC2----431	-690.50931750	21.47
84	07-Isocloso----ReC2----553	-690.50930150	21.48
85	07-Isocloso----ReC2----584	-690.50930150	21.48
86	07-Isocloso----ReC2----578	-690.50924620	21.52
87	02-TetrcTriPri2----ReC2----210	-690.50823440	22.15
88	02-TetrcTriPri2----ReC2----235	-690.50821850	22.16
89	01-TetrcTriPri1----ReC2----137	-690.50821710	22.16
90	01-TetrcTriPri1----ReC2----121	-690.50821620	22.16
91	02-TetrcTriPri2----ReC2----191	-690.50819340	22.18
92	01-TetrcTriPri1----ReC2----71	-690.50498220	24.19
93	01-TetrcTriPri1----ReC2----144	-690.50497610	24.20
94	01-TetrcTriPri1----ReC2----90	-690.50497610	24.20
95	04-BicapSqAntipr----ReC2----429	-690.50496620	24.20
96	04-BicapSqAntipr----ReC2----464	-690.50496620	24.20
97	01-TetrcTriPri1----ReC2----59	-690.50494380	24.22
98	07-Isocloso----ReC2----507	-690.50494340	24.22
99	03-BicapCub----ReC2----316	-690.50470060	24.37
100	04-BicapSqAntipr----ReC2----460	-690.50383440	24.91
101	07-Isocloso----ReC2----491	-690.50383370	24.91
102	02-TetrcTriPri2----ReC2----286	-690.50382590	24.92
103	07-Isocloso----ReC2----554	-690.50378380	24.95
104	02-TetrcTriPri2----ReC2----175	-690.50377790	24.95
105	07-Isocloso----ReC2----571	-690.50377750	24.95
106	02-TetrcTriPri2----ReC2----190	-690.50377360	24.95
107	07-Isocloso----ReC2----508	-690.50375350	24.97
108	02-TetrcTriPri2----ReC2----269	-690.50375260	24.97
109	04-BicapSqAntipr----ReC2----428	-690.50374440	24.97
110	03-BicapCub----ReC2----315	-690.50329570	25.25

111	02-TetrcTriPri2----ReC2----252	-690.50040250	27.07
112	02-TetrcTriPri2----ReC2----251	-690.50035920	27.10
113	02-TetrcTriPri2----ReC2----283	-690.50035920	27.10
114	04-BicapSqAntipr----ReC2----455	-690.49329600	31.53
115	04-BicapSqAntipr----ReC2----470	-690.49329520	31.53
116	02-TetrcTriPri2----ReC2----258	-690.49329340	31.53
117	02-TetrcTriPri2----ReC2----257	-690.49326720	31.55
118	02-TetrcTriPri2----ReC2----277	-690.49326720	31.55
119	02-TetrcTriPri2----ReC2----224	-690.49325300	31.55
120	01-TetrcTriPri1----ReC2----84	-690.49324920	31.56
121	01-TetrcTriPri1----ReC2----78	-690.49324900	31.56
122	02-TetrcTriPri2----ReC2----226	-690.49324690	31.56
123	02-TetrcTriPri2----ReC2----200	-690.49324610	31.56
124	07-Isocloso----ReC2----502	-690.49322090	31.57
125	07-Isocloso----ReC2----518	-690.49321980	31.58
126	07-Isocloso----ReC2----532	-690.49320870	31.58
127	04-BicapSqAntipr----ReC2----471	-690.49293280	31.76
128	02-TetrcTriPri2----ReC2----230	-690.49292610	31.76
129	02-TetrcTriPri2----ReC2----248	-690.49292130	31.76
130	07-Isocloso----ReC2----535	-690.49291720	31.77
131	02-TetrcTriPri2----ReC2----280	-690.49291670	31.77
132	02-TetrcTriPri2----ReC2----289	-690.49291670	31.77
133	07-Isocloso----ReC2----522	-690.49291650	31.77
134	01-TetrcTriPri1----ReC2----127	-690.49289870	31.78
135	01-TetrcTriPri1----ReC2----145	-690.49289850	31.78
136	01-TetrcTriPri1----ReC2----27	-690.49288990	31.78
137	02-TetrcTriPri2----ReC2----290	-690.49287770	31.79
138	07-Isocloso----ReC2----566	-690.49255600	31.99

139	07-Isocloso----ReC2----574	-690.49255600	31.99
140	02-TetrcTriPri2----ReC2----167	-690.49252420	32.01
141	02-TetrcTriPri2----ReC2----188	-690.49252420	32.01
142	02-TetrcTriPri2----ReC2----185	-690.49252000	32.01
143	02-TetrcTriPri2----ReC2----199	-690.49252000	32.01
144	02-TetrcTriPri2----ReC2----281	-690.49250750	32.02
145	02-TetrcTriPri2----ReC2----275	-690.49250620	32.02
146	07-Isocloso----ReC2----500	-690.49250010	32.03
147	04-BicapSqAntipr----ReC2----468	-690.49241520	32.08
148	01-TetrcTriPri1----ReC2----151	-690.49100870	32.96
149	01-TetrcTriPri1----ReC2----119	-690.49099490	32.97
150	02-TetrcTriPri2----ReC2----201	-690.49097260	32.99
151	02-TetrcTriPri2----ReC2----194	-690.49096910	32.99
152	02-TetrcTriPri2----ReC2----177_r-50	-690.49094570	33.00
153	07-Isocloso----ReC2----497	-690.49091510	33.02
154	02-TetrcTriPri2----ReC2----247	-690.49080570	33.09
155	02-TetrcTriPri2----ReC2----228	-690.49072720	33.14
156	02-TetrcTriPri2----ReC2----240	-690.49072720	33.14
157	01-TetrcTriPri1----ReC2----130	-690.49069920	33.16
158	01-TetrcTriPri1----ReC2----141	-690.49069920	33.16
159	07-Isocloso----ReC2----495	-690.49069900	33.16
160	07-Isocloso----ReC2----523	-690.49069670	33.16
161	02-TetrcTriPri2----ReC2----170	-690.49069350	33.16
162	07-Isocloso----ReC2----512	-690.49064750	33.19
163	07-Isocloso----ReC2----528	-690.49064440	33.19
164	01-TetrcTriPri1----ReC2----60	-690.49061540	33.21
165	01-TetrcTriPri1----ReC2----80	-690.49061540	33.21
166	02-TetrcTriPri2----ReC2----178	-690.49006640	33.55

167	07-Isocloso----ReC2----519	-690.49005030	33.56
168	07-Isocloso----ReC2----526	-690.49005030	33.56
169	07-Isocloso----ReC2----492	-690.48982860	33.70
170	02-TetrcTriPri2----ReC2----166	-690.48981100	33.71
171	07-Isocloso----ReC2----504	-690.48979070	33.73
172	07-Isocloso----ReC2----511	-690.48979070	33.73
173	01-TetrcTriPri1----ReC2----143	-690.48977710	33.74
174	01-TetrcTriPri1----ReC2----146	-690.48977710	33.74
175	02-TetrcTriPri2----ReC2----169	-690.48972770	33.77
176	02-TetrcTriPri2----ReC2----197	-690.48972770	33.77
177	02-TetrcTriPri2----ReC2----265	-690.48970490	33.78
178	02-TetrcTriPri2----ReC2----279	-690.48970490	33.78
179	04-BicapSqAntipr----ReC2----463	-690.48969560	33.79
180	04-BicapSqAntipr----ReC2----469	-690.48969560	33.79
181	07-Isocloso----ReC2----559	-690.48963640	33.82
182	07-Isocloso----ReC2----568	-690.48963640	33.82
183	01-TetrcTriPri1----ReC2----54	-690.48925550	34.06
184	01-TetrcTriPri1----ReC2----86	-690.48925550	34.06
185	01-TetrcTriPri1----ReC2----82	-690.48922890	34.08
186	02-TetrcTriPri2----ReC2----164	-690.48769250	35.04
187	07-Isocloso----ReC2----496	-690.48769010	35.05
188	01-TetrcTriPri1----ReC2----77	-690.48766180	35.06
189	01-TetrcTriPri1----ReC2----52	-690.48766050	35.06
190	02-TetrcTriPri2----ReC2----174	-690.48765170	35.07
191	02-TetrcTriPri2----ReC2----292	-690.48717880	35.37
192	02-TetrcTriPri2----ReC2----293	-690.48706080	35.44
193	02-TetrcTriPri2----ReC2----288	-690.48702920	35.46
194	02-TetrcTriPri2----ReC2----215	-690.48649560	35.79

195	02-TetrcTriPri2----ReC2----177_i-50	-690.48648390	35.80
196	01-TetrcTriPri1----ReC2----25	-690.48601110	36.10
197	01-TetrcTriPri1----ReC2----8	-690.48601110	36.10
198	01-TetrcTriPri1----ReC2----48	-690.48600850	36.10
199	01-TetrcTriPri1----ReC2----65	-690.48600560	36.10
200	01-TetrcTriPri1----ReC2----152	-690.48599630	36.11
201	01-TetrcTriPri1----ReC2----96	-690.48599220	36.11
202	03-BicapCub----ReC2----355	-690.48595530	36.13
203	03-BicapCub----ReC2----363	-690.48595530	36.13
204	01-TetrcTriPri1----ReC2----87	-690.48586480	36.19
205	01-TetrcTriPri1----ReC2----63	-690.48583510	36.21
206	01-TetrcTriPri1----ReC2----88	-690.48583510	36.21
207	01-TetrcTriPri1----ReC2----2	-690.48582940	36.21
208	01-TetrcTriPri1----ReC2----32	-690.48582650	36.21
209	01-TetrcTriPri1----ReC2----160	-690.48582270	36.22
210	01-TetrcTriPri1----ReC2----91	-690.48582270	36.22
211	06-AntiPr----ReC2----482	-690.48581850	36.22
212	07-Isocloso----ReC2----563	-690.48523800	36.58
213	07-Isocloso----ReC2----557	-690.48517260	36.63
214	04-BicapSqAntipr----ReC2----444	-690.48477590	36.87
215	04-BicapSqAntipr----ReC2----459	-690.48477550	36.87
216	02-TetrcTriPri2----ReC2----261	-690.48468110	36.93
217	02-TetrcTriPri2----ReC2----220	-690.48465260	36.95
218	07-Isocloso----ReC2----546	-690.48464170	36.96
219	02-TetrcTriPri2----ReC2----285	-690.48462860	36.97
220	02-TetrcTriPri2----ReC2----260	-690.48462360	36.97
221	07-Isocloso----ReC2----572	-690.48461060	36.98
222	07-Isocloso----ReC2----549	-690.48460950	36.98

223	01-TetrcTriPri1----ReC2----120	-690.48406970	37.32
224	01-TetrcTriPri1----ReC2----95	-690.48406970	37.32
225	01-TetrcTriPri1----ReC2----1	-690.48406750	37.32
226	01-TetrcTriPri1----ReC2----68	-690.48403510	37.34
227	01-TetrcTriPri1----ReC2----76	-690.48403340	37.34
228	02-TetrcTriPri2----ReC2----204	-690.48343600	37.71
229	04-BicapSqAntipr----ReC2----457	-690.48286770	38.07
230	07-Isocloso----ReC2----505	-690.48274920	38.15
231	07-Isocloso----ReC2----576	-690.48273600	38.15
232	02-TetrcTriPri2----ReC2----287	-690.48273580	38.15
233	02-TetrcTriPri2----ReC2----268	-690.48273190	38.16
234	03-BicapCub----ReC2----321	-690.48270070	38.18
235	02-TetrcTriPri2----ReC2----205	-690.48269240	38.18
236	02-TetrcTriPri2----ReC2----171	-690.48269210	38.18
237	01-TetrcTriPri1----ReC2----114	-690.48269180	38.18
238	01-TetrcTriPri1----ReC2----142	-690.48269180	38.18
239	01-TetrcTriPri1----ReC2----33	-690.48268780	38.18
240	02-TetrcTriPri2----ReC2----206	-690.48268470	38.19
241	07-Isocloso----ReC2----489	-690.48265110	38.21
242	05-PentagPrism----ReC2----474	-690.48261810	38.23
243	05-PentagPrism----ReC2----476	-690.48258740	38.25
244	02-TetrcTriPri2----ReC2----246	-690.48258140	38.25
245	07-Isocloso----ReC2----537	-690.48255290	38.27
246	07-Isocloso----ReC2----583	-690.48255290	38.27
247	01-TetrcTriPri1----ReC2----134	-690.48180090	38.74
248	01-TetrcTriPri1----ReC2----118	-690.48179670	38.74
249	04-BicapSqAntipr----ReC2----438	-690.48179180	38.75
250	01-TetrcTriPri1----ReC2----7	-690.48112020	39.17

251	01-TetrcTriPri1----ReC2----30	-690.48080110	39.37
252	07-Isocloso----ReC2----531	-690.48079790	39.37
253	02-TetrcTriPri2----ReC2----238	-690.48050820	39.55
254	02-TetrcTriPri2----ReC2----165	-690.48048010	39.57
255	02-TetrcTriPri2----ReC2----172	-690.48046770	39.58
256	07-Isocloso----ReC2----509	-690.48042840	39.60
257	06-AntiPr----ReC2----488_i-10	-690.48035070	39.65
258	02-TetrcTriPri2----ReC2----216	-690.48033550	39.66
259	02-TetrcTriPri2----ReC2----225	-690.48033510	39.66
260	02-TetrcTriPri2----ReC2----259	-690.48033130	39.66
261	02-TetrcTriPri2----ReC2----253	-690.48033040	39.66
262	02-TetrcTriPri2----ReC2----211	-690.48032460	39.67
263	02-TetrcTriPri2----ReC2----233	-690.48031950	39.67
264	06-AntiPr----ReC2----488_r-10	-690.48030780	39.68
265	04-BicapSqAntipr----ReC2----427	-690.48030020	39.68
266	04-BicapSqAntipr----ReC2----448	-690.48030020	39.68
267	01-TetrcTriPri1----ReC2----4	-690.48029570	39.69
268	01-TetrcTriPri1----ReC2----125	-690.48029250	39.69
269	01-TetrcTriPri1----ReC2----129	-690.48028480	39.69
270	02-TetrcTriPri2----ReC2----254	-690.48027360	39.70
271	02-TetrcTriPri2----ReC2----255	-690.48026930	39.70
272	02-TetrcTriPri2----ReC2----267	-690.48026930	39.70
273	04-BicapSqAntipr----ReC2----426	-690.48026490	39.70
274	04-BicapSqAntipr----ReC2----440	-690.48026490	39.70
275	01-TetrcTriPri1----ReC2----136	-690.48025830	39.71
276	01-TetrcTriPri1----ReC2----94	-690.48025830	39.71
277	01-TetrcTriPri1----ReC2----55	-690.48005940	39.83
278	01-TetrcTriPri1----ReC2----58	-690.48002480	39.86

279	01-TetrcTriPri1----ReC2----139	-690.47973490	40.04
280	01-TetrcTriPri1----ReC2----98	-690.47973490	40.04
281	02-TetrcTriPri2----ReC2----284	-690.47963140	40.10
282	04-BicapSqAntipr----ReC2----423	-690.47945420	40.21
283	04-BicapSqAntipr----ReC2----462	-690.47918870	40.38
284	01-TetrcTriPri1----ReC2----75	-690.47901410	40.49
285	04-BicapSqAntipr----ReC2----435	-690.47883200	40.60
286	02-TetrcTriPri2----ReC2----221	-690.47795870	41.15
287	02-TetrcTriPri2----ReC2----242	-690.47795870	41.15
288	03-BicapCub----ReC2----318	-690.47795420	41.15
289	03-BicapCub----ReC2----319	-690.47795360	41.16
290	07-Isocloso----ReC2----506	-690.47750310	41.44
291	07-Isocloso----ReC2----536	-690.47749350	41.44
292	01-TetrcTriPri1----ReC2----9	-690.47745920	41.47
293	07-Isocloso----ReC2----493	-690.47745660	41.47
294	07-Isocloso----ReC2----515	-690.47745390	41.47
295	02-TetrcTriPri2----ReC2----219	-690.47743990	41.48
296	02-TetrcTriPri2----ReC2----234	-690.47743990	41.48
297	01-TetrcTriPri1----ReC2----106	-690.47743160	41.48
298	01-TetrcTriPri1----ReC2----140	-690.47743160	41.48
299	02-TetrcTriPri2----ReC2----173	-690.47740850	41.50
300	02-TetrcTriPri2----ReC2----183	-690.47740810	41.50
301	03-BicapCub----ReC2----299	-690.47686330	41.84
302	01-TetrcTriPri1----ReC2----132	-690.47680030	41.88
303	07-Isocloso----ReC2----501	-690.47671400	41.93
304	04-BicapSqAntipr----ReC2----433	-690.47664050	41.98
305	04-BicapSqAntipr----ReC2----452	-690.47633450	42.17
306	01-TetrcTriPri1----ReC2----6	-690.47554130	42.67

307	01-TetrcTriPri1----ReC2----24	-690.47554060	42.67
308	05-PentagPrism----ReC2----477	-690.47550150	42.69
309	01-TetrcTriPri1----ReC2----67	-690.47550050	42.69
310	03-BicapCub----ReC2----297	-690.47547200	42.71
311	01-TetrcTriPri1----ReC2----40	-690.47545620	42.72
312	01-TetrcTriPri1----ReC2----3	-690.47545080	42.73
313	01-TetrcTriPri1----ReC2----64	-690.47543280	42.74
314	03-BicapCub----ReC2----307	-690.47519680	42.89
315	07-Isocloso----ReC2----542	-690.47510900	42.94
316	07-Isocloso----ReC2----575	-690.47510900	42.94
317	07-Isocloso----ReC2----538	-690.47510250	42.94
318	03-BicapCub----ReC2----301	-690.47500620	43.00
319	01-TetrcTriPri1----ReC2----101	-690.47374530	43.80
320	03-BicapCub----ReC2----310	-690.47125010	45.36
321	03-BicapCub----ReC2----343	-690.46905070	46.74
322	03-BicapCub----ReC2----346	-690.46905070	46.74
323	07-Isocloso----ReC2----541	-690.46882660	46.88
324	07-Isocloso----ReC2----543	-690.46880730	46.89
325	07-Isocloso----ReC2----567	-690.46880730	46.89
326	02-TetrcTriPri2----ReC2----236	-690.46759250	47.66
327	01-TetrcTriPri1----ReC2----89	-690.46573870	48.82
328	01-TetrcTriPri1----ReC2----128	-690.46573660	48.82
329	07-Isocloso----ReC2----545	-690.46547230	48.99
330	07-Isocloso----ReC2----579	-690.46547230	48.99
331	01-TetrcTriPri1----ReC2----81	-690.46543990	49.01
332	02-TetrcTriPri2----ReC2----181	-690.46543340	49.01
333	01-TetrcTriPri1----ReC2----53	-690.46538030	49.05
334	01-TetrcTriPri1----ReC2----69	-690.46538030	49.05

335	07-Isocloso----ReC2----562	-690.46537010	49.05
336	01-TetrcTriPri1----ReC2----49	-690.46536050	49.06
337	04-BicapSqAntipr----ReC2----465	-690.46534350	49.07
338	03-BicapCub----ReC2----345	-690.46534240	49.07
339	07-Isocloso----ReC2----573	-690.46531780	49.08
340	04-BicapSqAntipr----ReC2----379_r-14	-690.46510120	49.22
341	04-BicapSqAntipr----ReC2----386_r-14	-690.46510120	49.22
342	03-BicapCub----ReC2----298	-690.46504910	49.25
343	01-TetrcTriPri1----ReC2----73	-690.46469450	49.48
344	04-BicapSqAntipr----ReC2----379_i-14	-690.46464970	49.50
345	04-BicapSqAntipr----ReC2----386_i-14	-690.46464970	49.50
346	02-TetrcTriPri2----ReC2----223	-690.46449460	49.60
347	04-BicapSqAntipr----ReC2----445	-690.46448900	49.60
348	04-BicapSqAntipr----ReC2----451	-690.46448900	49.60
349	04-BicapSqAntipr----ReC2----454	-690.46439450	49.66
350	02-TetrcTriPri2----ReC2----179	-690.46437840	49.67
351	03-BicapCub----ReC2----347	-690.46354010	50.20
352	03-BicapCub----ReC2----367	-690.46354010	50.20
353	07-Isocloso----ReC2----533	-690.46330660	50.35
354	07-Isocloso----ReC2----514	-690.46330340	50.35
355	07-Isocloso----ReC2----513	-690.46288570	50.61
356	07-Isocloso----ReC2----529	-690.46286850	50.62
357	02-TetrcTriPri2----ReC2----208	-690.46285200	50.63
358	07-Isocloso----ReC2----569	-690.46280210	50.66
359	07-Isocloso----ReC2----581	-690.46280210	50.66
360	07-Isocloso----ReC2----577	-690.46277070	50.68
361	02-TetrcTriPri2----ReC2----245	-690.46276290	50.69
362	04-BicapSqAntipr----ReC2----461	-690.46216950	51.06

363	04-BicapSqAntipr----ReC2----453	-690.46216540	51.06
364	01-TetrcTriPri1----ReC2----138	-690.46215590	51.07
365	01-TetrcTriPri1----ReC2----154	-690.46215590	51.07
366	03-BicapCub----ReC2----356	-690.46211990	51.09
367	03-BicapCub----ReC2----372	-690.46211990	51.09
368	02-TetrcTriPri2----ReC2----187	-690.46199020	51.17
369	02-TetrcTriPri2----ReC2----207	-690.46199020	51.17
370	02-TetrcTriPri2----ReC2----182	-690.46059120	52.05
371	07-Isocloso----ReC2----490	-690.46057720	52.06
372	07-Isocloso----ReC2----510	-690.46057260	52.06
373	07-Isocloso----ReC2----530	-690.46057200	52.06
374	07-Isocloso----ReC2----520	-690.46056830	52.06
375	02-TetrcTriPri2----ReC2----237	-690.46035420	52.20
376	02-TetrcTriPri2----ReC2----244	-690.46035350	52.20
377	01-TetrcTriPri1----ReC2----109	-690.45954860	52.70
378	01-TetrcTriPri1----ReC2----133	-690.45954860	52.70
379	04-BicapSqAntipr----ReC2----434	-690.45952640	52.72
380	04-BicapSqAntipr----ReC2----442	-690.45952640	52.72
381	01-TetrcTriPri1----ReC2----12	-690.45939880	52.80
382	05-PentagPrism----ReC2----475	-690.45935620	52.83
383	07-Isocloso----ReC2----558	-690.45935250	52.83
384	02-TetrcTriPri2----ReC2----222	-690.45858110	53.31
385	04-BicapSqAntipr----ReC2----437	-690.45848090	53.37
386	04-BicapSqAntipr----ReC2----450	-690.45848090	53.37
387	01-TetrcTriPri1----ReC2----157	-690.45847170	53.38
388	01-TetrcTriPri1----ReC2----131	-690.45845170	53.39
389	05-PentagPrism----ReC2----478	-690.45843820	53.40
390	01-TetrcTriPri1----ReC2----116	-690.45841590	53.42

391	07-Isocloso----ReC2----540	-690.45841070	53.42
392	07-Isocloso----ReC2----551	-690.45841070	53.42
393	04-BicapSqAntipr----ReC2----436	-690.45777690	53.82
394	04-BicapSqAntipr----ReC2----458	-690.45777690	53.82
395	02-TetrcTriPri2----ReC2----214	-690.45776000	53.83
396	02-TetrcTriPri2----ReC2----209	-690.45741380	54.04
397	02-TetrcTriPri2----ReC2----217	-690.45741380	54.04
398	03-BicapCub----ReC2----325	-690.45722880	54.16
399	03-BicapCub----ReC2----376	-690.45722880	54.16
400	06-AntiPr----ReC2----487	-690.45592500	54.98
401	03-BicapCub----ReC2----300	-690.45587880	55.01
402	06-AntiPr----ReC2----486	-690.45544470	55.28
403	03-BicapCub----ReC2----311	-690.45505200	55.53
404	07-Isocloso----ReC2----552	-690.45504870	55.53
405	07-Isocloso----ReC2----556	-690.45504870	55.53
406	07-Isocloso----ReC2----547	-690.45499480	55.56
407	02-TetrcTriPri2----ReC2----213	-690.45456420	55.83
408	02-TetrcTriPri2----ReC2----241	-690.45456420	55.83
409	02-TetrcTriPri2----ReC2----176	-690.45357440	56.45
410	02-TetrcTriPri2----ReC2----212	-690.45287240	56.89
411	03-BicapCub----ReC2----326	-690.45261470	57.06
412	03-BicapCub----ReC2----336	-690.45261470	57.06
413	07-Isocloso----ReC2----560	-690.45101290	58.06
414	07-Isocloso----ReC2----539	-690.45095900	58.09
415	05-PentagPrism----ReC2----480	-690.45091350	58.12
416	07-Isocloso----ReC2----570	-690.45079810	58.20
417	03-BicapCub----ReC2----304	-690.45054160	58.36
418	03-BicapCub----ReC2----313	-690.45054100	58.36

419	04-BicapSqAntipr----ReC2----400	-690.45019610	58.57
420	04-BicapSqAntipr----ReC2----419	-690.45019610	58.57
421	03-BicapCub----ReC2----303	-690.45014390	58.61
422	03-BicapCub----ReC2----305	-690.45014060	58.61
423	01-TetrcTriPri1----ReC2----56	-690.44926040	59.16
424	01-TetrcTriPri1----ReC2----62	-690.44926040	59.16
425	04-BicapSqAntipr----ReC2----392	-690.44829150	59.77
426	04-BicapSqAntipr----ReC2----420	-690.44829150	59.77
427	04-BicapSqAntipr----ReC2----384	-690.44822470	59.81
428	04-BicapSqAntipr----ReC2----418	-690.44822470	59.81
429	01-TetrcTriPri1----ReC2----92	-690.44793590	59.99
430	01-TetrcTriPri1----ReC2----104	-690.44793280	59.99
431	03-BicapCub----ReC2----351	-690.44715450	60.48
432	06-AntiPr----ReC2----483	-690.44713750	60.49
433	03-BicapCub----ReC2----327	-690.44698500	60.59
434	01-TetrcTriPri1----ReC2----44	-690.44595370	61.24
435	01-TetrcTriPri1----ReC2----85	-690.44399050	62.47
436	01-TetrcTriPri1----ReC2----70	-690.44398730	62.47
437	01-TetrcTriPri1----ReC2----83	-690.44397840	62.48
438	04-BicapSqAntipr----ReC2----417	-690.44172140	63.89
439	01-TetrcTriPri1----ReC2----50	-690.44158880	63.97
440	07-Isocloso----ReC2----548	-690.44036410	64.74
441	07-Isocloso----ReC2----550	-690.44031690	64.77
442	07-Isocloso----ReC2----564	-690.44031690	64.77
443	03-BicapCub----ReC2----361	-690.43968370	65.17
444	01-TetrcTriPri1----ReC2----72	-690.43943570	65.33
445	01-TetrcTriPri1----ReC2----61	-690.43943460	65.33
446	07-Isocloso----ReC2----555	-690.43865910	65.81

447	03-BicapCub----ReC2----349	-690.43609430	67.42
448	03-BicapCub----ReC2----375	-690.43609430	67.42
449	01-TetrcTriPri1----ReC2----100	-690.43607820	67.43
450	01-TetrcTriPri1----ReC2----108	-690.43607820	67.43
451	03-BicapCub----ReC2----341	-690.43583250	67.59
452	03-BicapCub----ReC2----330	-690.43583210	67.59
453	03-BicapCub----ReC2----340	-690.43575320	67.64
454	03-BicapCub----ReC2----370	-690.43575320	67.64
455	03-BicapCub----ReC2----331	-690.43561650	67.72
456	03-BicapCub----ReC2----365	-690.43561650	67.72
457	03-BicapCub----ReC2----335	-690.43459700	68.36
458	03-BicapCub----ReC2----350	-690.43459700	68.36
459	02-TetrcTriPri2----ReC2----271	-690.43432450	68.53
460	03-BicapCub----ReC2----324	-690.43412460	68.66
461	03-BicapCub----ReC2----360	-690.43412460	68.66
462	03-BicapCub----ReC2----342	-690.43403970	68.71
463	04-BicapSqAntipr----ReC2----443	-690.43402490	68.72
464	01-TetrcTriPri1----ReC2----35	-690.43135270	70.40
465	01-TetrcTriPri1----ReC2----13	-690.43007960	71.20
466	03-BicapCub----ReC2----332	-690.42947620	71.58
467	03-BicapCub----ReC2----357	-690.42947280	71.58
468	06-AntiPr----ReC2----484	-690.42894730	71.91
469	03-BicapCub----ReC2----314_r-97	-690.42857070	72.14
470	03-BicapCub----ReC2----323	-690.42855880	72.15
471	03-BicapCub----ReC2----368	-690.42855880	72.15
472	03-BicapCub----ReC2----314_i-97	-690.42807260	72.46
473	06-AntiPr----ReC2----481	-690.42724020	72.98
474	03-BicapCub----ReC2----374	-690.42641700	73.50

475	03-BicapCub----ReC2----322	-690.42609610	73.70
476	03-BicapCub----ReC2----344	-690.42609610	73.70
477	01-TetrcTriPri1----ReC2----37	-690.42552160	74.06
478	03-BicapCub----ReC2----369	-690.42529340	74.20
479	04-BicapSqAntipr----ReC2----449	-690.42525570	74.22
480	01-TetrcTriPri1----ReC2----19	-690.42509500	74.32
481	03-BicapCub----ReC2----358	-690.42505940	74.35
482	04-BicapSqAntipr----ReC2----421	-690.42478610	74.52
483	03-BicapCub----ReC2----317	-690.42417020	74.91
484	04-BicapSqAntipr----ReC2----408	-690.42413400	74.93
485	01-TetrcTriPri1----ReC2----21	-690.42381160	75.13
486	04-BicapSqAntipr----ReC2----425	-690.42375430	75.17
487	04-BicapSqAntipr----ReC2----396	-690.42303850	75.62
488	03-BicapCub----ReC2----306_i-18	-690.42209650	76.21
489	03-BicapCub----ReC2----306_r-18	-690.42207410	76.22
490	05-PentagPrism----ReC2----473	-690.42149750	76.58
491	04-BicapSqAntipr----ReC2----399	-690.42130090	76.71
492	04-BicapSqAntipr----ReC2----424	-690.42124650	76.74
493	04-BicapSqAntipr----ReC2----416	-690.42121710	76.76
494	04-BicapSqAntipr----ReC2----422	-690.42121710	76.76
495	04-BicapSqAntipr----ReC2----383	-690.42075400	77.05
496	04-BicapSqAntipr----ReC2----388	-690.42072250	77.07
497	04-BicapSqAntipr----ReC2----389	-690.42068760	77.09
498	04-BicapSqAntipr----ReC2----412	-690.42068760	77.09
499	04-BicapSqAntipr----ReC2----382	-690.42063600	77.12
500	04-BicapSqAntipr----ReC2----402	-690.42063600	77.12
501	01-TetrcTriPri1----ReC2----38	-690.41848120	78.48
502	06-AntiPr----ReC2----485	-690.41803360	78.76

503	04-BicapSqAntipr----ReC2----405	-690.41676710	79.55
504	04-BicapSqAntipr----ReC2----415	-690.41417940	81.17
505	04-BicapSqAntipr----ReC2----401	-690.41413230	81.20
506	04-BicapSqAntipr----ReC2----409	-690.41409720	81.23
507	01-TetrcTriPri1----ReC2----22	-690.41406860	81.24
508	04-BicapSqAntipr----ReC2----441	-690.41343420	81.64
509	07-Isocloso----ReC2----561	-690.41315600	81.82
510	07-Isocloso----ReC2----582	-690.41315600	81.82
511	04-BicapSqAntipr----ReC2----446	-690.41310990	81.85
512	07-Isocloso----ReC2----580	-690.41310630	81.85
513	03-BicapCub----ReC2----339	-690.41302820	81.90
514	03-BicapCub----ReC2----354	-690.41302820	81.90
515	03-BicapCub----ReC2----334	-690.41293650	81.95
516	03-BicapCub----ReC2----333	-690.41280990	82.03
517	03-BicapCub----ReC2----373	-690.41280990	82.03
518	07-Isocloso----ReC2----544	-690.41205460	82.51
519	03-BicapCub----ReC2----364	-690.41146440	82.88
520	03-BicapCub----ReC2----371	-690.41146440	82.88
521	03-BicapCub----ReC2----338	-690.41143380	82.90
522	03-BicapCub----ReC2----362	-690.41143380	82.90
523	03-BicapCub----ReC2----302	-690.41090720	83.23
524	01-TetrcTriPri1----ReC2----42	-690.41085600	83.26
525	01-TetrcTriPri1----ReC2----31	-690.41085520	83.26
526	01-TetrcTriPri1----ReC2----102	-690.41019380	83.68
527	01-TetrcTriPri1----ReC2----105	-690.40763470	85.28
528	01-TetrcTriPri1----ReC2----124	-690.40763470	85.28
529	04-BicapSqAntipr----ReC2----393	-690.40726460	85.51
530	03-BicapCub----ReC2----353	-690.40577190	86.45

531	03-BicapCub----ReC2----337	-690.40529690	86.75
532	03-BicapCub----ReC2----312	-690.40477450	87.08
533	03-BicapCub----ReC2----320	-690.40477450	87.08
534	03-BicapCub----ReC2----348	-690.40475610	87.09
535	03-BicapCub----ReC2----359	-690.40475610	87.09
536	01-TetrcTriPri1----ReC2----20	-690.40357520	87.83
537	01-TetrcTriPri1----ReC2----15	-690.40264370	88.41
538	01-TetrcTriPri1----ReC2----45	-690.40264370	88.41
539	04-BicapSqAntipr----ReC2----387	-690.40257750	88.46
540	04-BicapSqAntipr----ReC2----395	-690.40257750	88.46
541	04-BicapSqAntipr----ReC2----378	-690.40252820	88.49
542	04-BicapSqAntipr----ReC2----394	-690.40252820	88.49
543	01-TetrcTriPri1----ReC2----103	-690.40221270	88.68
544	01-TetrcTriPri1----ReC2----148	-690.40221270	88.68
545	04-BicapSqAntipr----ReC2----413_r-20	-690.40139760	89.20
546	02-TetrcTriPri2----ReC2----203	-690.40080850	89.57
547	02-TetrcTriPri2----ReC2----163	-690.40080800	89.57
548	03-BicapCub----ReC2----309	-690.40064850	89.67
549	04-BicapSqAntipr----ReC2----377	-690.39936530	90.47
550	04-BicapSqAntipr----ReC2----404	-690.39898130	90.71
551	04-BicapSqAntipr----ReC2----390	-690.39897800	90.71
552	04-BicapSqAntipr----ReC2----398	-690.39896390	90.72
553	04-BicapSqAntipr----ReC2----403	-690.39896390	90.72
554	04-BicapSqAntipr----ReC2----397	-690.39893180	90.74
555	04-BicapSqAntipr----ReC2----411	-690.39893180	90.74
556	04-BicapSqAntipr----ReC2----391	-690.39885930	90.79
557	04-BicapSqAntipr----ReC2----407	-690.39806150	91.29
558	04-BicapSqAntipr----ReC2----413_i-20	-690.39787820	91.40

559	04-BicapSqAntipr----ReC2----381	-690.39783610	91.43
560	04-BicapSqAntipr----ReC2----410	-690.39783610	91.43
561	04-BicapSqAntipr----ReC2----406	-690.39763950	91.55
562	04-BicapSqAntipr----ReC2----414	-690.39763950	91.55
563	01-TetrcTriPri1----ReC2----10	-690.39619020	92.46
564	01-TetrcTriPri1----ReC2----28	-690.39619020	92.46
565	04-BicapSqAntipr----ReC2----385	-690.39574080	92.75
566	04-BicapSqAntipr----ReC2----380	-690.39552940	92.88
567	01-TetrcTriPri1----ReC2----112	-690.39087590	95.80
568	01-TetrcTriPri1----ReC2----93	-690.39087410	95.80
569	01-TetrcTriPri1----ReC2----14	-690.38549420	99.18
570	01-TetrcTriPri1----ReC2----39	-690.38311030	100.67
571	01-TetrcTriPri1----ReC2----43	-690.38311030	100.67
572	01-TetrcTriPri1----ReC2----17	-690.38134870	101.78
573	01-TetrcTriPri1----ReC2----41	-690.37486750	105.84
574	01-TetrcTriPri1----ReC2----147	-690.37471770	105.94
575	01-TetrcTriPri1----ReC2----159	-690.37471770	105.94
576	01-TetrcTriPri1----ReC2----113	-690.36866860	109.73
577	01-TetrcTriPri1----ReC2----126	-690.36866860	109.73
578	01-TetrcTriPri1----ReC2----155	-690.36593450	111.45
579	01-TetrcTriPri1----ReC2----99	-690.36593450	111.45
580	01-TetrcTriPri1----ReC2----34	-690.36225090	113.76
581	01-TetrcTriPri1----ReC2----26	-690.36225010	113.76
582	01-TetrcTriPri1----ReC2----123	-690.34748530	123.03
583	01-TetrcTriPri1----ReC2----97	-690.34748530	123.03
584	01-TetrcTriPri1----ReC2----110	-690.34305280	125.81
585	01-TetrcTriPri1----ReC2----117	-690.34301760	125.83
586	01-TetrcTriPri1----ReC2----115	-690.34128110	126.92

587	01-TetrcTriPri1----ReC2----158	-690.34128110	126.92
588	01-TetrcTriPri1----ReC2----18	-690.33749380	129.30
589	01-TetrcTriPri1----ReC2----29	-690.33749380	129.30
590	01-TetrcTriPri1----ReC2----16	-690.26994310	171.69
591	01-TetrcTriPri1----ReC2----5	-690.26994310	171.69

Table 4A. Initial $(CO)_2(NO)ReC_2B_8H_{10}$ structures (one example from each family), a total of 984 structures:

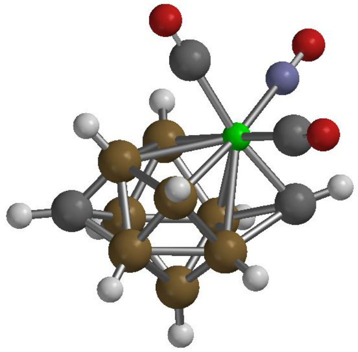<br>1. Pentacapped trigonal prism 88 structures
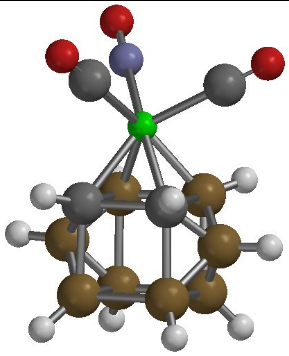<br>2. Tricapped cube 384 structures
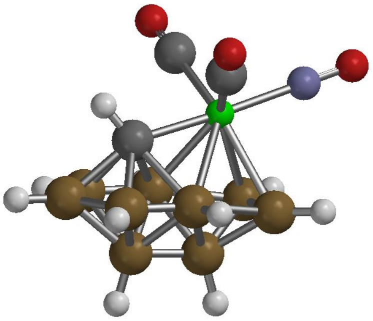<br>2b. Octahedron fused to pentag pyr 240 structures
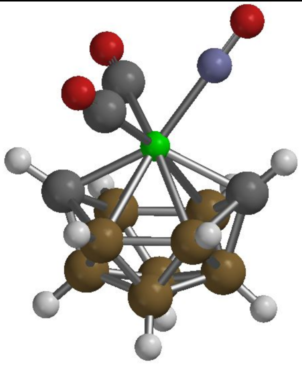

4. Edge-coalesced icosahedrons (icosahedron without a vertex) 272 structures

Table 4B. Distances table for the lowest-lying $(CO)_2(NO)ReC_2B_8H_{10}$ structures after M06L/6-311G(d,p)//SDD optimization. Included are the ZPcorrected E (a.u.), relative energy (kcal/mol) and symmetry:

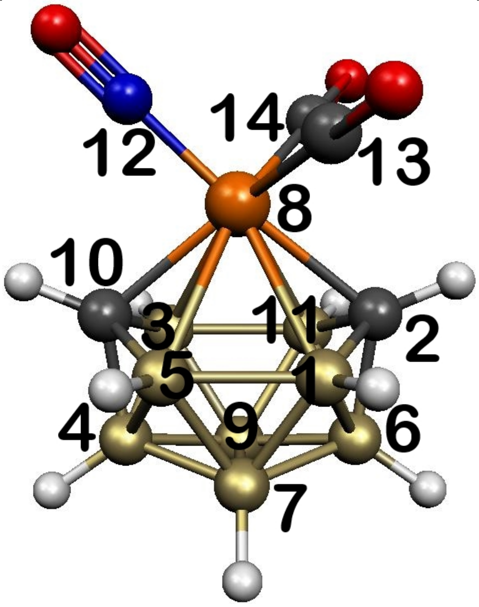

1. -716.2067546 0.0 C_{2v}-C_S

		1	2	3	4	5
1	B	0.000000				
2	C	1.582223	0.000000			
3	B	3.229983	2.970704	0.000000		
4	B	2.950520	3.523009	1.815375	0.000000	
5	B	1.874859	2.968891	2.635478	1.816733	0.000000
6	B	1.809933	1.630489	2.962094	2.907828	2.962128
7	B	1.769298	2.711785	2.816857	1.739676	1.778718
8	Re	2.477798	2.205630	2.446898	3.309739	2.444604
9	B	2.808193	2.711907	1.775948	1.739968	2.817781
10	C	2.971847	3.368744	1.585585	1.619611	1.584732
11	B	2.620447	1.581802	1.872352	2.947417	3.224941
12	N	4.110567	4.047353	3.424478	4.269376	3.421802
13	C	2.818847	2.835125	4.378193	4.851357	3.396677
14	C	3.941935	2.836655	3.386296	4.842573	4.373674

		6	7	8	9	10
6	B	0.000000				
7	B	1.742887	0.000000			
8	Re	3.348255	3.428918	0.000000		
9	B	1.743550	1.813098	3.428102	0.000000	
10	C	3.530064	2.709376	2.157148	2.708907	0.000000
11	B	1.810212	2.805481	2.473394	1.767487	2.967442
12	N	5.123240	4.868495	1.845486	4.867781	2.684919
13	C	4.233180	4.402928	2.004332	4.961486	3.828295
14	C	4.229658	4.957715	2.004022	4.391868	3.820649

		11	12	13	14
11	B	0.000000			
12	N	4.106575	0.000000		

13	C	3.936640	2.772088	0.000000	
14	C	2.807878	2.772329	2.901427	0.000000

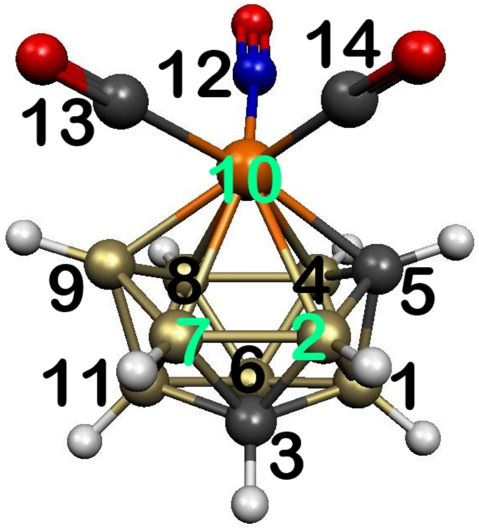

2. -716.1962432 +6.6 C_1

		1	2	3	4	5
1	B	0.000000				
2	B	1.804756	0.000000			
3	C	1.661702	1.682317	0.000000		
4	B	1.795349	2.623072	2.669712	0.000000	
5	C	1.628811	1.593765	2.597044	1.584142	0.000000
6	B	1.724758	2.789918	1.715184	1.761216	2.706430
7	B	2.919601	1.817119	1.692473	3.214987	2.939150
8	B	2.938379	3.214716	2.679848	1.871277	2.965331
9	B	3.704359	3.123130	2.776650	3.136626	3.537008
10	Re	3.302589	2.459128	3.266968	2.443215	2.175834
11	B	2.884206	2.906118	1.671912	2.934142	3.498826
12	N	4.506526	4.138683	4.817909	3.027522	3.271226
13	C	5.043724	3.813602	4.463639	4.337589	4.084933
14	C	4.223046	2.944767	4.432230	3.851638	2.746933

		6	7	8	9	10
6	B	0.000000				
7	B	2.802326	0.000000			
8	B	1.766077	2.643827	0.000000		
9	B	2.872329	1.732142	1.699084	0.000000	
10	Re	3.384605	2.429707	2.415994	2.224071	0.000000
11	B	1.742892	1.797430	1.796218	1.705771	3.247458
12	N	4.368717	4.164493	3.057756	3.374703	1.837821
13	C	4.869705	2.970695	3.603630	2.448335	2.007699
14	C	4.978658	3.602032	4.398183	4.046726	2.041704

		11	12	13	14
11	B	0.000000			

12	N	4.522930	0.000000		
13	C	4.026495	2.825605	0.000000	
14	C	4.951613	2.935678	2.788406	0.000000

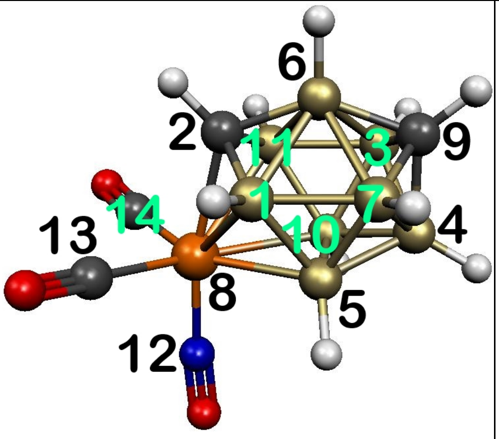

3. -716.1954398 +7.1 C_S

		1	2	3	4	5
1	B	0.000000				
2	C	1.582209	0.000000			
3	B	3.218291	2.841047	0.000000		
4	B	2.939079	3.446048	1.793459	0.000000	
5	B	1.793775	2.766509	2.823263	1.759020	0.000000
6	B	1.986922	1.624494	1.974610	2.662378	2.778088
7	B	1.832579	2.842434	2.648976	1.792538	1.754280
8	Re	2.325094	2.212323	3.617190	3.600542	2.320995
9	C	2.831390	3.043160	1.562945	1.632893	2.679164
10	B	2.844568	2.766394	1.755334	1.757096	1.844185
11	B	2.643283	1.581564	1.831016	2.938572	2.845701
12	N	3.804375	4.023600	4.753110	4.314796	3.046448
13	C	2.827024	3.051765	5.315041	5.140291	3.532618
14	C	3.959401	3.067090	4.539205	5.134045	4.214290

		6	7	8	9	10
6	B	0.000000				
7	B	1.973614	0.000000			
8	Re	3.398889	3.616180	0.000000		
9	C	1.618270	1.562487	4.168203	0.000000	
10	B	2.778852	2.821042	2.322276	2.678457	0.000000
11	B	1.988382	3.218315	2.327557	2.830975	1.795147
12	N	5.007247	4.741816	1.833513	5.386378	3.058056
13	C	4.456610	4.542457	1.994497	5.485360	4.217484
14	C	4.467806	5.319207	1.994797	5.487796	3.523143

		11	12	13	14

11	B	0.000000			
12	N	3.818611	0.000000		
13	C	3.949718	2.827174	0.000000	
14	C	2.830645	2.828423	2.889081	0.000000

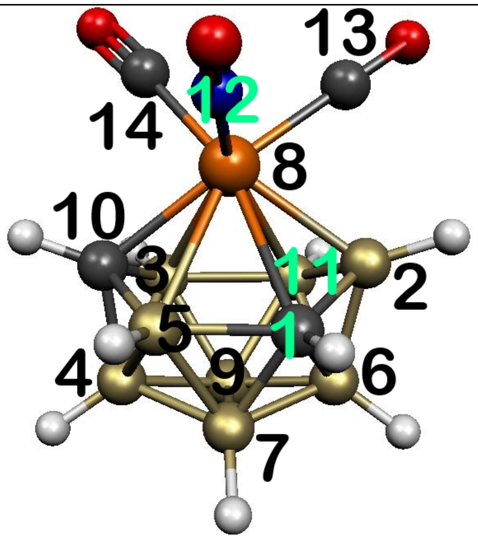

4. -716.1897511 +10.7 C_1

		1	2	3	4	5
1	C	0.000000				
2	B	1.601453	0.000000			
3	B	3.081586	3.105445	0.000000		
4	B	2.819108	3.665076	1.803461	0.000000	
5	B	1.771360	3.040915	2.619722	1.807775	0.000000
6	B	1.731098	1.710250	2.905391	2.886996	2.920384
7	B	1.679794	2.821207	2.793430	1.739028	1.773141
8	Re	2.382041	2.224102	2.460926	3.300463	2.417630
9	B	2.689659	2.878365	1.766013	1.743128	2.808502
10	C	2.849371	3.486770	1.609137	1.630388	1.578196
11	B	2.527239	1.731229	1.839029	2.940202	3.202494
12	N	3.177453	3.417140	4.147493	4.531146	3.053641
13	C	3.555118	2.435070	3.796082	5.021754	4.289376
14	C	4.320073	4.010789	2.944263	4.228981	3.853759

		6	7	8	9	10
6	B	0.000000				
7	B	1.729316	0.000000			
8	Re	3.289318	3.408307	0.000000		
9	B	1.726470	1.799454	3.434986	0.000000	
10	C	3.503760	2.717702	2.151965	2.734334	0.000000
11	B	1.793131	2.803609	2.455469	1.784449	2.960537
12	N	4.625808	4.455372	1.842682	5.029609	3.248538
13	C	4.011394	4.846506	2.011364	4.560706	4.065324
14	C	4.948587	4.990431	2.006961	4.548057	2.763837

		11	12	13	14
11	B	0.000000			
12	N	4.210818	0.000000		
13	C	2.933671	2.846834	0.000000	
14	C	3.590082	2.866025	2.760710	0.000000

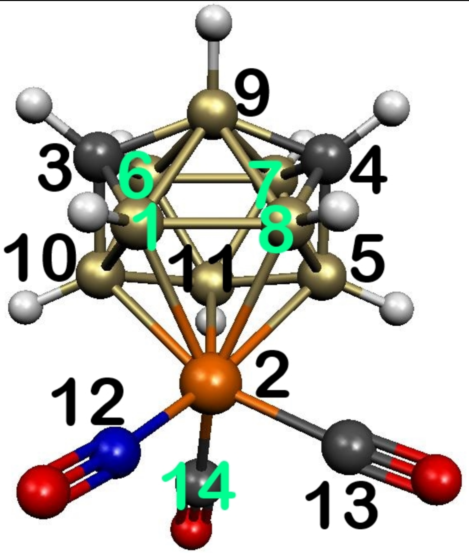

5. -716.1887825 +11.3 (Cs)

		1	2	3	4	5
1	B	0.000000				
2	Re	2.300107	0.000000			
3	C	1.560516	3.356095	0.000000		
4	C	2.830757	3.356437	3.014419	0.000000	
5	B	3.013232	2.328290	3.436200	1.642143	0.000000
6	B	2.662045	3.540651	1.561474	2.810431	2.929636
7	B	3.230997	3.549625	2.806388	1.559598	1.773530
8	B	1.860596	2.315518	2.841248	1.560063	1.877916
9	B	1.993925	3.535379	1.617145	1.619264	2.694848
10	B	1.858810	2.325902	1.637832	3.437459	3.026218
11	B	2.863446	2.403463	2.696922	2.692678	1.783645
12	N	2.853532	1.840536	4.115857	4.876788	4.124536
13	C	3.799936	1.990922	5.038671	3.892022	2.807804
14	C	4.061118	1.988279	4.699662	4.790398	3.329190

		6	7	8	9	10
6	B	0.000000				
7	B	1.808655	0.000000			
8	B	3.243910	2.673121	0.000000		
9	B	1.971087	1.964521	1.998692	0.000000	
10	B	1.768871	2.939027	3.026980	2.692226	0.000000
11	B	1.764822	1.771004	2.877564	2.776573	1.798918
12	N	4.787912	5.206435	3.524099	4.692570	3.179382
13	C	5.152559	4.525854	2.860725	4.697947	4.259200

14 C	4.392486	4.462861	4.141564	5.085162	3.200130

	11	12	13	14
11 B	0.000000			
12 N	4.017082	0.000000		
13 C	3.823344	2.876728	0.000000	
14 C	2.858359	2.850068	2.874432	0.000000

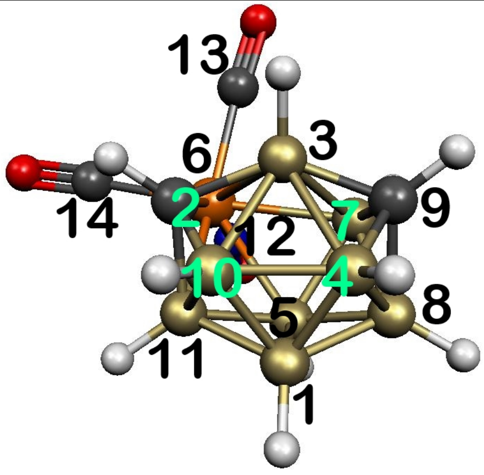

6. -716.1870837 +12.3 C_1

	1	2	3	4	5
1 B	0.000000				
2 C	2.710101	0.000000			
3 B	2.783612	1.622172	0.000000		
4 B	1.755518	2.837942	2.012284	0.000000	
5 B	1.799712	2.756493	2.820593	2.821060	0.000000
6 Re	3.551637	2.143772	2.648453	4.021366	2.324828
7 B	2.815557	2.920798	2.067552	2.665802	1.791845
8 B	1.748594	3.434873	2.699059	1.767669	1.778152
9 C	2.673361	3.020027	1.622007	1.567031	2.695869
10 B	1.752218	1.568245	1.983522	1.810348	2.817942
11 B	1.778277	1.664932	2.683044	2.931106	1.801492
12 N	4.606945	3.918237	4.298721	5.368470	2.921664
13 C	5.095825	3.252850	3.120190	4.961037	3.956759
14 C	4.882992	2.850221	4.008566	5.495028	3.989493

	6	7	8	9	10
6 Re	0.000000				
7 B	2.365741	0.000000			
8 B	3.625544	1.777433	0.000000		
9 C	3.477046	1.558942	1.649554	0.000000	
10 B	3.382781	3.249436	2.898843	2.791870	0.000000
11 B	2.349966	2.982392	2.954907	3.397129	1.780790
12 N	1.837938	3.188878	4.415377	4.670553	4.961635

13	C	1.977987	3.052094	4.735553	3.955217	4.619120
14	C	1.990346	4.328859	5.429088	5.235572	4.288855

		11	12	13	14
11	B	0.000000			
12	N	3.567636	0.000000		
13	C	4.142183	2.843636	0.000000	
14	C	3.210122	2.823073	2.822932	0.000000

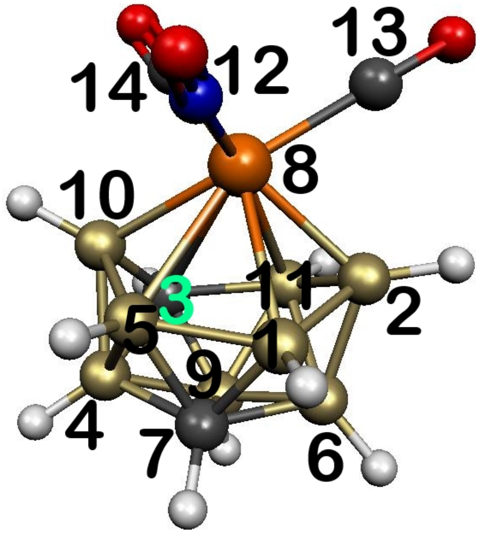

7. -716.1808960 +16.2 C_1

3-8 fragile edge

		1	2	3	4	5
1	B	0.000000				
2	B	1.733047	0.000000			
3	C	3.261727	3.151551	0.000000		
4	B	2.926919	3.667321	1.668303	0.000000	
5	B	1.839471	3.045132	2.589165	1.771064	0.000000
6	B	1.779285	1.698375	2.830370	2.876810	2.878749
7	C	1.691780	2.760180	2.557116	1.655932	1.703745
8	Re	2.372753	2.163363	2.889828	3.424256	2.367027
9	B	2.837634	2.900837	1.612591	1.743023	2.788229
10	B	3.087915	3.488991	1.592525	1.729337	1.725407
11	B	2.664606	1.750567	1.709804	2.820815	3.045627
12	N	3.175656	3.571035	4.453786	4.519522	2.962514
13	C	3.585438	2.483680	4.331342	5.218454	4.278226
14	C	4.274278	3.821312	3.175619	4.179046	3.682159

		6	7	8	9	10
6	B	0.000000				
7	C	1.666437	0.000000			
8	Re	3.275204	3.301346	0.000000		
9	B	1.745634	1.701514	3.612343	0.000000	
10	B	3.530481	2.735540	2.287668	2.765046	0.000000
11	B	1.874790	2.647827	2.491481	1.801126	2.711382

12	N	4.654523	4.333853	1.833423	5.134836	3.332122
13	C	4.138190	4.829289	2.014167	4.885623	4.171302
14	C	4.803257	4.744000	2.015207	4.532446	2.553733

		11	12	13	14
11	B	0.000000			
12	N	4.280225	0.000000		
13	C	3.260666	2.873810	0.000000	
14	C	3.351675	2.925198	2.831055	0.000000

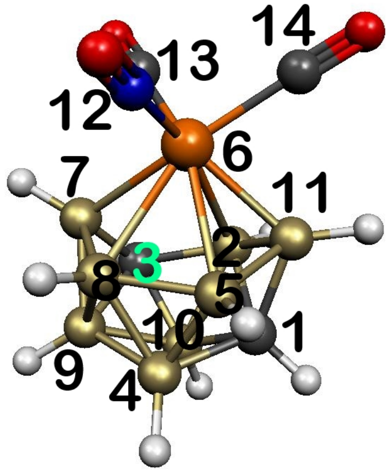

8. -716.1803568 +16.6 C_1

3-6 fragile edge

		1	2	3	4	5
1	C	0.000000				
2	B	1.796800	0.000000			
3	C	2.697600	1.671125	0.000000		
4	B	1.660712	2.785841	2.697398	0.000000	
5	B	1.697311	2.670860	3.289304	1.750461	0.000000
6	Re	3.150970	2.493593	3.006012	3.469795	2.373826
7	B	3.333352	2.592600	1.591630	2.855601	3.064656
8	B	2.730071	2.994372	2.617900	1.791106	1.815961
9	B	2.759085	2.789645	1.669031	1.734947	2.930513
10	B	1.678595	1.833602	1.609967	1.780025	2.845949
11	B	1.618542	1.742022	3.149267	2.864469	1.755575
12	N	4.552467	4.279948	4.516380	4.435335	3.195190
13	C	4.630495	3.273426	3.258614	4.906146	4.256341
14	C	4.107308	3.389440	4.510756	5.004896	3.587647

		6	7	8	9	10
6	Re	0.000000				
7	B	2.319008	0.000000			
8	B	2.377483	1.734602	0.000000		
9	B	3.492230	1.745143	1.793842	0.000000	
10	B	3.686234	2.749779	2.793373	1.737535	0.000000

		1	2	3	4	5
11	B	2.152154	3.424240	3.020513	3.657432	2.898138
12	N	1.837682	3.319419	2.927283	4.520814	5.168957
13	C	2.009044	2.582645	3.692228	4.235253	4.574880
14	C	2.011582	4.220161	4.280453	5.314402	5.023551

		11	12	13	14
11	B	0.000000			
12	N	3.631556	0.000000		
13	C	3.752374	2.926069	0.000000	
14	C	2.538206	2.874116	2.849025	0.000000

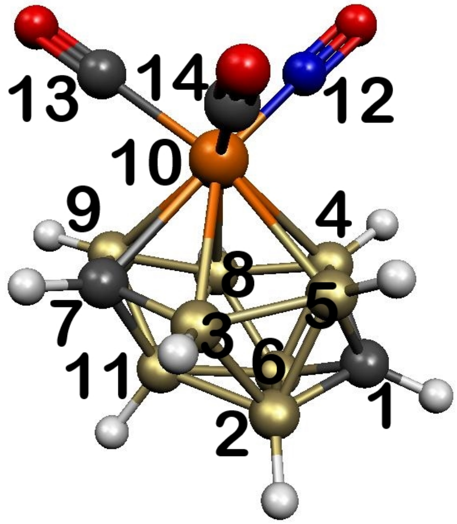

9. -716.1784069 +17.8 C_1

		1	2	3	4	5
1	C	0.000000				
2	B	1.574743	0.000000			
3	B	2.800737	1.771137	0.000000		
4	B	1.583635	2.562015	3.000078	0.000000	
5	B	1.587452	1.793018	1.807435	1.877780	0.000000
6	B	1.576893	1.795242	2.930409	1.780152	2.561598
7	C	3.780862	2.847851	1.561928	3.446397	3.002192
8	B	2.779004	2.912398	3.017932	1.778106	2.959924
9	B	3.920129	3.415027	2.735270	3.131957	3.557187
10	Re	3.455940	3.474829	2.444232	2.430355	2.410396
11	B	2.823173	1.869386	1.954410	2.836815	2.840663
12	N	4.103618	4.733222	4.091187	2.748125	3.319553
13	C	5.355637	5.154491	3.854777	4.211341	4.367647
14	C	4.455691	4.283193	2.858877	3.912930	2.929053

		6	7	8	9	10
6	B	0.000000				
7	C	3.285320	0.000000			
8	B	1.759805	2.635522	0.000000		
9	B	2.980631	1.527180	1.676826	0.000000	

		6	7	8	9	10
10	Re	3.475056	2.265844	2.425481	2.317969	0.000000
11	B	1.862557	1.722238	1.941160	1.825813	3.056495
12	N	4.355922	4.075634	3.288139	3.753997	1.833522
13	C	5.014484	2.890775	3.524671	2.618572	2.003009
14	C	4.966384	3.186098	4.321358	3.963127	1.998819

		11	12	13	14
11	B	0.000000			
12	N	4.571110	0.000000		
13	C	4.148229	2.812585	0.000000	
14	C	4.376752	2.848459	2.890001	0.000000

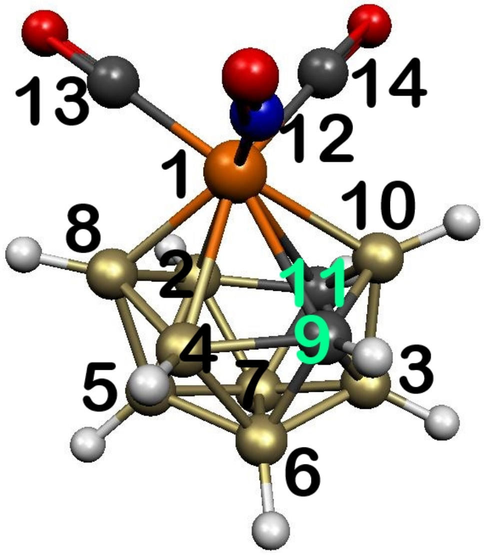

10. -716.1760563 +19.3 (C_s)

		1	2	3	4	5
1	Re	0.000000				
2	B	2.474383	0.000000			
3	B	3.440594	2.833783	0.000000		
4	B	2.353693	2.657315	2.915240	0.000000	
5	B	3.260996	1.856505	2.874058	1.799910	0.000000
6	B	3.451599	2.790808	1.718916	1.770957	1.740237
7	B	3.599060	1.803279	1.727805	2.840135	1.751754
8	B	2.149637	1.757812	3.689060	1.744383	1.707186
9	C	2.367161	2.944808	1.706999	1.748736	2.764595
10	B	2.265258	2.757006	1.732828	2.995374	3.510177
11	C	2.799306	1.712716	1.670592	3.198715	2.821962
12	N	1.836705	4.261215	4.587718	3.124660	4.623065
13	C	1.992300	3.201469	5.202567	3.612596	4.128585
14	C	2.025520	3.398025	4.188186	4.261548	4.823577

		6	7	8	9	10
6	B	0.000000				
7	B	1.785345	0.000000			
8	B	2.893840	2.923531	0.000000		

	9 C 1.693377 2.664124 2.971853 0.000000<br>10 B 2.816203 2.774977 3.484112 1.616990 0.000000<br>11 C 2.678726 1.643094 3.130278 2.442316 1.589317<br>12 N 4.462692 5.141684 3.501029 3.072552 3.334610<br>13 C 4.975734 4.845944 2.483353 4.257544 4.131408<br>14 C 4.904796 4.541996 3.862477 3.672255 2.572620<br>11 12 13 14<br>11 C 0.000000<br>12 N 4.399476 0.000000<br>13 C 4.206425 2.833574 0.000000<br>14 C 3.113020 2.940334 2.805030 0.000000

Table 4C. Energy ranking for $(CO)_2(NO)ReC_2B_8H_{10}$ obtained after B3LYP/6-31G(d)//SDD optimization.

No	Initial structure	Final energy (a.u.)	ΔE (kcal/mol)
1	04-Icos-1vx----ReC2------776	-715.9864839	0.00
2	04-Icos-1vx----ReC2------746	-715.9864807	0.00
3	02-TricapCubeB----ReC2------296	-715.9864586	0.02
4	02-TricapCubeB----ReC2------263	-715.9864572	0.02
5	02-TricapCubeB----ReC2------360	-715.9864555	0.02
6	04-Icos-1vx----ReC2------775	-715.9732712	8.29
7	04-Icos-1vx----ReC2------810	-715.9732712	8.29
8	04-Icos-1vx----ReC2------958	-715.9732455	8.31
9	04-Icos-1vx----ReC2------970	-715.9732455	8.31
10	02-TricapCubeA----ReC2------203	-715.9732144	8.33
11	02-TricapCubeB----ReC2------343	-715.9705324	10.01
12	02-TricapCubeB----ReC2------348	-715.9705324	10.01
13	02-TricapCubeB----ReC2------344	-715.9705160	10.02
14	02-TricapCubeA----ReC2------196	-715.9705025	10.03
15	04-Icos-1vx----ReC2------767	-715.9704932	10.03
16	02-TricapCubeB----ReC2------269	-715.9704924	10.03
17	04-Icos-1vx----ReC2------809	-715.9704920	10.04
18	02-TricapCubeB----ReC2------283	-715.9704917	10.04
19	04-Icos-1vx----ReC2------929	-715.9704830	10.04
20	04-Icos-1vx----ReC2------953	-715.9704823	10.04
21	02-TricapCubeB----ReC2------400	-715.9704820	10.04
22	04-Icos-1vx----ReC2------784	-715.9704764	10.05
23	02-TricapCubeB----ReC2------287	-715.9704427	10.07
24	02-TricapCubeB----ReC2------405	-715.9704358	10.07
25	02-TricapCubeB----ReC2------398	-715.9704350	10.07

26	02-TricapCubeA----ReC2------207	-715.9696960	10.53
27	02-TricapCubeA----ReC2------250	-715.9696592	10.56
28	01-PentaCapTrPr----ReC2------53	-715.9696547	10.56
29	01-PentaCapTrPr----ReC2------13	-715.9696526	10.56
30	04-Icos-1vx----ReC2------880	-715.9696478	10.56
31	04-Icos-1vx----ReC2------770	-715.9695698	10.61
32	04-Icos-1vx----ReC2------786	-715.9695698	10.61
33	01-PentaCapTrPr----ReC2------19	-715.9695061	10.65
34	02-TricapCubeB----ReC2------315	-715.9693901	10.73
35	02-TricapCubeB----ReC2------337	-715.9693892	10.73
36	02-TricapCubeB----ReC2------393	-715.9693285	10.77
37	02-TricapCubeA----ReC2------188	-715.9693251	10.77
38	02-TricapCubeB----ReC2------372	-715.9693237	10.77
39	02-TricapCubeB----ReC2------282	-715.9692953	10.79
40	03-OhfusetoPentBipyr----ReC2------556	-715.9683555	11.38
41	03-OhfusetoPentBipyr----ReC2------531	-715.9683536	11.38
42	04-Icos-1vx----ReC2------752	-715.9683035	11.41
43	04-Icos-1vx----ReC2------816	-715.9683035	11.41
44	02-TricapCubeA----ReC2------94	-715.9682864	11.42
45	04-Icos-1vx----ReC2------942	-715.9682417	11.45
46	04-Icos-1vx----ReC2------878	-715.9682139	11.46
47	02-TricapCubeB----ReC2------410	-715.9682052	11.47
48	02-TricapCubeB----ReC2------354	-715.9681961	11.48
49	02-TricapCubeB----ReC2------333	-715.9679190	11.65
50	04-Icos-1vx----ReC2------751	-715.9678659	11.68
51	04-Icos-1vx----ReC2------808	-715.9678651	11.68
52	02-TricapCubeA----ReC2------134	-715.9677020	11.79
53	02-TricapCubeB----ReC2------276	-715.9676989	11.79

54	03-OhfusetoPentBipyr----ReC2------562	-715.9676945	11.79
55	04-Icos-1vx----ReC2------939	-715.9676537	11.82
56	02-TricapCubeB----ReC2------407	-715.9676518	11.82
57	02-TricapCubeB----ReC2------391	-715.9676515	11.82
58	01-PentaCapTrPr----ReC2------83	-715.9676428	11.82
59	02-TricapCubeB----ReC2------392	-715.9676299	11.83
60	02-TricapCubeB----ReC2------261	-715.9676074	11.85
61	01-PentaCapTrPr----ReC2------31	-715.9676068	11.85
62	02-TricapCubeB----ReC2------323_r-28	-715.9676058	11.85
63	02-TricapCubeB----ReC2------365_r-29	-715.9676052	11.85
64	01-PentaCapTrPr----ReC2------7	-715.9676044	11.85
65	02-TricapCubeB----ReC2------351	-715.9676042	11.85
66	02-TricapCubeA----ReC2------199	-715.9676029	11.85
67	02-TricapCubeB----ReC2------335	-715.9675983	11.85
68	04-Icos-1vx----ReC2------943	-715.9675863	11.86
69	02-TricapCubeB----ReC2------336	-715.9675688	11.87
70	02-TricapCubeA----ReC2------118	-715.9674153	11.97
71	01-PentaCapTrPr----ReC2------43	-715.9673468	12.01
72	02-TricapCubeA----ReC2------241_i-29	-715.9669730	12.24
73	02-TricapCubeA----ReC2------214_i-28	-715.9669720	12.24
74	03-OhfusetoPentBipyr----ReC2------561_i-13	-715.9669630	12.25
75	02-TricapCubeB----ReC2------378_r-25	-715.9669496	12.26
76	02-TricapCubeB----ReC2------418	-715.9669298	12.27
77	03-OhfusetoPentBipyr----ReC2------561_r-13	-715.9669232	12.27
78	02-TricapCubeB----ReC2------322	-715.9669048	12.29
79	03-OhfusetoPentBipyr----ReC2------485	-715.9669014	12.29
80	04-Icos-1vx----ReC2------736	-715.9669002	12.29

81	03-OhfusetoPentBipyr----ReC2------564	-715.9668938	12.29
82	01-PentaCapTrPr----ReC2------52	-715.9668895	12.30
83	03-OhfusetoPentBipyr----ReC2------525	-715.9668893	12.30
84	02-TricapCubeA----ReC2------214_r-28	-715.9668823	12.30
85	02-TricapCubeA----ReC2------241_r-29	-715.9668822	12.30
86	02-TricapCubeB----ReC2------421	-715.9668805	12.30
87	04-Icos-1vx----ReC2------797	-715.9668802	12.30
88	04-Icos-1vx----ReC2------820	-715.9668802	12.30
89	02-TricapCubeB----ReC2------379	-715.9668799	12.30
90	04-Icos-1vx----ReC2------763	-715.9668600	12.31
91	04-Icos-1vx----ReC2------793	-715.9668587	12.32
92	03-OhfusetoPentBipyr----ReC2------486	-715.9668349	12.33
93	02-TricapCubeA----ReC2------253	-715.9656962	13.04
94	02-TricapCubeA----ReC2------191	-715.9656950	13.05
95	02-TricapCubeB----ReC2------406	-715.9656660	13.06
96	02-TricapCubeB----ReC2------382	-715.9656628	13.07
97	04-Icos-1vx----ReC2------936	-715.9656524	13.07
98	04-Icos-1vx----ReC2------977	-715.9656524	13.07
99	02-TricapCubeB----ReC2------384	-715.9656515	13.07
100	02-TricapCubeB----ReC2------370	-715.9656440	13.08
101	02-TricapCubeB----ReC2------417	-715.9656440	13.08
102	02-TricapCubeB----ReC2------328	-715.9656423	13.08
103	02-TricapCubeB----ReC2------327	-715.9656395	13.08
104	02-TricapCubeB----ReC2------350	-715.9656385	13.08
105	02-TricapCubeB----ReC2------314	-715.9656118	13.10
106	02-TricapCubeB----ReC2------361	-715.9656118	13.10
107	02-TricapCubeB----ReC2------369	-715.9656085	13.10
108	04-Icos-1vx----ReC2------863	-715.9655941	13.11

109	02-TricapCubeB----ReC2------313	-715.9655905	13.11
110	02-TricapCubeB----ReC2------260	-715.9655859	13.11
111	02-TricapCubeB----ReC2------312	-715.9655834	13.12
112	04-Icos-1vx----ReC2------720	-715.9655782	13.12
113	04-Icos-1vx----ReC2------768	-715.9644482	13.83
114	04-Icos-1vx----ReC2------745	-715.9644410	13.83
115	02-TricapCubeB----ReC2------365_i-29	-715.9641238	14.03
116	02-TricapCubeB----ReC2------323_i-28	-715.9641190	14.03
117	02-TricapCubeB----ReC2------378_i-25	-715.9640434	14.08
118	04-Icos-1vx----ReC2------718	-715.9605172	16.29
119	04-Icos-1vx----ReC2------875	-715.9603768	16.38
120	04-Icos-1vx----ReC2------715	-715.9603265	16.41
121	02-TricapCubeA----ReC2------210	-715.9588482	17.34
122	02-TricapCubeA----ReC2------233	-715.9588482	17.34
123	03-OhfusetoPentBipyr----ReC2------563	-715.9588375	17.35
124	04-Icos-1vx----ReC2------790	-715.9563748	18.89
125	04-Icos-1vx----ReC2------803	-715.9563748	18.89
126	04-Icos-1vx----ReC2------919	-715.9561936	19.01
127	03-OhfusetoPentBipyr----ReC2------577_r-34	-715.9542535	20.23
128	03-OhfusetoPentBipyr----ReC2------569	-715.9541972	20.26
129	03-OhfusetoPentBipyr----ReC2------543	-715.9529786	21.03
130	01-PentaCapTrPr----ReC2------2	-715.9529734	21.03
131	03-OhfusetoPentBipyr----ReC2------686	-715.9527840	21.15
132	03-OhfusetoPentBipyr----ReC2------515	-715.9527691	21.16
133	04-Icos-1vx----ReC2------762	-715.9527545	21.17
134	04-Icos-1vx----ReC2------785	-715.9527545	21.17
135	02-TricapCubeA----ReC2------187	-715.9527511	21.17
136	02-TricapCubeA----ReC2------236	-715.9527511	21.17

137	02-TricapCubeB----ReC2------300	-715.9527434	21.17
138	04-Icos-1vx----ReC2------925	-715.9527373	21.18
139	04-Icos-1vx----ReC2------945	-715.9527053	21.20
140	02-TricapCubeB----ReC2------280	-715.9522821	21.46
141	04-Icos-1vx----ReC2------982	-715.9522656	21.47
142	03-OhfusetoPentBipyr----ReC2------532	-715.9522143	21.50
143	02-TricapCubeA----ReC2------222	-715.9522118	21.51
144	02-TricapCubeA----ReC2------232	-715.9522072	21.51
145	04-Icos-1vx----ReC2------755	-715.9522042	21.51
146	04-Icos-1vx----ReC2------788	-715.9522042	21.51
147	03-OhfusetoPentBipyr----ReC2------497	-715.9520489	21.61
148	01-PentaCapTrPr----ReC2------75	-715.9520434	21.61
149	03-OhfusetoPentBipyr----ReC2------498	-715.9520030	21.64
150	03-OhfusetoPentBipyr----ReC2------511	-715.9520030	21.64
151	03-OhfusetoPentBipyr----ReC2------518	-715.9520019	21.64
152	03-OhfusetoPentBipyr----ReC2------524	-715.9519380	21.68
153	02-TricapCubeA----ReC2------248	-715.9510828	22.21
154	02-TricapCubeA----ReC2------223	-715.9510825	22.22
155	02-TricapCubeA----ReC2------227	-715.9503913	22.65
156	02-TricapCubeA----ReC2------237	-715.9503913	22.65
157	02-TricapCubeB----ReC2------271	-715.9503702	22.66
158	01-PentaCapTrPr----ReC2------14	-715.9503575	22.67
159	01-PentaCapTrPr----ReC2------5	-715.9503575	22.67
160	01-PentaCapTrPr----ReC2------27	-715.9503522	22.67
161	01-PentaCapTrPr----ReC2------47	-715.9503522	22.67
162	02-TricapCubeA----ReC2------242	-715.9502771	22.72
163	02-TricapCubeA----ReC2------206	-715.9502770	22.72
164	02-TricapCubeB----ReC2------275	-715.9502394	22.74

165	04-Icos-1vx----ReC2------744	-715.9498433	22.99
166	01-PentaCapTrPr----ReC2------6	-715.9498241	23.00
167	01-PentaCapTrPr----ReC2------54	-715.9497111	23.08
168	02-TricapCubeB----ReC2------358	-715.9480128	24.14
169	02-TricapCubeB----ReC2------388	-715.9479767	24.16
170	02-TricapCubeB----ReC2------415	-715.9479767	24.16
171	01-PentaCapTrPr----ReC2------28	-715.9479576	24.18
172	03-OhfusetoPentBipyr----ReC2---577_i-34	-715.9479436	24.18
173	03-OhfusetoPentBipyr----ReC2------661	-715.9479014	24.21
174	02-TricapCubeA----ReC2------90	-715.9478999	24.21
175	04-Icos-1vx----ReC2------728	-715.9477112	24.33
176	02-TricapCubeA----ReC2------107	-715.9458850	25.48
177	02-TricapCubeB----ReC2------270	-715.9455155	25.71
178	02-TricapCubeB----ReC2------291	-715.9455155	25.71
179	02-TricapCubeB----ReC2------289	-715.9455054	25.71
180	02-TricapCubeB----ReC2------302	-715.9455054	25.71
181	02-TricapCubeA----ReC2------256	-715.9454876	25.73
182	02-TricapCubeA----ReC2------224	-715.9454869	25.73
183	02-TricapCubeB----ReC2------273	-715.9452906	25.85
184	02-TricapCubeA----ReC2------238	-715.9450250	26.02
185	02-TricapCubeA----ReC2------243	-715.9450250	26.02
186	02-TricapCubeB----ReC2------368	-715.9449468	26.07
187	02-TricapCubeB----ReC2------346	-715.9449213	26.08
188	02-TricapCubeB----ReC2------424	-715.9449181	26.08
189	02-TricapCubeB----ReC2------259	-715.9449165	26.08
190	04-Icos-1vx----ReC2------930	-715.9449103	26.09
191	02-TricapCubeB----ReC2------272	-715.9449019	26.09
192	02-TricapCubeB----ReC2------402	-715.9448763	26.11

193	02-TricapCubeB----ReC2------321_i-11	-715.9443670	26.43
194	02-TricapCubeB----ReC2------317_i-11	-715.9443660	26.43
195	02-TricapCubeB----ReC2------321_r-11	-715.9443303	26.45
196	02-TricapCubeB----ReC2------317_r-11	-715.9443297	26.45
197	02-TricapCubeB----ReC2------373	-715.9442905	26.48
198	02-TricapCubeB----ReC2------377	-715.9442905	26.48
199	03-OhfusetoPentBipyr----ReC2------530	-715.9442664	26.49
200	03-OhfusetoPentBipyr----ReC2------540	-715.9442632	26.49
201	04-Icos-1vx----ReC2------857	-715.9442593	26.50
202	04-Icos-1vx----ReC2------865	-715.9442593	26.50
203	02-TricapCubeA----ReC2------133	-715.9442421	26.51
204	04-Icos-1vx----ReC2------760	-715.9442349	26.51
205	04-Icos-1vx----ReC2------749	-715.9442321	26.51
206	04-Icos-1vx----ReC2------717	-715.9442268	26.52
207	02-TricapCubeB----ReC2------306	-715.9441057	26.59
208	04-Icos-1vx----ReC2------974	-715.9432361	27.14
209	04-Icos-1vx----ReC2------949	-715.9429663	27.31
210	02-TricapCubeB----ReC2------267	-715.9429589	27.31
211	02-TricapCubeB----ReC2------307	-715.9429531	27.32
212	01-PentaCapTrPr----ReC2------45	-715.9429485	27.32
213	01-PentaCapTrPr----ReC2------51	-715.9429479	27.32
214	04-Icos-1vx----ReC2------963	-715.9429439	27.32
215	04-Icos-1vx----ReC2------947	-715.9429330	27.33
216	02-TricapCubeB----ReC2------355	-715.9429300	27.33
217	02-TricapCubeB----ReC2------364	-715.9429253	27.33
218	03-OhfusetoPentBipyr----ReC2------625	-715.9429175	27.34
219	03-OhfusetoPentBipyr----ReC2------635	-715.9429175	27.34
220	03-OhfusetoPentBipyr----ReC2------674	-715.9429156	27.34

221	04-Icos-1vx----ReC2------748	-715.9429084	27.34
222	04-Icos-1vx----ReC2------800	-715.9429084	27.34
223	04-Icos-1vx----ReC2------831	-715.9429052	27.35
224	02-TricapCubeB----ReC2------326	-715.9424865	27.61
225	02-TricapCubeB----ReC2------334	-715.9424863	27.61
226	02-TricapCubeB----ReC2------383	-715.9424577	27.63
227	02-TricapCubeB----ReC2------390	-715.9424577	27.63
228	02-TricapCubeB----ReC2------304	-715.9417998	28.04
229	02-TricapCubeB----ReC2------257	-715.9417992	28.04
230	02-TricapCubeA----ReC2------99	-715.9417834	28.05
231	02-TricapCubeB----ReC2------279	-715.9415343	28.21
232	02-TricapCubeA----ReC2------215	-715.9410999	28.48
233	02-TricapCubeA----ReC2------249	-715.9410995	28.48
234	02-TricapCubeB----ReC2------362	-715.9410653	28.50
235	04-Icos-1vx----ReC2------957	-715.9409720	28.56
236	02-TricapCubeB----ReC2------288	-715.9409712	28.56
237	02-TricapCubeB----ReC2------262	-715.9409602	28.57
238	04-Icos-1vx----ReC2------771	-715.9404538	28.88
239	04-Icos-1vx----ReC2------794	-715.9404538	28.88
240	02-TricapCubeB----ReC2------340	-715.9403468	28.95
241	02-TricapCubeB----ReC2------356	-715.9403457	28.95
242	02-TricapCubeB----ReC2------352	-715.9402058	29.04
243	02-TricapCubeB----ReC2------408	-715.9401728	29.06
244	04-Icos-1vx----ReC2------780	-715.9401680	29.06
245	04-Icos-1vx----ReC2------783	-715.9401480	29.08
246	04-Icos-1vx----ReC2------774	-715.9397342	29.34
247	04-Icos-1vx----ReC2------802	-715.9397342	29.34
248	04-Icos-1vx----ReC2------961	-715.9396239	29.41

249	02-TricapCubeB----ReC2------416	-715.9396144	29.41
250	02-TricapCubeA----ReC2------195	-715.9395988	29.42
251	02-TricapCubeA----ReC2------140	-715.9386708	30.00
252	02-TricapCubeA----ReC2------235	-715.9386159	30.04
253	03-OhfusetoPentBipyr----ReC2------679	-715.9385889	30.06
254	03-OhfusetoPentBipyr----ReC2------697	-715.9385882	30.06
255	04-Icos-1vx----ReC2------867_r-32	-715.9385744	30.06
256	02-TricapCubeB----ReC2------396	-715.9385444	30.08
257	02-TricapCubeB----ReC2------412	-715.9385444	30.08
258	02-TricapCubeB----ReC2------277	-715.9384138	30.16
259	04-Icos-1vx----ReC2------759	-715.9382073	30.29
260	04-Icos-1vx----ReC2------813	-715.9382073	30.29
261	02-TricapCubeB----ReC2------316	-715.9373632	30.82
262	02-TricapCubeB----ReC2------353	-715.9373632	30.82
263	02-TricapCubeB----ReC2------371	-715.9373269	30.85
264	02-TricapCubeB----ReC2------409	-715.9373269	30.85
265	03-OhfusetoPentBipyr----ReC2------613	-715.9369654	31.07
266	03-OhfusetoPentBipyr----ReC2------484	-715.9364877	31.37
267	04-Icos-1vx----ReC2------714	-715.9363779	31.44
268	04-Icos-1vx----ReC2------879	-715.9363740	31.44
269	04-Icos-1vx----ReC2------877	-715.9363674	31.45
270	02-TricapCubeA----ReC2------91	-715.9363456	31.46
271	02-TricapCubeB----ReC2------295	-715.9363432	31.46
272	04-Icos-1vx----ReC2------844	-715.9363337	31.47
273	01-PentaCapTrPr----ReC2------60	-715.9363141	31.48
274	02-TricapCubeB----ReC2------430	-715.9362994	31.49
275	04-Icos-1vx----ReC2------731	-715.9362188	31.54
276	01-PentaCapTrPr----ReC2------22	-715.9361790	31.57

277	01-PentaCapTrPr----ReC2------56	-715.9361747	31.57
278	04-Icos-1vx----ReC2------918	-715.9358594	31.77
279	01-PentaCapTrPr----ReC2------70	-715.9345472	32.59
280	03-OhfusetoPentBipyr----ReC2------575	-715.9345432	32.59
281	03-OhfusetoPentBipyr----ReC2------522	-715.9345352	32.60
282	03-OhfusetoPentBipyr----ReC2------509	-715.9345302	32.60
283	04-Icos-1vx----ReC2------825	-715.9344940	32.62
284	04-Icos-1vx----ReC2------861	-715.9344913	32.63
285	03-OhfusetoPentBipyr----ReC2------560	-715.9344857	32.63
286	03-OhfusetoPentBipyr----ReC2------579	-715.9344849	32.63
287	03-OhfusetoPentBipyr----ReC2------529	-715.9344085	32.68
288	04-Icos-1vx----ReC2------864	-715.9338802	33.01
289	02-TricapCubeA----ReC2------251	-715.9333374	33.35
290	02-TricapCubeA----ReC2------239	-715.9333370	33.35
291	01-PentaCapTrPr----ReC2------21	-715.9333342	33.35
292	01-PentaCapTrPr----ReC2------16	-715.9333281	33.36
293	02-TricapCubeB----ReC2------411	-715.9333233	33.36
294	02-TricapCubeB----ReC2------419	-715.9333233	33.36
295	04-Icos-1vx----ReC2------787	-715.9333105	33.37
296	04-Icos-1vx----ReC2------795	-715.9333105	33.37
297	04-Icos-1vx----ReC2------895	-715.9332937	33.38
298	04-Icos-1vx----ReC2------754	-715.9328955	33.63
299	04-Icos-1vx----ReC2------772	-715.9328955	33.63
300	02-TricapCubeA----ReC2------252	-715.9313384	34.60
301	04-Icos-1vx----ReC2------791	-715.9312591	34.65
302	04-Icos-1vx----ReC2------811	-715.9312591	34.65
303	03-OhfusetoPentBipyr----ReC2------580	-715.9304981	35.13
304	01-PentaCapTrPr----ReC2------12	-715.9304598	35.16

305	03-OhfusetoPentBipyr----ReC2------513	-715.9304041	35.19
306	04-Icos-1vx----ReC2------867_i-32	-715.9303006	35.26
307	04-Icos-1vx----ReC2------779	-715.9300742	35.40
308	02-TricapCubeB----ReC2------342	-715.9285139	36.38
309	02-TricapCubeB----ReC2------332	-715.9285113	36.38
310	01-PentaCapTrPr----ReC2------18	-715.9284845	36.40
311	02-TricapCubeA----ReC2------244	-715.9284379	36.43
312	04-Icos-1vx----ReC2------807	-715.9284140	36.44
313	04-Icos-1vx----ReC2------815	-715.9284140	36.44
314	03-OhfusetoPentBipyr----ReC2------690	-715.9270545	37.29
315	02-TricapCubeB----ReC2------285	-715.9270543	37.29
316	02-TricapCubeB----ReC2------278	-715.9270285	37.31
317	04-Icos-1vx----ReC2------874	-715.9268287	37.43
318	02-TricapCubeA----ReC2------212	-715.9267891	37.46
319	02-TricapCubeA----ReC2------185	-715.9267890	37.46
320	02-TricapCubeB----ReC2------268	-715.9267426	37.49
321	04-Icos-1vx----ReC2------733	-715.9267265	37.50
322	04-Icos-1vx----ReC2------756	-715.9267028	37.51
323	04-Icos-1vx----ReC2------796	-715.9267028	37.51
324	02-TricapCubeA----ReC2------255	-715.9266980	37.52
325	02-TricapCubeB----ReC2------366	-715.9266921	37.52
326	02-TricapCubeA----ReC2------247	-715.9266907	37.52
327	02-TricapCubeB----ReC2------331	-715.9266905	37.52
328	02-TricapCubeB----ReC2------387	-715.9266687	37.54
329	02-TricapCubeB----ReC2------423	-715.9266651	37.54
330	02-TricapCubeA----ReC2------234	-715.9261273	37.87
331	02-TricapCubeA----ReC2------202	-715.9261236	37.88
332	04-Icos-1vx----ReC2------934	-715.9260936	37.90

333	04-Icos-1vx----ReC2------969	-715.9260936	37.90
334	02-TricapCubeB----ReC2------359	-715.9260924	37.90
335	02-TricapCubeB----ReC2------349	-715.9260877	37.90
336	02-TricapCubeB----ReC2------404	-715.9260540	37.92
337	02-TricapCubeB----ReC2------414	-715.9260507	37.92
338	03-OhfusetoPentBipyr----ReC2------572	-715.9253392	38.37
339	02-TricapCubeA----ReC2------220	-715.9248077	38.70
340	02-TricapCubeB----ReC2------381	-715.9248045	38.71
341	02-TricapCubeB----ReC2------397	-715.9248045	38.71
342	04-Icos-1vx----ReC2------960	-715.9247738	38.72
343	04-Icos-1vx----ReC2------978	-715.9247738	38.72
344	03-OhfusetoPentBipyr----ReC2------545	-715.9247613	38.73
345	02-TricapCubeB----ReC2------324	-715.9247530	38.74
346	02-TricapCubeB----ReC2------341	-715.9247521	38.74
347	02-TricapCubeB----ReC2------284	-715.9247483	38.74
348	02-TricapCubeB----ReC2------309	-715.9247474	38.74
349	03-OhfusetoPentBipyr----ReC2------510	-715.9236270	39.44
350	03-OhfusetoPentBipyr----ReC2------521	-715.9235206	39.51
351	02-TricapCubeB----ReC2------258	-715.9233159	39.64
352	02-TricapCubeB----ReC2------320	-715.9233120	39.64
353	02-TricapCubeB----ReC2------374	-715.9232782	39.66
354	02-TricapCubeB----ReC2------401	-715.9232772	39.66
355	02-TricapCubeB----ReC2------319	-715.9232401	39.69
356	02-TricapCubeB----ReC2------345	-715.9232401	39.69
357	04-Icos-1vx----ReC2------859	-715.9232210	39.70
358	04-Icos-1vx----ReC2------950	-715.9232161	39.70
359	04-Icos-1vx----ReC2------971	-715.9232161	39.70
360	04-Icos-1vx----ReC2------830	-715.9232116	39.70

361	04-Icos-1vx----ReC2------846	-715.9232065	39.71
362	02-TricapCubeB----ReC2------376	-715.9232022	39.71
363	02-TricapCubeA----ReC2------108	-715.9231741	39.73
364	01-PentaCapTrPr----ReC2------87	-715.9229784	39.85
365	02-TricapCubeA----ReC2------131	-715.9229527	39.87
366	02-TricapCubeA----ReC2------178	-715.9229522	39.87
367	03-OhfusetoPentBipyr----ReC2------578	-715.9227205	40.01
368	02-TricapCubeA----ReC2------190	-715.9225863	40.10
369	02-TricapCubeA----ReC2------245	-715.9225845	40.10
370	03-OhfusetoPentBipyr----ReC2------600	-715.9223697	40.23
371	03-OhfusetoPentBipyr----ReC2------616	-715.9223681	40.23
372	04-Icos-1vx----ReC2------833	-715.9223005	40.28
373	01-PentaCapTrPr----ReC2------9	-715.9213584	40.87
374	01-PentaCapTrPr----ReC2------36	-715.9213575	40.87
375	03-OhfusetoPentBipyr----ReC2------587	-715.9207873	41.23
376	03-OhfusetoPentBipyr----ReC2------621	-715.9207873	41.23
377	04-Icos-1vx----ReC2------753	-715.9207741	41.23
378	04-Icos-1vx----ReC2------764	-715.9207741	41.23
379	01-PentaCapTrPr----ReC2------37	-715.9206120	41.34
380	01-PentaCapTrPr----ReC2------49	-715.9206120	41.34
381	02-TricapCubeA----ReC2------217	-715.9205617	41.37
382	02-TricapCubeA----ReC2------216	-715.9205574	41.37
383	04-Icos-1vx----ReC2------716	-715.9203629	41.49
384	04-Icos-1vx----ReC2------738	-715.9203629	41.49
385	03-OhfusetoPentBipyr----ReC2------559	-715.9203601	41.49
386	03-OhfusetoPentBipyr----ReC2------544	-715.9203084	41.53
387	01-PentaCapTrPr----ReC2------82	-715.9200063	41.72
388	02-TricapCubeA----ReC2------100	-715.9195884	41.98

389	04-Icos-1vx----ReC2------750	-715.9195747	41.99
390	04-Icos-1vx----ReC2------824	-715.9195747	41.99
391	02-TricapCubeB----ReC2------446	-715.9195395	42.01
392	02-TricapCubeB----ReC2------330	-715.9191730	42.24
393	02-TricapCubeB----ReC2------363	-715.9190782	42.30
394	02-TricapCubeB----ReC2------339	-715.9190773	42.30
395	04-Icos-1vx----ReC2------798	-715.9189346	42.39
396	04-Icos-1vx----ReC2------804	-715.9189346	42.39
397	03-OhfusetoPentBipyr----ReC2------542	-715.9186631	42.56
398	03-OhfusetoPentBipyr----ReC2------570	-715.9186631	42.56
399	01-PentaCapTrPr----ReC2------58	-715.9186305	42.58
400	02-TricapCubeA----ReC2------161	-715.9186221	42.58
401	03-OhfusetoPentBipyr----ReC2------489	-715.9185107	42.65
402	03-OhfusetoPentBipyr----ReC2------490	-715.9184763	42.68
403	03-OhfusetoPentBipyr----ReC2------624	-715.9183013	42.79
404	03-OhfusetoPentBipyr----ReC2------611	-715.9183000	42.79
405	02-TricapCubeA----ReC2------130	-715.9182886	42.79
406	02-TricapCubeA----ReC2------138	-715.9182869	42.79
407	02-TricapCubeB----ReC2------471	-715.9182708	42.81
408	02-TricapCubeA----ReC2------219	-715.9181614	42.87
409	02-TricapCubeA----ReC2------240	-715.9181609	42.87
410	01-PentaCapTrPr----ReC2------1	-715.9181147	42.90
411	01-PentaCapTrPr----ReC2------38	-715.9181121	42.90
412	03-OhfusetoPentBipyr----ReC2------582	-715.9180699	42.93
413	03-OhfusetoPentBipyr----ReC2------552	-715.9180687	42.93
414	04-Icos-1vx----ReC2------852	-715.9180429	42.95
415	01-PentaCapTrPr----ReC2------84	-715.9180299	42.96
416	02-TricapCubeA----ReC2------201	-715.9179977	42.98

417	02-TricapCubeA----ReC2------209	-715.9179977	42.98
418	04-Icos-1vx----ReC2------832	-715.9179920	42.98
419	04-Icos-1vx----ReC2------782	-715.9179811	42.99
420	04-Icos-1vx----ReC2------940	-715.9178118	43.09
421	04-Icos-1vx----ReC2------777	-715.9172991	43.41
422	04-Icos-1vx----ReC2------956	-715.9170116	43.60
423	02-TricapCubeB----ReC2------301	-715.9169835	43.61
424	04-Icos-1vx----ReC2------839	-715.9169524	43.63
425	02-TricapCubeA----ReC2------96	-715.9164576	43.94
426	04-Icos-1vx----ReC2------781	-715.9164412	43.95
427	04-Icos-1vx----ReC2------873	-715.9163806	43.99
428	04-Icos-1vx----ReC2------836	-715.9156717	44.44
429	02-TricapCubeA----ReC2------150	-715.9156380	44.46
430	02-TricapCubeA----ReC2------166	-715.9156378	44.46
431	02-TricapCubeA----ReC2------169	-715.9156347	44.46
432	02-TricapCubeA----ReC2------119	-715.9155857	44.49
433	03-OhfusetoPentBipyr----ReC2------473	-715.9153457	44.64
434	03-OhfusetoPentBipyr----ReC2------500	-715.9153422	44.64
435	04-Icos-1vx----ReC2------730	-715.9153395	44.64
436	02-TricapCubeA----ReC2------95	-715.9153091	44.66
437	03-OhfusetoPentBipyr----ReC2------646	-715.9152859	44.68
438	03-OhfusetoPentBipyr----ReC2------602	-715.9152803	44.68
439	03-OhfusetoPentBipyr----ReC2------691	-715.9152792	44.68
440	03-OhfusetoPentBipyr----ReC2------651	-715.9152781	44.68
441	01-PentaCapTrPr----ReC2------68	-715.9152721	44.69
442	03-OhfusetoPentBipyr----ReC2------519	-715.9152717	44.69
443	03-OhfusetoPentBipyr----ReC2------499	-715.9152679	44.69
444	02-TricapCubeB----ReC2------425	-715.9152141	44.72

445	02-TricapCubeB----ReC2------437	-715.9152141	44.72
446	01-PentaCapTrPr----ReC2------85	-715.9152138	44.72
447	02-TricapCubeA----ReC2------173	-715.9151895	44.74
448	02-TricapCubeB----ReC2------436	-715.9149165	44.91
449	02-TricapCubeB----ReC2------457	-715.9149165	44.91
450	04-Icos-1vx----ReC2------866	-715.9149139	44.91
451	02-TricapCubeA----ReC2------103	-715.9149129	44.91
452	03-OhfusetoPentBipyr----ReC2------609	-715.9148974	44.92
453	03-OhfusetoPentBipyr----ReC2------640	-715.9148953	44.92
454	04-Icos-1vx----ReC2------944	-715.9148810	44.93
455	02-TricapCubeA----ReC2------167	-715.9148706	44.94
456	02-TricapCubeA----ReC2------151	-715.9148634	44.94
457	02-TricapCubeA----ReC2------153	-715.9148616	44.94
458	04-Icos-1vx----ReC2------799	-715.9147166	45.04
459	04-Icos-1vx----ReC2------812	-715.9147166	45.04
460	02-TricapCubeB----ReC2------281	-715.9145447	45.14
461	04-Icos-1vx----ReC2------902	-715.9144757	45.19
462	04-Icos-1vx----ReC2------915	-715.9144757	45.19
463	04-Icos-1vx----ReC2------900	-715.9144639	45.19
464	02-TricapCubeA----ReC2------125	-715.9143723	45.25
465	02-TricapCubeB----ReC2------428	-715.9143515	45.26
466	02-TricapCubeB----ReC2------453	-715.9143515	45.26
467	02-TricapCubeB----ReC2------290	-715.9143489	45.27
468	02-TricapCubeB----ReC2------308	-715.9143438	45.27
469	02-TricapCubeA----ReC2------112	-715.9143204	45.28
470	02-TricapCubeA----ReC2------189	-715.9141182	45.41
471	02-TricapCubeA----ReC2------229	-715.9141182	45.41
472	04-Icos-1vx----ReC2------734	-715.9140761	45.44

473	02-TricapCubeB----ReC2------305	-715.9137946	45.61
474	04-Icos-1vx----ReC2------723	-715.9137479	45.64
475	04-Icos-1vx----ReC2------828	-715.9137042	45.67
476	02-TricapCubeB----ReC2------426	-715.9136609	45.70
477	03-OhfusetoPentBipyr----ReC2------620	-715.9135260	45.78
478	02-TricapCubeB----ReC2------431	-715.9134226	45.85
479	02-TricapCubeB----ReC2------470	-715.9134226	45.85
480	03-OhfusetoPentBipyr----ReC2------696	-715.9133936	45.87
481	03-OhfusetoPentBipyr----ReC2------684	-715.9133913	45.87
482	04-Icos-1vx----ReC2------851	-715.9133778	45.88
483	02-TricapCubeA----ReC2------170	-715.9133648	45.88
484	02-TricapCubeA----ReC2------111	-715.9133569	45.89
485	02-TricapCubeA----ReC2------213	-715.9133169	45.91
486	02-TricapCubeA----ReC2------225	-715.9133169	45.91
487	02-TricapCubeA----ReC2------179	-715.9131959	45.99
488	04-Icos-1vx----ReC2------840	-715.9131953	45.99
489	02-TricapCubeA----ReC2------139	-715.9131952	45.99
490	03-OhfusetoPentBipyr----ReC2------576	-715.9131624	46.01
491	03-OhfusetoPentBipyr----ReC2------583	-715.9131609	46.01
492	03-OhfusetoPentBipyr----ReC2------514	-715.9131051	46.05
493	03-OhfusetoPentBipyr----ReC2------506	-715.9131023	46.05
494	02-TricapCubeA----ReC2------104	-715.9130947	46.05
495	04-Icos-1vx----ReC2------739	-715.9130746	46.07
496	01-PentaCapTrPr----ReC2------57	-715.9130609	46.07
497	01-PentaCapTrPr----ReC2------81	-715.9130609	46.07
498	02-TricapCubeB----ReC2------432	-715.9130147	46.10
499	04-Icos-1vx----ReC2------823	-715.9129978	46.11
500	04-Icos-1vx----ReC2------757	-715.9128458	46.21

501	04-Icos-1vx----ReC2------821	-715.9128458	46.21
502	04-Icos-1vx----ReC2------896	-715.9125859	46.37
503	04-Icos-1vx----ReC2------922	-715.9125859	46.37
504	04-Icos-1vx----ReC2------941	-715.9125732	46.38
505	04-Icos-1vx----ReC2------872	-715.9125726	46.38
506	04-Icos-1vx----ReC2------761	-715.9122621	46.58
507	04-Icos-1vx----ReC2------769	-715.9122621	46.58
508	01-PentaCapTrPr----ReC2------69	-715.9119353	46.78
509	02-TricapCubeA----ReC2------89	-715.9119125	46.80
510	01-PentaCapTrPr----ReC2------4	-715.9118983	46.80
511	03-OhfusetoPentBipyr----ReC2------505	-715.9118118	46.86
512	04-Icos-1vx----ReC2------871_i-17	-715.9117592	46.89
513	04-Icos-1vx----ReC2------871_r-17	-715.9116413	46.97
514	04-Icos-1vx----ReC2------843	-715.9113643	47.14
515	02-TricapCubeB----ReC2------462	-715.9113207	47.17
516	04-Icos-1vx----ReC2------948	-715.9112967	47.18
517	03-OhfusetoPentBipyr----ReC2------547	-715.9112722	47.20
518	03-OhfusetoPentBipyr----ReC2------557	-715.9112717	47.20
519	03-OhfusetoPentBipyr----ReC2------555	-715.9112659	47.20
520	04-Icos-1vx----ReC2------892	-715.9112327	47.22
521	04-Icos-1vx----ReC2------894	-715.9111891	47.25
522	04-Icos-1vx----ReC2------914	-715.9111891	47.25
523	04-Icos-1vx----ReC2------906	-715.9102528	47.84
524	04-Icos-1vx----ReC2------891	-715.9102484	47.84
525	04-Icos-1vx----ReC2------893	-715.9102307	47.85
526	04-Icos-1vx----ReC2------911	-715.9102264	47.85
527	02-TricapCubeA----ReC2------157	-715.9100221	47.98
528	02-TricapCubeB----ReC2------443	-715.9100179	47.98

529	02-TricapCubeB----ReC2------451	-715.9100179	47.98
530	04-Icos-1vx----ReC2------819	-715.9099742	48.01
531	04-Icos-1vx----ReC2------789	-715.9099732	48.01
532	04-Icos-1vx----ReC2------928	-715.9098968	48.06
533	02-TricapCubeA----ReC2------211	-715.9095218	48.30
534	02-TricapCubeB----ReC2------380	-715.9094958	48.31
535	02-TricapCubeB----ReC2------413	-715.9094952	48.31
536	02-TricapCubeB----ReC2------325	-715.9094723	48.33
537	02-TricapCubeB----ReC2------357	-715.9094711	48.33
538	02-TricapCubeA----ReC2------218	-715.9094656	48.33
539	02-TricapCubeA----ReC2------208	-715.9094624	48.33
540	02-TricapCubeB----ReC2------389	-715.9093567	48.40
541	02-TricapCubeB----ReC2------399	-715.9093540	48.40
542	04-Icos-1vx----ReC2------773	-715.9093015	48.43
543	04-Icos-1vx----ReC2------818	-715.9093015	48.43
544	04-Icos-1vx----ReC2------937	-715.9092202	48.48
545	02-TricapCubeB----ReC2------422	-715.9091836	48.51
546	02-TricapCubeB----ReC2------403	-715.9091823	48.51
547	02-TricapCubeB----ReC2------274	-715.9091736	48.51
548	02-TricapCubeB----ReC2------347	-715.9091587	48.52
549	02-TricapCubeB----ReC2------367	-715.9091587	48.52
550	02-TricapCubeA----ReC2------171	-715.9091098	48.55
551	02-TricapCubeA----ReC2------142	-715.9091096	48.55
552	04-Icos-1vx----ReC2------778	-715.9089933	48.63
553	02-TricapCubeA----ReC2------115	-715.9088407	48.72
554	02-TricapCubeA----ReC2------192	-715.9085940	48.88
555	02-TricapCubeA----ReC2------221	-715.9085940	48.88
556	02-TricapCubeB----ReC2------329	-715.9085893	48.88

557	02-TricapCubeB----ReC2------318	-715.9085864	48.88
558	02-TricapCubeB----ReC2------375	-715.9085130	48.93
559	02-TricapCubeB----ReC2------385	-715.9085130	48.93
560	04-Icos-1vx----ReC2------908	-715.9081219	49.17
561	04-Icos-1vx----ReC2------935	-715.9081206	49.17
562	04-Icos-1vx----ReC2------917	-715.9080157	49.24
563	02-TricapCubeA----ReC2------156	-715.9075599	49.53
564	02-TricapCubeA----ReC2------183	-715.9075546	49.53
565	04-Icos-1vx----ReC2------814	-715.9072796	49.70
566	04-Icos-1vx----ReC2------849	-715.9059069	50.56
567	04-Icos-1vx----ReC2------860	-715.9058650	50.59
568	02-TricapCubeA----ReC2------105	-715.9058593	50.59
569	02-TricapCubeB----ReC2------311_i-25	-715.9053974	50.88
570	02-TricapCubeB----ReC2------311_r-25	-715.9053502	50.91
571	02-TricapCubeB----ReC2------303	-715.9052142	51.00
572	04-Icos-1vx----ReC2------899_i-18	-715.9051284	51.05
573	02-TricapCubeB----ReC2------394	-715.9051254	51.05
574	02-TricapCubeB----ReC2------395_i-12	-715.9050994	51.07
575	02-TricapCubeB----ReC2------420_i-12	-715.9050994	51.07
576	04-Icos-1vx----ReC2------907	-715.9050746	51.09
577	02-TricapCubeB----ReC2------338	-715.9050605	51.09
578	04-Icos-1vx----ReC2------899_r-18	-715.9050556	51.10
579	04-Icos-1vx----ReC2------938	-715.9050508	51.10
580	02-TricapCubeB----ReC2------395_r-12	-715.9050178	51.12
581	02-TricapCubeB----ReC2------420_r-12	-715.9050178	51.12
582	04-Icos-1vx----ReC2------901	-715.9050034	51.13
583	03-OhfusetoPentBipyr----ReC2------664	-715.9047493	51.29
584	03-OhfusetoPentBipyr----ReC2------682	-715.9047491	51.29

585	01-PentaCapTrPr----ReC2------77	-715.9029763	52.40
586	02-TricapCubeB----ReC2------266	-715.9027293	52.56
587	02-TricapCubeB----ReC2------297	-715.9026557	52.60
588	03-OhfusetoPentBipyr----ReC2------538	-715.9026458	52.61
589	03-OhfusetoPentBipyr----ReC2------554	-715.9026424	52.61
590	03-OhfusetoPentBipyr----ReC2------526	-715.9019485	53.05
591	03-OhfusetoPentBipyr----ReC2------553	-715.9010348	53.62
592	02-TricapCubeA----ReC2------197	-715.9007555	53.80
593	03-OhfusetoPentBipyr----ReC2------539	-715.9005897	53.90
594	01-PentaCapTrPr----ReC2------35	-715.8993676	54.67
595	03-OhfusetoPentBipyr----ReC2------487	-715.8992896	54.72
596	03-OhfusetoPentBipyr----ReC2------501	-715.8992815	54.72
597	04-Icos-1vx----ReC2------826	-715.8990655	54.86
598	04-Icos-1vx----ReC2------869	-715.8990637	54.86
599	01-PentaCapTrPr----ReC2------78	-715.8988209	55.01
600	02-TricapCubeA----ReC2------143	-715.8988126	55.02
601	02-TricapCubeA----ReC2------193	-715.8987930	55.03
602	02-TricapCubeA----ReC2------230	-715.8986868	55.09
603	02-TricapCubeA----ReC2------246	-715.8986868	55.09
604	04-Icos-1vx----ReC2------841	-715.8986457	55.12
605	04-Icos-1vx----ReC2------862	-715.8986434	55.12
606	04-Icos-1vx----ReC2------719	-715.8986253	55.13
607	04-Icos-1vx----ReC2------722	-715.8986253	55.13
608	03-OhfusetoPentBipyr----ReC2------565	-715.8985548	55.18
609	01-PentaCapTrPr----ReC2------23	-715.8974908	55.84
610	01-PentaCapTrPr----ReC2------8	-715.8974908	55.84
611	02-TricapCubeA----ReC2------160	-715.8974279	55.88
612	02-TricapCubeA----ReC2------128	-715.8974204	55.89

613	04-Icos-1vx----ReC2------806	-715.8971550	56.06
614	04-Icos-1vx----ReC2------822	-715.8971550	56.06
615	02-TricapCubeA----ReC2------136	-715.8971449	56.06
616	02-TricapCubeA----ReC2------122	-715.8971421	56.06
617	02-TricapCubeB----ReC2------450	-715.8970949	56.09
618	04-Icos-1vx----ReC2------726	-715.8970822	56.10
619	03-OhfusetoPentBipyr----ReC2------592	-715.8970736	56.11
620	03-OhfusetoPentBipyr----ReC2------614	-715.8970600	56.12
621	03-OhfusetoPentBipyr----ReC2------517	-715.8968165	56.27
622	03-OhfusetoPentBipyr----ReC2------523	-715.8968140	56.27
623	01-PentaCapTrPr----ReC2------67	-715.8967762	56.29
624	01-PentaCapTrPr----ReC2------15	-715.8967421	56.31
625	01-PentaCapTrPr----ReC2------29	-715.8967421	56.31
626	02-TricapCubeA----ReC2------181	-715.8962835	56.60
627	04-Icos-1vx----ReC2------741	-715.8961434	56.69
628	02-TricapCubeB----ReC2------456	-715.8961072	56.71
629	02-TricapCubeA----ReC2------120	-715.8959469	56.81
630	02-TricapCubeB----ReC2------454	-715.8958610	56.87
631	04-Icos-1vx----ReC2------747	-715.8958225	56.89
632	04-Icos-1vx----ReC2------792	-715.8958221	56.89
633	02-TricapCubeA----ReC2------231	-715.8958202	56.89
634	02-TricapCubeA----ReC2------254	-715.8958202	56.89
635	02-TricapCubeA----ReC2------97	-715.8954522	57.12
636	04-Icos-1vx----ReC2------724	-715.8954336	57.14
637	02-TricapCubeB----ReC2------442	-715.8954171	57.15
638	04-Icos-1vx----ReC2------909	-715.8951312	57.33
639	02-TricapCubeB----ReC2------386	-715.8951015	57.34
640	02-TricapCubeA----ReC2------141	-715.8949163	57.46

641	04-Icos-1vx----ReC2------727	-715.8949117	57.46
642	02-TricapCubeB----ReC2------466	-715.8948893	57.48
643	04-Icos-1vx----ReC2------713	-715.8948192	57.52
644	04-Icos-1vx----ReC2------729	-715.8948192	57.52
645	04-Icos-1vx----ReC2------910	-715.8948053	57.53
646	04-Icos-1vx----ReC2------916	-715.8948053	57.53
647	02-TricapCubeA----ReC2------154	-715.8948030	57.53
648	02-TricapCubeA----ReC2------135	-715.8947982	57.53
649	04-Icos-1vx----ReC2------842	-715.8947926	57.54
650	04-Icos-1vx----ReC2------870	-715.8947926	57.54
651	03-OhfusetoPentBipyr----ReC2------641	-715.8947864	57.54
652	03-OhfusetoPentBipyr----ReC2------633	-715.8947849	57.54
653	03-OhfusetoPentBipyr----ReC2------599	-715.8947606	57.56
654	03-OhfusetoPentBipyr----ReC2------607	-715.8947582	57.56
655	02-TricapCubeB----ReC2------438	-715.8947032	57.59
656	03-OhfusetoPentBipyr----ReC2------677	-715.8938354	58.14
657	04-Icos-1vx----ReC2------968	-715.8925232	58.96
658	04-Icos-1vx----ReC2------980	-715.8925232	58.96
659	02-TricapCubeA----ReC2------106	-715.8924763	58.99
660	03-OhfusetoPentBipyr----ReC2------650	-715.8924637	59.00
661	03-OhfusetoPentBipyr----ReC2------658	-715.8924602	59.00
662	04-Icos-1vx----ReC2------837_i-122	-715.8924249	59.02
663	04-Icos-1vx----ReC2------837_r-122	-715.8922275	59.15
664	04-Icos-1vx----ReC2------981	-715.8920063	59.29
665	04-Icos-1vx----ReC2------758	-715.8915555	59.57
666	04-Icos-1vx----ReC2------805	-715.8915555	59.57
667	01-PentaCapTrPr----ReC2------42	-715.8913882	59.67
668	02-TricapCubeB----ReC2------464	-715.8911759	59.81

669	02-TricapCubeB----ReC2------434	-715.8911235	59.84
670	02-TricapCubeB----ReC2------441	-715.8911235	59.84
671	04-Icos-1vx----ReC2------765	-715.8909909	59.92
672	04-Icos-1vx----ReC2------817	-715.8909909	59.92
673	02-TricapCubeB----ReC2------458	-715.8904396	60.27
674	04-Icos-1vx----ReC2------766	-715.8903527	60.32
675	04-Icos-1vx----ReC2------801	-715.8903527	60.32
676	02-TricapCubeA----ReC2------198	-715.8900795	60.50
677	04-Icos-1vx----ReC2------975	-715.8900286	60.53
678	02-TricapCubeA----ReC2------200	-715.8900245	60.53
679	04-Icos-1vx----ReC2------973	-715.8899171	60.60
680	04-Icos-1vx----ReC2------965	-715.8896968	60.74
681	02-TricapCubeB----ReC2------293	-715.8893130	60.98
682	04-Icos-1vx----ReC2------838	-715.8886341	61.40
683	01-PentaCapTrPr----ReC2------72	-715.8885496	61.46
684	01-PentaCapTrPr----ReC2------79	-715.8885496	61.46
685	02-TricapCubeA----ReC2------165	-715.8885234	61.47
686	02-TricapCubeA----ReC2------149	-715.8885226	61.47
687	04-Icos-1vx----ReC2------933	-715.8883230	61.60
688	02-TricapCubeA----ReC2------164	-715.8876294	62.03
689	02-TricapCubeA----ReC2------148	-715.8876232	62.04
690	02-TricapCubeA----ReC2------186	-715.8875884	62.06
691	02-TricapCubeA----ReC2------204	-715.8875884	62.06
692	02-TricapCubeA----ReC2------92	-715.8875396	62.09
693	02-TricapCubeA----ReC2------93	-715.8874883	62.12
694	02-TricapCubeA----ReC2------98	-715.8874883	62.12
695	02-TricapCubeA----ReC2------180	-715.8874869	62.12
696	01-PentaCapTrPr----ReC2------64	-715.8874841	62.12

697	01-PentaCapTrPr----ReC2------73	-715.8874841	62.12
698	02-TricapCubeA----ReC2------116	-715.8874815	62.13
699	02-TricapCubeB----ReC2------427	-715.8874293	62.16
700	02-TricapCubeB----ReC2------445	-715.8874293	62.16
701	02-TricapCubeA----ReC2------109	-715.8873703	62.20
702	04-Icos-1vx----ReC2------912	-715.8873310	62.22
703	04-Icos-1vx----ReC2------924	-715.8873310	62.22
704	02-TricapCubeA----ReC2------205	-715.8872492	62.27
705	02-TricapCubeA----ReC2------226	-715.8872492	62.27
706	04-Icos-1vx----ReC2------827	-715.8869962	62.43
707	04-Icos-1vx----ReC2------858	-715.8866094	62.67
708	03-OhfusetoPentBipyr----ReC2------537	-715.8865886	62.69
709	03-OhfusetoPentBipyr----ReC2------496	-715.8865784	62.69
710	01-PentaCapTrPr----ReC2------76	-715.8865189	62.73
711	02-TricapCubeA----ReC2------228	-715.8862411	62.90
712	03-OhfusetoPentBipyr----ReC2------503	-715.8860182	63.04
713	03-OhfusetoPentBipyr----ReC2------502	-715.8860180	63.04
714	03-OhfusetoPentBipyr----ReC2------527	-715.8860165	63.05
715	02-TricapCubeB----ReC2------292	-715.8858448	63.15
716	02-TricapCubeB----ReC2------310	-715.8858403	63.16
717	02-TricapCubeB----ReC2------429	-715.8858371	63.16
718	02-TricapCubeB----ReC2------461	-715.8858371	63.16
719	02-TricapCubeA----ReC2------113	-715.8855667	63.33
720	04-Icos-1vx----ReC2------854	-715.8853489	63.46
721	04-Icos-1vx----ReC2------845	-715.8853478	63.46
722	04-Icos-1vx----ReC2------984	-715.8853128	63.49
723	04-Icos-1vx----ReC2------856_r-17	-715.8850640	63.64
724	04-Icos-1vx----ReC2------856_i-17	-715.8850449	63.66

725	04-Icos-1vx----ReC2------964	-715.8843793	64.07
726	02-TricapCubeA----ReC2------124	-715.8843358	64.10
727	02-TricapCubeA----ReC2------184	-715.8843355	64.10
728	04-Icos-1vx----ReC2------931	-715.8843288	64.10
729	01-PentaCapTrPr----ReC2------50	-715.8841975	64.19
730	04-Icos-1vx----ReC2------890	-715.8841839	64.20
731	04-Icos-1vx----ReC2------898	-715.8841829	64.20
732	04-Icos-1vx----ReC2------904	-715.8841211	64.23
733	04-Icos-1vx----ReC2------923	-715.8841211	64.23
734	04-Icos-1vx----ReC2------903	-715.8840958	64.25
735	04-Icos-1vx----ReC2------881	-715.8840395	64.29
736	04-Icos-1vx----ReC2------889	-715.8840395	64.29
737	03-OhfusetoPentBipyr----ReC2------665	-715.8838465	64.41
738	03-OhfusetoPentBipyr----ReC2------676	-715.8838465	64.41
739	01-PentaCapTrPr----ReC2------80	-715.8829886	64.95
740	03-OhfusetoPentBipyr----ReC2------675	-715.8827206	65.11
741	03-OhfusetoPentBipyr----ReC2------689	-715.8827187	65.11
742	02-TricapCubeB----ReC2------449	-715.8826450	65.16
743	02-TricapCubeB----ReC2------435	-715.8826389	65.16
744	01-PentaCapTrPr----ReC2------26	-715.8823542	65.34
745	02-TricapCubeB----ReC2------264	-715.8821938	65.44
746	04-Icos-1vx----ReC2------847	-715.8815064	65.88
747	01-PentaCapTrPr----ReC2------48	-715.8810954	66.13
748	02-TricapCubeB----ReC2------452	-715.8796245	67.06
749	02-TricapCubeB----ReC2------460	-715.8796245	67.06
750	02-TricapCubeB----ReC2------294	-715.8796193	67.06
751	02-TricapCubeB----ReC2------286	-715.8796140	67.06
752	02-TricapCubeA----ReC2------121	-715.8796113	67.06

753	02-TricapCubeA----ReC2------152	-715.8796113	67.06
754	02-TricapCubeA----ReC2------168	-715.8796113	67.06
755	03-OhfusetoPentBipyr----ReC2------657	-715.8795774	67.09
756	03-OhfusetoPentBipyr----ReC2------673	-715.8795774	67.09
757	04-Icos-1vx----ReC2------955	-715.8790775	67.40
758	04-Icos-1vx----ReC2------962	-715.8790775	67.40
759	04-Icos-1vx----ReC2------952	-715.8778514	68.17
760	04-Icos-1vx----ReC2------979	-715.8778514	68.17
761	02-TricapCubeA----ReC2------110	-715.8778079	68.20
762	03-OhfusetoPentBipyr----ReC2------660	-715.8778046	68.20
763	03-OhfusetoPentBipyr----ReC2------666	-715.8778031	68.20
764	04-Icos-1vx----ReC2------967	-715.8769960	68.71
765	03-OhfusetoPentBipyr----ReC2------615	-715.8765019	69.02
766	04-Icos-1vx----ReC2------834	-715.8764939	69.02
767	03-OhfusetoPentBipyr----ReC2------474	-715.8759965	69.33
768	03-OhfusetoPentBipyr----ReC2------512	-715.8759965	69.33
769	03-OhfusetoPentBipyr----ReC2------693	-715.8758198	69.44
770	03-OhfusetoPentBipyr----ReC2------603	-715.8757492	69.49
771	03-OhfusetoPentBipyr----ReC2------622	-715.8757492	69.49
772	03-OhfusetoPentBipyr----ReC2------568	-715.8755787	69.60
773	03-OhfusetoPentBipyr----ReC2------692	-715.8755451	69.62
774	01-PentaCapTrPr----ReC2------63	-715.8749654	69.98
775	01-PentaCapTrPr----ReC2------20	-715.8745078	70.27
776	03-OhfusetoPentBipyr----ReC2------481	-715.8744794	70.29
777	03-OhfusetoPentBipyr----ReC2------482	-715.8744506	70.30
778	03-OhfusetoPentBipyr----ReC2------508	-715.8744505	70.30
779	03-OhfusetoPentBipyr----ReC2------567	-715.8743576	70.36
780	01-PentaCapTrPr----ReC2------10	-715.8740311	70.57

781	02-TricapCubeA----ReC2------174	-715.8736778	70.79
782	04-Icos-1vx----ReC2------725	-715.8736513	70.80
783	04-Icos-1vx----ReC2------743	-715.8736513	70.80
784	04-Icos-1vx----ReC2------932	-715.8730057	71.21
785	04-Icos-1vx----ReC2------721	-715.8722920	71.66
786	04-Icos-1vx----ReC2------735	-715.8722920	71.66
787	02-TricapCubeA----ReC2------158	-715.8722832	71.66
788	02-TricapCubeB----ReC2------433	-715.8722273	71.70
789	01-PentaCapTrPr----ReC2------66	-715.8722127	71.71
790	01-PentaCapTrPr----ReC2------86	-715.8722127	71.71
791	02-TricapCubeB----ReC2------472	-715.8716373	72.07
792	04-Icos-1vx----ReC2------742	-715.8716279	72.07
793	02-TricapCubeB----ReC2------455	-715.8715810	72.10
794	02-TricapCubeB----ReC2------468	-715.8715810	72.10
795	02-TricapCubeA----ReC2------123	-715.8715720	72.11
796	02-TricapCubeA----ReC2------144	-715.8715720	72.11
797	03-OhfusetoPentBipyr----ReC2------595	-715.8706078	72.71
798	03-OhfusetoPentBipyr----ReC2------623	-715.8706078	72.71
799	03-OhfusetoPentBipyr----ReC2------659	-715.8701399	73.01
800	03-OhfusetoPentBipyr----ReC2------644	-715.8699644	73.12
801	02-TricapCubeB----ReC2------298	-715.8699513	73.13
802	03-OhfusetoPentBipyr----ReC2------593	-715.8696953	73.29
803	03-OhfusetoPentBipyr----ReC2------639	-715.8696947	73.29
804	04-Icos-1vx----ReC2------966	-715.8695743	73.36
805	04-Icos-1vx----ReC2------972	-715.8695743	73.36
806	02-TricapCubeB----ReC2------265	-715.8695312	73.39
807	04-Icos-1vx----ReC2------951	-715.8693270	73.52
808	02-TricapCubeB----ReC2------444	-715.8693079	73.53

809	02-TricapCubeB----ReC2------459	-715.8693079	73.53
810	02-TricapCubeB----ReC2------440	-715.8688669	73.81
811	04-Icos-1vx----ReC2------732	-715.8688591	73.81
812	04-Icos-1vx----ReC2------737	-715.8688591	73.81
813	02-TricapCubeA----ReC2------114	-715.8684746	74.05
814	04-Icos-1vx----ReC2------868	-715.8682463	74.20
815	04-Icos-1vx----ReC2------850	-715.8682461	74.20
816	02-TricapCubeA----ReC2------101	-715.8682267	74.21
817	02-TricapCubeA----ReC2------194	-715.8677689	74.50
818	04-Icos-1vx----ReC2------959	-715.8676221	74.59
819	02-TricapCubeA----ReC2------129	-715.8675408	74.64
820	04-Icos-1vx----ReC2------740	-715.8672852	74.80
821	02-TricapCubeB----ReC2------448	-715.8672259	74.84
822	03-OhfusetoPentBipyr----ReC2------548	-715.8672246	74.84
823	03-OhfusetoPentBipyr----ReC2------627	-715.8669914	74.98
824	03-OhfusetoPentBipyr----ReC2------619	-715.8669730	75.00
825	03-OhfusetoPentBipyr----ReC2------495	-715.8669705	75.00
826	02-TricapCubeB----ReC2------439	-715.8669664	75.00
827	02-TricapCubeB----ReC2------465	-715.8669664	75.00
828	03-OhfusetoPentBipyr----ReC2------494	-715.8669542	75.01
829	01-PentaCapTrPr----ReC2------30	-715.8668996	75.04
830	01-PentaCapTrPr----ReC2------24	-715.8668780	75.06
831	01-PentaCapTrPr----ReC2------32	-715.8668780	75.06
832	01-PentaCapTrPr----ReC2------55	-715.8667975	75.11
833	03-OhfusetoPentBipyr----ReC2------566	-715.8665798	75.24
834	02-TricapCubeB----ReC2------447	-715.8663135	75.41
835	02-TricapCubeB----ReC2------467	-715.8663135	75.41
836	02-TricapCubeA----ReC2------177	-715.8662130	75.47

837	02-TricapCubeA----ReC2------147	-715.8662096	75.47
838	02-TricapCubeA----ReC2------163	-715.8662082	75.48
839	02-TricapCubeB----ReC2------463	-715.8654905	75.93
840	02-TricapCubeB----ReC2------469	-715.8654905	75.93
841	02-TricapCubeA----ReC2------127	-715.8642400	76.71
842	02-TricapCubeA----ReC2------176	-715.8642375	76.71
843	02-TricapCubeA----ReC2------146	-715.8639959	76.86
844	02-TricapCubeA----ReC2------162	-715.8639959	76.86
845	02-TricapCubeA----ReC2------137	-715.8639935	76.87
846	03-OhfusetoPentBipyr----ReC2------493	-715.8635265	77.16
847	01-PentaCapTrPr----ReC2------59	-715.8632451	77.33
848	04-Icos-1vx----ReC2------848	-715.8630363	77.47
849	04-Icos-1vx----ReC2------946	-715.8630184	77.48
850	04-Icos-1vx----ReC2------954	-715.8630184	77.48
851	04-Icos-1vx----ReC2------887	-715.8615231	78.42
852	04-Icos-1vx----ReC2------888	-715.8614549	78.46
853	04-Icos-1vx----ReC2------921	-715.8614549	78.46
854	01-PentaCapTrPr----ReC2------34	-715.8614081	78.49
855	01-PentaCapTrPr----ReC2------41	-715.8614081	78.49
856	04-Icos-1vx----ReC2------835	-715.8606654	78.95
857	02-TricapCubeA----ReC2------126	-715.8600830	79.32
858	02-TricapCubeA----ReC2------145	-715.8599997	79.37
859	03-OhfusetoPentBipyr----ReC2------668	-715.8597821	79.51
860	03-OhfusetoPentBipyr----ReC2------584	-715.8596368	79.60
861	01-PentaCapTrPr----ReC2------11	-715.8595951	79.63
862	01-PentaCapTrPr----ReC2------44	-715.8595951	79.63
863	03-OhfusetoPentBipyr----ReC2------610	-715.8595521	79.65
864	03-OhfusetoPentBipyr----ReC2------648	-715.8595521	79.65

865	02-TricapCubeA----ReC2------117	-715.8595284	79.67
866	04-Icos-1vx----ReC2------876	-715.8588918	80.07
867	04-Icos-1vx----ReC2------926	-715.8585712	80.27
868	04-Icos-1vx----ReC2------920	-715.8584660	80.33
869	04-Icos-1vx----ReC2------927	-715.8584660	80.33
870	04-Icos-1vx----ReC2------886	-715.8576754	80.83
871	04-Icos-1vx----ReC2------913	-715.8576754	80.83
872	04-Icos-1vx----ReC2------884	-715.8576022	80.88
873	03-OhfusetoPentBipyr----ReC2------479	-715.8562659	81.71
874	03-OhfusetoPentBipyr----ReC2------528	-715.8562000	81.76
875	03-OhfusetoPentBipyr----ReC2------478	-715.8561929	81.76
876	01-PentaCapTrPr----ReC2------61	-715.8558023	82.01
877	02-TricapCubeA----ReC2------132	-715.8557562	82.03
878	04-Icos-1vx----ReC2------829	-715.8556180	82.12
879	04-Icos-1vx----ReC2------853	-715.8556180	82.12
880	03-OhfusetoPentBipyr----ReC2------597	-715.8555997	82.13
881	04-Icos-1vx----ReC2------885	-715.8535452	83.42
882	04-Icos-1vx----ReC2------883	-715.8534721	83.47
883	04-Icos-1vx----ReC2------905	-715.8534721	83.47
884	04-Icos-1vx----ReC2------882	-715.8534673	83.47
885	04-Icos-1vx----ReC2------897	-715.8534673	83.47
886	02-TricapCubeA----ReC2------102	-715.8512327	84.87
887	03-OhfusetoPentBipyr----ReC2------475	-715.8508403	85.12
888	03-OhfusetoPentBipyr----ReC2------520	-715.8508364	85.12
889	03-OhfusetoPentBipyr----ReC2------476	-715.8508023	85.14
890	04-Icos-1vx----ReC2------976	-715.8503600	85.42
891	04-Icos-1vx----ReC2------983	-715.8503600	85.42
892	01-PentaCapTrPr----ReC2------71	-715.8502330	85.50

893	04-Icos-1vx----ReC2------855	-715.8499158	85.70
894	02-TricapCubeA----ReC2------182	-715.8498873	85.72
895	02-TricapCubeA----ReC2------172	-715.8498789	85.72
896	03-OhfusetoPentBipyr----ReC2------581	-715.8491513	86.18
897	03-OhfusetoPentBipyr----ReC2------536	-715.8490849	86.22
898	02-TricapCubeB----ReC2------299	-715.8440597	89.37
899	01-PentaCapTrPr----ReC2------25	-715.8428642	90.12
900	01-PentaCapTrPr----ReC2------39	-715.8428642	90.12
901	03-OhfusetoPentBipyr----ReC2------534	-715.8426667	90.25
902	03-OhfusetoPentBipyr----ReC2------573	-715.8426667	90.25
903	03-OhfusetoPentBipyr----ReC2------535	-715.8416415	90.89
904	03-OhfusetoPentBipyr----ReC2------601	-715.8415126	90.97
905	03-OhfusetoPentBipyr----ReC2------638	-715.8415126	90.97
906	01-PentaCapTrPr----ReC2------62	-715.8413139	91.10
907	01-PentaCapTrPr----ReC2------65	-715.8413139	91.10
908	02-TricapCubeA----ReC2------175	-715.8403862	91.68
909	02-TricapCubeA----ReC2------159	-715.8403786	91.68
910	03-OhfusetoPentBipyr----ReC2------507	-715.8394233	92.28
911	03-OhfusetoPentBipyr----ReC2------550	-715.8381768	93.07
912	03-OhfusetoPentBipyr----ReC2------574	-715.8381768	93.07
913	03-OhfusetoPentBipyr----ReC2------492	-715.8367439	93.96
914	03-OhfusetoPentBipyr----ReC2------491	-715.8367327	93.97
915	03-OhfusetoPentBipyr----ReC2------551	-715.8358629	94.52
916	03-OhfusetoPentBipyr----ReC2------590	-715.8333822	96.07
917	02-TricapCubeA----ReC2------155	-715.8324459	96.66
918	03-OhfusetoPentBipyr----ReC2------618	-715.8324009	96.69
919	03-OhfusetoPentBipyr----ReC2------642	-715.8324009	96.69
920	03-OhfusetoPentBipyr----ReC2------480	-715.8315662	97.21

921	03-OhfusetoPentBipyr----ReC2------504	-715.8315662	97.21
922	03-OhfusetoPentBipyr----ReC2------711	-715.8305277	97.87
923	01-PentaCapTrPr----ReC2------17	-715.8298448	98.29
924	01-PentaCapTrPr----ReC2------40	-715.8298448	98.29
925	03-OhfusetoPentBipyr----ReC2------667	-715.8279100	99.51
926	03-OhfusetoPentBipyr----ReC2------652	-715.8279096	99.51
927	03-OhfusetoPentBipyr----ReC2------680	-715.8271541	99.98
928	03-OhfusetoPentBipyr----ReC2------681	-715.8271541	99.98
929	03-OhfusetoPentBipyr----ReC2------698	-715.8268339	100.18
930	03-OhfusetoPentBipyr----ReC2------663	-715.8268244	100.19
931	01-PentaCapTrPr----ReC2------3	-715.8241362	101.88
932	01-PentaCapTrPr----ReC2------46	-715.8241362	101.88
933	03-OhfusetoPentBipyr----ReC2------647	-715.8184110	105.47
934	03-OhfusetoPentBipyr----ReC2------594	-715.8184101	105.47
935	03-OhfusetoPentBipyr----ReC2------558	-715.8182866	105.55
936	03-OhfusetoPentBipyr----ReC2------571	-715.8182866	105.55
937	01-PentaCapTrPr----ReC2------74	-715.8177885	105.86
938	01-PentaCapTrPr----ReC2------88	-715.8177885	105.86
939	03-OhfusetoPentBipyr----ReC2------628	-715.8169431	106.39
940	03-OhfusetoPentBipyr----ReC2------533	-715.8105783	110.38
941	03-OhfusetoPentBipyr----ReC2------549	-715.8105783	110.38
942	03-OhfusetoPentBipyr----ReC2------702	-715.8080020	112.00
943	01-PentaCapTrPr----ReC2------33	-715.8076211	112.24
944	03-OhfusetoPentBipyr----ReC2------488	-715.8065801	112.89
945	03-OhfusetoPentBipyr----ReC2------477	-715.8065732	112.90
946	03-OhfusetoPentBipyr----ReC2------589	-715.8064475	112.98
947	03-OhfusetoPentBipyr----ReC2------541	-715.8064196	112.99
948	03-OhfusetoPentBipyr----ReC2------546	-715.8064196	112.99

949	03-OhfusetoPentBipyr----ReC2------704	-715.8041647	114.41
950	03-OhfusetoPentBipyr----ReC2------688	-715.8041632	114.41
951	03-OhfusetoPentBipyr----ReC2------671	-715.8033903	114.89
952	03-OhfusetoPentBipyr----ReC2------695	-715.8028443	115.24
953	03-OhfusetoPentBipyr----ReC2------700	-715.7994089	117.39
954	03-OhfusetoPentBipyr----ReC2------678	-715.7970818	118.85
955	03-OhfusetoPentBipyr----ReC2------705	-715.7970817	118.85
956	03-OhfusetoPentBipyr----ReC2------626	-715.7962114	119.40
957	03-OhfusetoPentBipyr----ReC2------643	-715.7962068	119.40
958	03-OhfusetoPentBipyr----ReC2------703	-715.7956930	119.73
959	03-OhfusetoPentBipyr----ReC2------706	-715.7909175	122.72
960	03-OhfusetoPentBipyr----ReC2------662	-715.7909107	122.73
961	03-OhfusetoPentBipyr----ReC2------637	-715.7906773	122.87
962	03-OhfusetoPentBipyr----ReC2------585	-715.7906706	122.88
963	03-OhfusetoPentBipyr----ReC2------701	-715.7893950	123.68
964	03-OhfusetoPentBipyr----ReC2------606	-715.7877359	124.72
965	03-OhfusetoPentBipyr----ReC2------670	-715.7838879	127.13
966	03-OhfusetoPentBipyr----ReC2------709	-715.7838879	127.13
967	03-OhfusetoPentBipyr----ReC2------653	-715.7825026	128.00
968	03-OhfusetoPentBipyr----ReC2------710	-715.7821785	128.21
969	03-OhfusetoPentBipyr----ReC2------617	-715.7795938	129.83
970	03-OhfusetoPentBipyr----ReC2------634	-715.7795938	129.83
971	03-OhfusetoPentBipyr----ReC2------604	-715.7794319	129.93
972	03-OhfusetoPentBipyr----ReC2------630	-715.7794319	129.93
973	03-OhfusetoPentBipyr----ReC2------612	-715.7794022	129.95
974	03-OhfusetoPentBipyr----ReC2------632	-715.7794022	129.95
975	03-OhfusetoPentBipyr----ReC2------654	-715.7789932	130.20
976	03-OhfusetoPentBipyr----ReC2------707	-715.7789932	130.20

977	03-OhfusetoPentBipyr----ReC2------605	-715.7781925	130.71
978	03-OhfusetoPentBipyr----ReC2------687	-715.7763877	131.84
979	03-OhfusetoPentBipyr----ReC2------712	-715.7763753	131.85
980	03-OhfusetoPentBipyr----ReC2------586	-715.7746621	132.92
981	03-OhfusetoPentBipyr----ReC2------645	-715.7746621	132.92
982	03-OhfusetoPentBipyr----ReC2------636	-715.7715187	134.89
983	03-OhfusetoPentBipyr----ReC2------672	-715.7703914	135.60
984	03-OhfusetoPentBipyr----ReC2------685	-715.7703914	135.60
985	03-OhfusetoPentBipyr----ReC2------588	-715.7698165	135.96
986	03-OhfusetoPentBipyr----ReC2------629	-715.7698165	135.96
987	03-OhfusetoPentBipyr----ReC2------591	-715.7691238	136.40
988	03-OhfusetoPentBipyr----ReC2------598	-715.7691238	136.40
989	03-OhfusetoPentBipyr----ReC2------483	-715.7674786	137.43
990	03-OhfusetoPentBipyr----ReC2------516	-715.7674786	137.43
991	03-OhfusetoPentBipyr----ReC2------655	-715.7657359	138.52
992	03-OhfusetoPentBipyr----ReC2------699	-715.7657344	138.52
993	03-OhfusetoPentBipyr----ReC2------596	-715.7641489	139.52
994	03-OhfusetoPentBipyr----ReC2------631	-715.7641489	139.52
995	03-OhfusetoPentBipyr----ReC2------608	-715.7609140	141.55
996	03-OhfusetoPentBipyr----ReC2------669	-715.7476383	149.88
997	03-OhfusetoPentBipyr----ReC2------683	-715.7472127	150.15
998	03-OhfusetoPentBipyr----ReC2------656	-715.7472084	150.15
999	03-OhfusetoPentBipyr----ReC2------694	-715.7462438	150.76
1000	03-OhfusetoPentBipyr----ReC2------708	-715.7462438	150.76
1001	03-OhfusetoPentBipyr----ReC2------649	-715.7196529	167.44

Table 5A. Initial $(CO)_2(NO)ReC_2B_9H_{11}$ structures (one example from each family), a total of 350 structures:

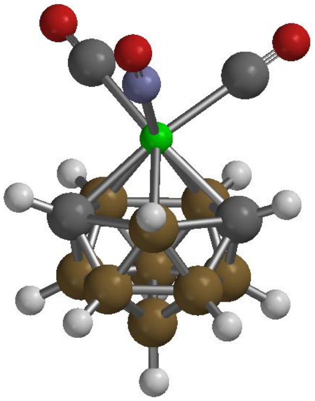<br>1. Icosahedron 30 structures
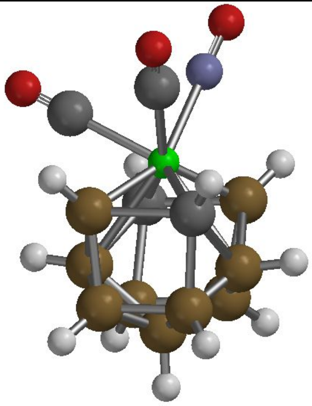<br>2. Cubeoctahedron 150 structures
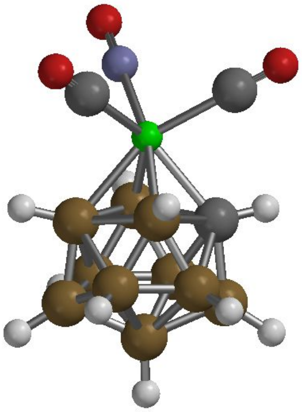<br>3. Anticubeoctahedron 170 structures

Table 5B. Distances table for the lowest-lying $(CO)_2(NO)ReC_2B_9H_{11}$ structures after M06L/6-311G(d,p)//SDD optimization. Included are the ZPcorrected E (a.u.), relative energy (kcal/mol) and symmetry.

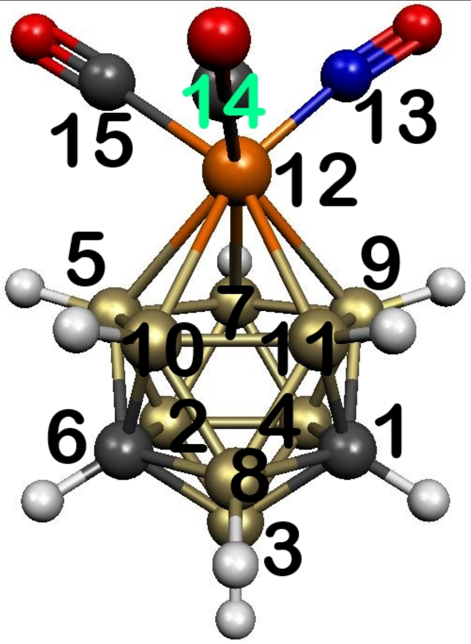

1. -741.69942670 0.0 (C_S)

	1	2	3	4	5
1 C	0.000000				
2 B	2.715735	0.000000			
3 B	1.672388	1.750248	0.000000		
4 B	1.683897	1.761201	1.747891	0.000000	
5 B	3.206950	1.756823	2.838802	2.861643	0.000000
6 C	2.575723	1.690158	1.673305	2.718384	1.686644
7 B	2.747900	1.754377	2.847727	1.760842	1.802905
8 B	1.672947	2.847158	1.764553	2.843192	2.846855
9 B	1.685941	2.859371	2.839835	1.755278	2.895079
10 B	2.740168	2.868806	2.851441	3.380557	1.790965
11 B	1.693673	3.388281	2.860208	2.879516	2.911872
12 Re	3.433773	3.570773	4.142920	3.562972	2.328950
13 N	4.393616	4.865030	5.432789	4.363586	3.961078
14 C	4.542109	5.181794	5.386444	5.219818	3.737323
15 C	5.168881	4.316894	5.425600	4.903162	2.770043

	6	7	8	9	10
6 C	0.000000				
7 B	2.748776	0.000000			
8 B	1.676621	3.375199	0.000000		
9 B	3.211927	1.806063	2.847414	0.000000	
10 B	1.682137	2.931430	1.746604	2.913728	0.000000
11 B	2.744145	2.940959	1.745824	1.806222	1.813275
12 Re	3.450536	2.343771	3.537369	2.307101	2.326253
13 N	5.111493	3.231836	5.002487	2.837665	4.093692
14 C	4.480105	4.263664	4.168958	3.825324	2.853931

15	C	4.343655	3.254445	5.017651	4.065436	3.450055

		11	12	13	14	15
11	B	0.000000				
12	Re	2.309102	0.000000			
13	N	3.458386	1.839560	0.000000		
14	C	2.913473	1.986331	2.854918	0.000000	
15	C	4.175670	1.996545	2.849386	2.871214	0.000000

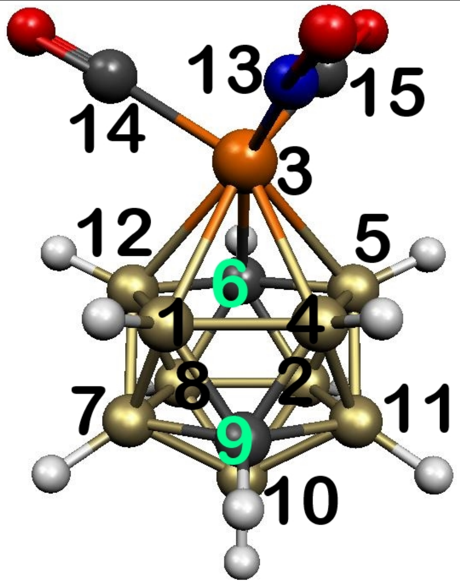

2. -741.69660170 +1.8 (C_S)

		1	2	3	4	5
1	B	0.000000				
2	B	3.380265	0.000000			
3	Re	2.339887	3.586706	0.000000		
4	B	1.811829	2.867097	2.312627	0.000000	
5	B	2.908046	1.770155	2.306610	1.789935	0.000000
6	C	2.784037	1.683918	2.319702	2.782347	1.719538
7	B	1.750633	2.844243	3.580561	2.867535	3.363555
8	B	2.876830	1.754052	3.605502	3.380264	2.865887
9	C	1.681846	2.705779	3.441257	1.679173	2.722124
10	B	2.862501	1.749801	4.176954	2.861221	2.858899
11	B	2.875604	1.752313	3.565867	1.760187	1.762079
12	B	1.787071	2.842808	2.331579	2.884252	2.864009
13	N	3.195068	5.075884	1.823255	2.872145	3.515034
14	C	3.299197	5.033136	1.995472	4.099326	4.147648
15	C	4.274843	4.268857	1.992464	3.797687	2.923368

		6	7	8	9	10
6	C	0.000000				
7	B	2.722900	0.000000			
8	B	1.679514	1.761227	0.000000		
9	C	3.050212	1.684637	2.711725	0.000000	
10	B	2.718721	1.766050	1.749619	1.685243	0.000000

11	B	2.731458	2.850434	2.842917	1.681607	1.760264
12	B	1.689968	1.751186	1.764715	2.710186	2.842490
13	N	4.075653	4.840490	5.263631	4.289433	5.468661
14	C	3.443916	4.345279	4.428415	4.821352	5.458890
15	C	3.031721	5.242029	4.684243	5.090026	5.456768

		11	12	13	14	15
11	B	0.000000				
12	B	3.344425	0.000000			
13	N	4.522952	3.923601	0.000000		
14	C	5.321456	2.785565	2.811089	0.000000	
15	C	4.642275	3.812248	2.810819	2.869263	0.000000

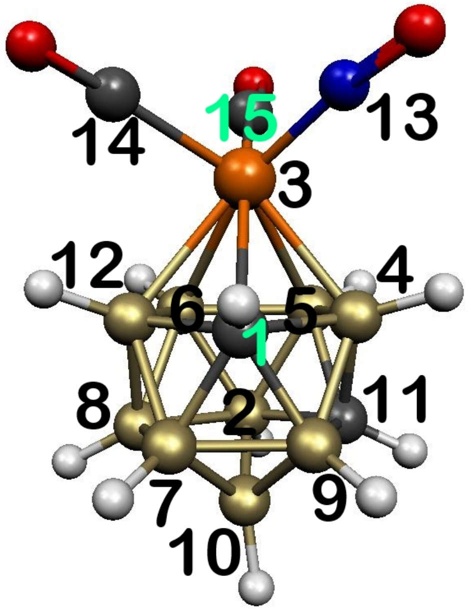

3. -741.69521340 +2.6 C_1

		1	2	3	4	5
1	C	0.000000				
2	B	3.225036	0.000000			
3	Re	2.325325	3.569532	0.000000		
4	B	1.703380	2.842351	2.285921	0.000000	
5	B	2.806704	1.760395	2.317334	1.789818	0.000000
6	B	2.803007	1.760566	2.341631	2.883245	1.807539
7	B	1.680815	2.847667	3.600732	2.845532	3.391406
8	B	2.749569	1.760959	3.599434	3.350714	2.882189
9	B	1.671030	2.834740	3.573776	1.760036	2.874556
10	B	2.732186	1.757010	4.179539	2.850718	2.871251
11	C	2.598430	1.685523	3.437533	1.673891	1.691203
12	B	1.704270	2.836960	2.329930	2.833458	2.882989
13	N	3.243377	5.067330	1.840024	2.861825	3.526282
14	C	3.374820	5.012827	2.001050	4.088454	4.157399
15	C	4.216300	4.236722	1.972696	3.756829	2.903582

		6	7	8	9	10
6	B	0.000000				

7	B	2.881196	0.000000			
8	B	1.769901	1.767471	0.000000		
9	B	3.372586	1.745927	2.839102	0.000000	
10	B	2.864582	1.758469	1.756248	1.755159	0.000000
11	C	2.741144	2.711763	2.720559	1.672450	1.689811
12	B	1.782746	1.760480	1.761679	2.827326	2.838041
13	N	4.126006	4.862969	5.269119	4.373963	5.475991
14	C	3.404721	4.391740	4.419882	4.969806	5.473462
15	C	2.945979	5.245996	4.668774	5.212458	5.440903
		11	12	13	14	15
11	C	0.000000				
12	B	3.175897	0.000000			
13	N	4.428925	3.928741	0.000000		
14	C	5.185170	2.799895	2.853378	0.000000	
15	C	4.527820	3.808562	2.808002	2.835078	0.000000

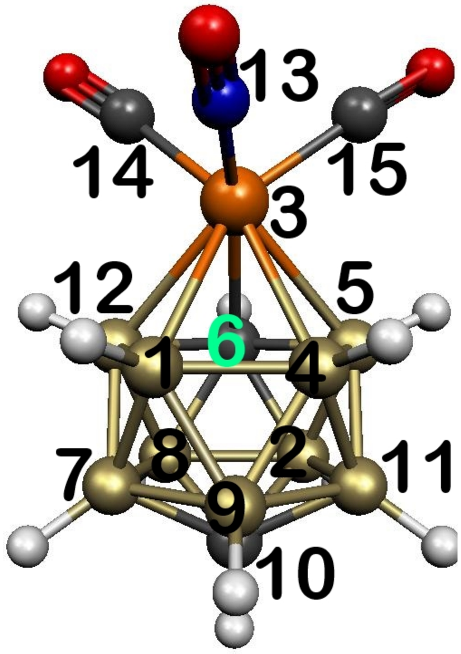

4. -741.69214110 +4.6 (C_S)

		1	2	3	4	5
1	B	0.000000				
2	B	3.369014	0.000000			
3	Re	2.341744	3.570669	0.000000		
4	B	1.810696	2.860222	2.351682	0.000000	
5	B	2.895889	1.755836	2.317401	1.788603	0.000000
6	C	2.803378	1.674039	2.306351	2.803259	1.716653
7	B	1.761739	2.836974	3.577220	2.875009	3.356667
8	B	2.858845	1.751998	3.564641	3.368720	2.846692
9	B	1.751707	2.833763	3.575147	1.752199	2.847426
10	C	2.731137	1.673164	4.019236	2.730235	2.719390
11	B	2.873481	1.750425	3.583016	1.756891	1.756155
12	B	1.791739	2.853342	2.309156	2.904437	2.875276
13	N	2.952242	5.184683	1.822829	3.054933	3.779621

14	C	3.657638	4.788774	1.990855	4.268036	3.957749
15	C	4.215197	4.329096	1.993676	3.511169	2.794854

		6	7	8	9	10
6	C	0.000000				
7	B	2.743512	0.000000			
8	B	1.675835	1.746471	0.000000		
9	B	3.225300	1.761204	2.832111	0.000000	
10	C	2.597899	1.683658	1.673760	1.684582	0.000000
11	B	2.742008	2.853102	2.839535	1.763265	1.686307
12	B	1.725737	1.759579	1.757072	2.851469	2.725122
13	N	4.086835	4.624810	5.126578	4.334985	5.310539
14	C	3.147691	4.534395	4.273056	5.155337	5.311528
15	C	3.279302	5.332014	4.900081	5.073178	5.318754

		11	12	13	14	15
11	B	0.000000				
12	B	3.364503	0.000000			
13	N	4.723667	3.653118	0.000000		
14	C	5.304861	2.832079	2.810156	0.000000	
15	C	4.451563	4.060546	2.808445	2.853884	0.000000

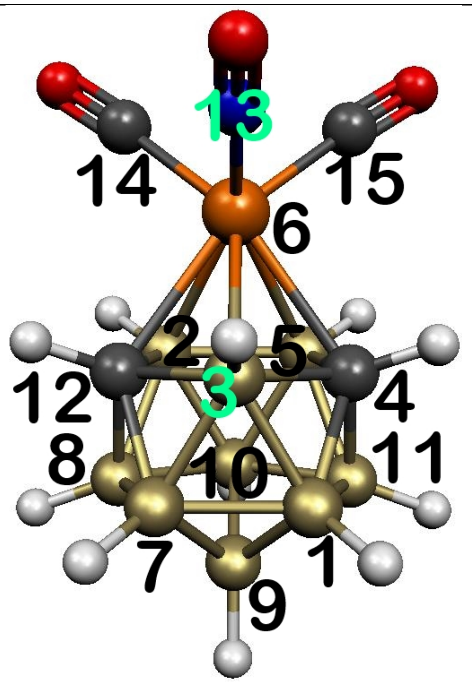

5. -741.68973690 +6.1 C_S

		1	2	3	4	5
1	B	0.000000				
2	B	3.351342	0.000000			
3	B	1.753013	2.813814	0.000000		
4	C	1.684864	2.762999	1.689012	0.000000	
5	B	2.847869	1.772295	2.814459	1.703013	0.000000
6	Re	3.606042	2.321373	2.310838	2.327197	2.320698
7	B	1.755877	2.848726	1.751657	2.743366	3.349323
8	B	2.840922	1.764966	2.817812	3.227034	2.853792
9	B	1.766384	2.858462	2.837034	2.742004	2.856097
10	B	2.851448	1.764505	3.316855	2.743189	1.761859
11	B	1.754567	2.856204	2.819751	1.681517	1.764150
12	C	2.743409	1.702648	1.685439	2.668637	2.761218
13	N	4.471107	4.032045	2.883401	3.401461	4.028002
14	C	5.334935	2.870799	3.964100	4.209749	3.630530
15	C	4.849091	3.627066	3.963582	3.201334	2.864094

		6	7	8	9	10
6	Re	0.000000				
7	B	3.607335	0.000000			
8	B	3.604506	1.754217	0.000000		
9	B	4.211979	1.765827	1.758528	0.000000	
10	B	3.598010	2.850905	1.763899	1.766927	0.000000
11	B	3.603227	2.840126	2.842275	1.757556	1.763473
12	C	2.328361	1.685820	1.682198	2.742945	2.743732
13	N	1.842134	4.476238	4.995931	5.512852	5.287248
14	C	1.977123	4.855762	4.347371	5.513255	4.563613
15	C	1.976765	5.332047	5.174100	5.506183	4.555906

		11	12	13	14	15
11	B	0.000000				

12	C	3.227002	0.000000			
13	N	4.989671	3.409293	0.000000		
14	C	5.177960	3.207750	2.798841	0.000000	
15	C	4.338110	4.208423	2.795291	2.790695	0.000000

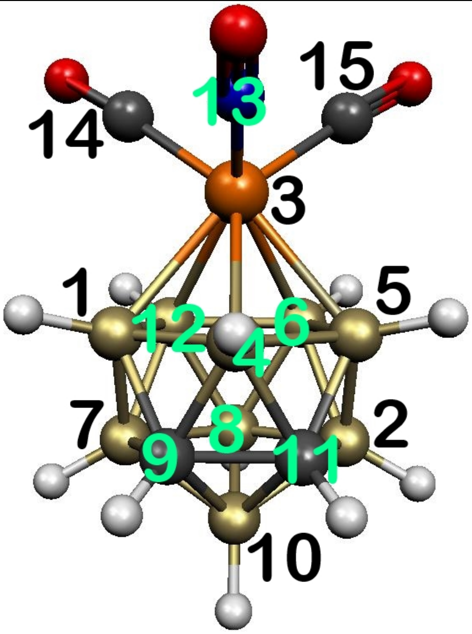

6. -741.67369120 +16.2 C_S

		1	2	3	4	5
1	B	0.000000				
2	B	3.387496	0.000000			
3	Re	2.318518	3.566125	0.000000		
4	B	1.812809	2.869397	2.276454	0.000000	
5	B	2.941404	1.756524	2.317339	1.809588	0.000000
6	B	2.918256	1.757569	2.351927	2.912915	1.813606
7	B	1.756371	2.854018	3.567765	2.871876	3.388912
8	B	2.890825	1.766862	3.608689	3.394201	2.891996
9	C	1.677448	2.718066	3.413419	1.688769	2.745403
10	B	2.868632	1.760744	4.157260	2.872629	2.868781
11	C	2.745786	1.673406	3.412418	1.687690	1.676056
12	B	1.813992	2.858730	2.354615	2.916289	2.921345
13	N	3.355364	4.955800	1.837464	2.807765	3.351080
14	C	3.065604	5.117231	1.995514	3.932471	4.223270
15	C	4.224170	4.236260	1.995194	3.926101	3.056754

		6	7	8	9	10
6	B	0.000000				
7	B	2.857966	0.000000			
8	B	1.768811	1.766840	0.000000		
9	C	3.206721	1.675529	2.717611	0.000000	
10	B	2.847592	1.761522	1.743033	1.694604	0.000000
11	C	2.723885	2.718920	2.716831	1.606986	1.694220
12	B	1.779889	1.758292	1.768690	2.726838	2.848789
13	N	4.065704	4.959103	5.295155	4.326244	5.458011

14	C	3.606768	4.243494	4.519913	4.645640	5.419608
15	C	2.809616	5.118771	4.519223	5.114296	5.415471

		11	12	13	14	15
11	C	0.000000				
12	B	3.208214	0.000000			
13	N	4.324569	4.069547	0.000000		
14	C	5.117020	2.812454	2.851298	0.000000	
15	C	4.637264	3.611716	2.845553	2.879244	0.000000

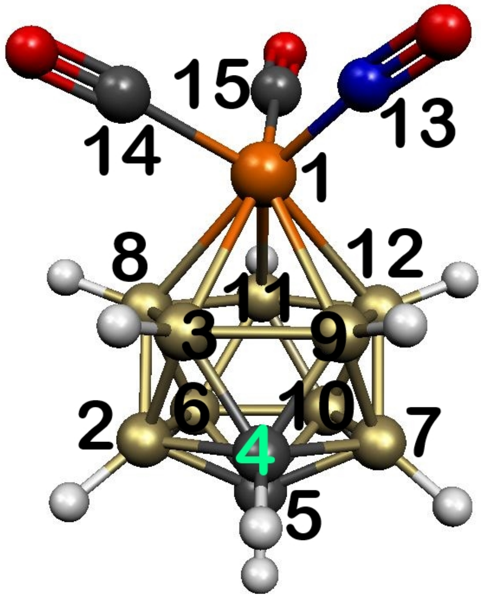

7. -741.67317560 +16.5 (C_s)

		1	2	3	4	5
1	Re	0.000000				
2	B	3.552642	0.000000			
3	B	2.306317	1.757725	0.000000		
4	C	3.416159	1.686407	1.679739	0.000000	
5	C	3.995378	1.692619	2.740544	1.615860	0.000000
6	B	3.583875	1.762013	2.881533	2.719486	1.665136
7	B	3.554946	2.855099	2.877367	1.687435	1.690128
8	B	2.343897	1.751545	1.819212	2.738712	2.721120
9	B	2.302143	2.866223	1.808431	1.673996	2.730488
10	B	3.574035	2.854694	3.388326	2.721269	1.671010
11	B	2.339752	2.857719	2.914501	3.207660	2.708677
12	B	2.348334	3.388887	2.942356	2.739544	2.723178
13	N	1.837826	5.038552	3.491452	4.394780	5.298672
14	C	1.986621	4.187003	2.892054	4.508689	5.252717
15	C	1.996304	5.006572	4.156154	5.153865	5.277121

		6	7	8	9	10
6	B	0.000000				
7	B	2.853502	0.000000			
8	B	1.758634	3.384827	0.000000		
9	B	3.380400	1.754568	2.928491	0.000000	

10	B	1.764722	1.763642	2.870850	2.867432	0.000000
11	B	1.763452	2.864770	1.794701	2.907667	1.759105
12	B	2.884073	1.750238	2.943926	1.810105	1.754063
13	N	5.256084	4.350735	4.121482	2.844665	4.850692
14	C	4.610152	5.205626	2.895973	3.795733	5.202217
15	C	4.404952	4.914421	3.424391	4.079180	4.327229

		11	12	13	14	15
11	B	0.000000				
12	B	1.814093	0.000000			
13	N	3.953455	3.211922	0.000000		
14	C	3.776447	4.271596	2.848419	0.000000	
15	C	2.765016	3.286839	2.845073	2.868752	0.000000

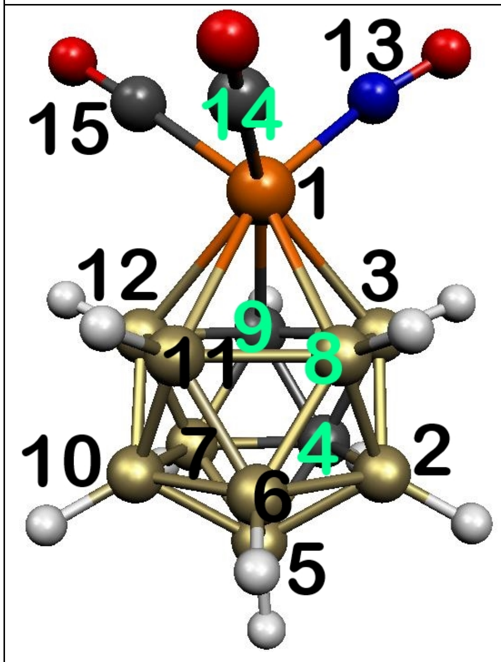

8. -741.66878590 +19.2 C_1

		1	2	3	4	5
1	Re	0.000000				
2	B	3.573074	0.000000			
3	B	2.297176	1.758124	0.000000		
4	C	3.449953	1.669940	1.688957	0.000000	
5	B	4.180505	1.759154	2.859773	1.682845	0.000000
6	B	3.588859	1.763055	2.855660	2.708157	1.754665
7	B	3.585347	2.845845	2.861841	1.683099	1.765676
8	B	2.337154	1.766719	1.785885	2.738981	2.869848
9	C	2.297141	2.750770	1.730701	1.612409	2.746115
10	B	3.604125	2.856342	3.369221	2.712391	1.756422
11	B	2.347547	2.867385	2.885619	3.205116	2.851920
12	B	2.327665	3.350943	2.867650	2.716306	2.849782
13	N	1.840140	4.480790	2.846190	4.326106	5.478728
14	C	1.976650	4.694986	3.835961	5.105449	5.438369
15	C	1.995652	5.331668	4.046144	4.795419	5.480831

		6	7	8	9	10

6	B	0.000000				
7	B	2.851510	0.000000			
8	B	1.770325	3.391027	0.000000		
9	C	3.237014	1.683864	2.808324	0.000000	
10	B	1.773989	1.756292	2.896362	2.747162	0.000000
11	B	1.763792	2.860015	1.814193	2.781118	1.768052
12	B	2.853966	1.759348	2.900968	1.703470	1.762813
13	N	5.035609	4.912911	3.454433	3.299638	5.286591
14	C	4.251870	5.190828	2.983750	4.186079	4.606150
15	C	5.099156	4.343830	4.219365	3.259980	4.485086

		11	12	13	14	15
11	B	0.000000				
12	B	1.774383	0.000000			
13	N	4.101103	3.988450	0.000000		
14	C	2.888039	3.715053	2.807389	0.000000	
15	C	3.522293	2.816476	2.837694	2.843582	0.000000

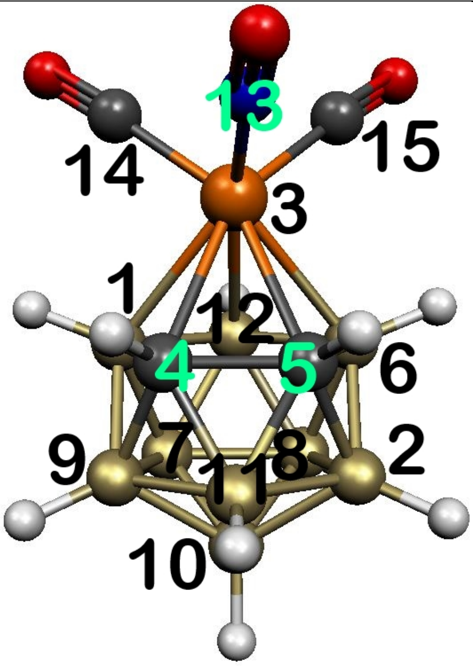

9. -741.66563440 +21.2 C_S

		1	2	3	4	5
1	B	0.000000				
2	B	3.352694	0.000000			
3	Re	2.326781	3.606932	0.000000		
4	C	1.690905	2.723567	2.316661	0.000000	
5	C	2.737086	1.673642	2.316693	1.622596	0.000000
6	B	2.860282	1.763539	2.328311	2.738313	1.687807
7	B	1.766571	2.849579	3.603976	2.725126	3.204778
8	B	2.853924	1.762569	3.600618	3.203859	2.718940
9	B	1.763486	2.842260	3.606037	1.675909	2.723159
10	B	2.861803	1.764413	4.215778	2.729920	2.727960
11	B	2.856708	1.754125	3.619802	1.691311	1.691076
12	B	1.786229	2.881869	2.340751	2.768185	2.767561

13	N	3.790887	4.641874	1.841247	3.100385	3.023398
14	C	2.840202	5.355238	1.976237	3.545208	4.164341
15	C	3.957537	4.563407	1.973930	4.189484	3.646942
		6	7	8	9	10
6	B	0.000000				
7	B	2.856718	0.000000			
8	B	1.763008	1.761500	0.000000		
9	B	3.352421	1.763226	2.847591	0.000000	
10	B	2.861080	1.764984	1.764811	1.765026	0.000000
11	B	2.855867	2.848268	2.846086	1.755601	1.760029
12	B	1.792043	1.773062	1.773595	2.877882	2.882516
13	N	3.678081	5.218187	5.164261	4.731521	5.488834
14	C	4.061374	4.375353	4.929560	4.505989	5.516679
15	C	2.860813	4.816635	4.308396	5.315577	5.493329
		11	12	13	14	15
11	B	0.000000				
12	B	3.389805	0.000000			
13	N	4.360252	4.148908	0.000000		
14	C	5.127587	3.250858	2.821336	0.000000	
15	C	5.184958	3.107957	2.824923	2.793299	0.000000

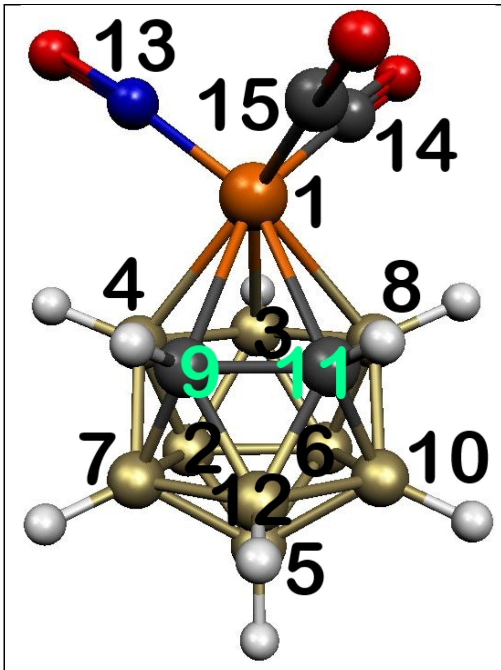

10. -741.66438110 +22.0 (C_S)

		1	2	3	4	5
1	Re	0.000000				
2	B	3.601524	0.000000			
3	B	2.346941	1.775341	0.000000		
4	B	2.316527	1.761671	1.785029	0.000000	
5	B	4.219518	1.763935	2.884338	2.861688	0.000000
6	B	3.602811	1.763498	1.767578	2.847240	1.768186
7	B	3.601509	1.757083	2.879785	1.765052	1.764778
8	B	2.341814	2.859041	1.800726	2.856932	2.857004
9	C	2.313254	2.729203	2.778137	1.703026	2.732703
10	B	3.621541	2.851941	2.888074	3.355074	1.764575
11	C	2.329385	3.200581	2.771811	2.735790	2.723945
12	B	3.628542	2.845602	3.396991	2.860475	1.758384
13	N	1.827184	4.512521	3.442813	2.893105	5.527058
14	C	1.972451	4.725391	3.004338	3.867268	5.461553
15	C	1.991812	5.363192	4.236904	4.051406	5.536327

		6	7	8	9	10
6	B	0.000000				
7	B	2.846367	0.000000			
8	B	1.761104	3.347060	0.000000		
9	C	3.204011	1.683128	2.729998	0.000000	
10	B	1.765808	2.843840	1.759188	2.723452	0.000000
11	C	2.714630	2.719452	1.678284	1.613825	1.673643
12	B	2.848629	1.754795	2.851492	1.691220	1.757494
13	N	5.034888	4.462233	4.075025	3.362967	5.303792
14	C	4.253228	5.266907	2.876882	4.183755	4.592512
15	C	5.136293	4.930830	3.554184	3.290269	4.540030

		11	12	13	14	15
11	C	0.000000				

	12 B 1.692409 0.000000<br><br>13 N 3.996095 4.982998 0.000000<br><br>14 C 3.701628 5.207482 2.773300 0.000000<br><br>15 C 2.960890 4.408532 2.805308 2.829755 0.000000

Table 5C. Energy ranking for $(CO)_2(NO)ReC_2B_9H_{11}$ obtained after B3LYP/6-31G(d)//SDD optimization:

No	Initial structure	Final energy (a.u.)	ΔE (kcal/mol)
1	03-Anticuboh-Re2-c----259_i-11	-741.4554829	0.00
2	03-Anticuboh-Re3-c----302	-741.4554667	0.01
3	03-Anticuboh-Re3-e----327	-741.4554654	0.01
4	03-Anticuboh-Re3-c----307	-741.4554642	0.01
5	03-Anticuboh-Re3-c----303	-741.4554551	0.02
6	03-Anticuboh-Re3-g----346	-741.4554551	0.02
7	03-Anticuboh-Re2-c----259_r-11	-741.4554386	0.03
8	02-Cuboctaedru-Re2-j----170	-741.4554043	0.05
9	02-Cuboctaedru-Re2-j----168	-741.4553572	0.08
10	02-Cuboctaedru-Re2-f----128	-741.4553485	0.08
11	02-Cuboctaedru-Re1-a----39	-741.4553356	0.09
12	03-Anticuboh-Re2-c----257	-741.4553018	0.11
13	03-Anticuboh-Re3-a----287	-741.4552981	0.12
14	03-Anticuboh-Re3-c----309	-741.4552946	0.12
15	02-Cuboctaedru-Re2-c----96_i-15	-741.4552899	0.12
16	02-Cuboctaedru-Re2-i----155_i-16	-741.4552897	0.12
17	02-Cuboctaedru-Re2-c----96_r-15	-741.4552717	0.13
18	02-Cuboctaedru-Re2-e----112_i-11	-741.4552701	0.13
19	02-Cuboctaedru-Re2-i----155_r-16	-741.4552697	0.13
20	02-Cuboctaedru-Re2-h----142_i-11	-741.4552689	0.13
21	03-Anticuboh-Re2-a----236	-741.4552687	0.13
22	03-Anticuboh-Re3-e----328	-741.4552565	0.14
23	02-Cuboctaedru-Re2-b----87	-741.4552562	0.14
24	02-Cuboctaedru-Re2-j----164	-741.4552553	0.14
25	02-Cuboctaedru-Re2-e----112_r-11	-741.4552334	0.16
26	02-Cuboctaedru-Re2-h----142_r-11	-741.4552325	0.16
27	02-Cuboctaedru-Re1-c----52	-741.4552258	0.16

28	03-Anticuboh-Re3-b----296	-741.4550317	0.28
29	03-Anticuboh-Re3-c----305	-741.4550317	0.28
30	03-Anticuboh-Re3-b----298	-741.4549947	0.31
31	03-Anticuboh-Re3-c----304	-741.4549826	0.31
32	02-Cuboctaedru-Re1-c----57	-741.4549493	0.33
33	02-Cuboctaedru-Re2-f----123	-741.4549234	0.35
34	02-Cuboctaedru-Re2-d----107	-741.4548989	0.37
35	02-Cuboctaedru-Re2-i----160	-741.4548964	0.37
36	02-Cuboctaedru-Re2-h----148_i-31	-741.4546571	0.52
37	02-Cuboctaedru-Re2-h----148_r-31	-741.4546173	0.54
38	02-Cuboctaedru-Re2-f----126	-741.4544943	0.62
39	02-Cuboctaedru-Re2-a----78	-741.4544942	0.62
40	03-Anticuboh-Re3-f----332	-741.4544821	0.63
41	03-Anticuboh-Re3-d----315	-741.4544441	0.65
42	03-Anticuboh-Re3-b----300	-741.4544434	0.65
43	03-Anticuboh-Re2-d----270	-741.4544073	0.67
44	03-Anticuboh-Re3-b----291_r-22	-741.4544024	0.68
45	02-Cuboctaedru-Re2-e----120	-741.4525451	1.84
46	02-Cuboctaedru-Re2-d----102	-741.4525444	1.84
47	02-Cuboctaedru-Re1-a----37	-741.4525303	1.85
48	01-Icos-Re-a----9	-741.4525165	1.86
49	01-Icos-Re-b----15	-741.4525132	1.86
50	03-Anticuboh-Re3-a----289	-741.4525083	1.87
51	02-Cuboctaedru-Re2-c----98	-741.4525036	1.87
52	02-Cuboctaedru-Re2-f----125	-741.4524949	1.88
53	02-Cuboctaedru-Re2-d----106	-741.4524835	1.88
54	03-Anticuboh-Re3-b----292	-741.4524818	1.88
55	02-Cuboctaedru-Re2-a----80	-741.4524806	1.88
56	03-Anticuboh-Re3-b----291_i-22	-741.4522230	2.05
57	02-Cuboctaedru-Re2-j----165	-741.4519723	2.20
58	02-Cuboctaedru-Re2-c----97	-741.4519683	2.21

59	01-Icos-Re-a----4	-741.4519503	2.22
60	03-Anticuboh-Re3-c----301	-741.4519359	2.23
61	02-Cuboctaedru-Re2-j----162	-741.4519220	2.23
62	02-Cuboctaedru-Re2-e----117	-741.4519208	2.24
63	02-Cuboctaedru-Re1-a----40	-741.4518986	2.25
64	02-Cuboctaedru-Re2-i----154	-741.4518821	2.26
65	02-Cuboctaedru-Re2-b----86	-741.4518763	2.26
66	02-Cuboctaedru-Re1-b----49	-741.4518728	2.27
67	03-Anticuboh-Re3-e----321	-741.4518604	2.27
68	03-Anticuboh-Re3-e----324	-741.4518421	2.28
69	03-Anticuboh-Re2-c----251	-741.4518415	2.29
70	02-Cuboctaedru-Re2-b----83	-741.4518333	2.29
71	02-Cuboctaedru-Re2-a----76	-741.4518310	2.29
72	02-Cuboctaedru-Re2-i----156	-741.4518302	2.29
73	03-Anticuboh-Re3-f----338	-741.4518270	2.29
74	03-Anticuboh-Re3-c----310	-741.4518099	2.30
75	03-Anticuboh-Re3-d----317	-741.4518099	2.30
76	03-Anticuboh-Re3-e----325	-741.4518077	2.31
77	03-Anticuboh-Re3-b----297	-741.4518015	2.31
78	03-Anticuboh-Re3-d----312	-741.4517837	2.32
79	03-Anticuboh-Re2-c----260	-741.4517804	2.32
80	02-Cuboctaedru-Re1-b----46	-741.4517723	2.33
81	02-Cuboctaedru-Re1-c----55	-741.4517537	2.34
82	02-Cuboctaedru-Re2-c----99	-741.4500586	3.40
83	02-Cuboctaedru-Re2-k----175	-741.4500580	3.40
84	02-Cuboctaedru-Re2-k----172	-741.4500552	3.41
85	02-Cuboctaedru-Re2-e----119	-741.4500550	3.41
86	03-Anticuboh-Re3-a----284	-741.4500529	3.41
87	03-Anticuboh-Re3-d----313	-741.4500212	3.43
88	01-Icos-Re-a----8	-741.4500201	3.43
89	01-Icos-Re-c----29	-741.4500201	3.43

90	03-Anticuboh-Re3-g----350	-741.4500193	3.43
91	02-Cuboctaedru-Re2-a----79	-741.4500136	3.43
92	02-Cuboctaedru-Re1-d----68	-741.4499838	3.45
93	03-Anticuboh-Re3-a----283	-741.4499816	3.45
94	03-Anticuboh-Re3-g----349	-741.4499816	3.45
95	02-Cuboctaedru-Re2-b----89	-741.4499750	3.46
96	02-Cuboctaedru-Re1-d----63	-741.4499453	3.47
97	02-Cuboctaedru-Re2-k----176	-741.4492506	3.91
98	03-Anticuboh-Re2-e----280	-741.4492189	3.93
99	02-Cuboctaedru-Re1-a----38_i-16	-741.4492045	3.94
100	02-Cuboctaedru-Re1-d----64_i-16	-741.4492035	3.94
101	02-Cuboctaedru-Re1-a----38_r-16	-741.4491835	3.95
102	02-Cuboctaedru-Re1-d----64_r-16	-741.4491835	3.95
103	03-Anticuboh-Re3-e----323	-741.4491634	3.97
104	03-Anticuboh-Re3-g----347	-741.4491634	3.97
105	02-Cuboctaedru-Re1-a----35	-741.4489296	4.11
106	02-Cuboctaedru-Re2-e----115	-741.4489289	4.11
107	03-Anticuboh-Re3-a----281	-741.4489251	4.12
108	02-Cuboctaedru-Re2-a----72	-741.4489247	4.12
109	03-Anticuboh-Re1-a----189	-741.4489076	4.13
110	03-Anticuboh-Re3-d----318	-741.4489000	4.13
111	03-Anticuboh-Re3-e----330	-741.4489000	4.13
112	02-Cuboctaedru-Re1-b----43	-741.4488717	4.15
113	01-Icos-Re-a----5	-741.4488495	4.16
114	02-Cuboctaedru-Re1-b----41	-741.4483345	4.49
115	02-Cuboctaedru-Re2-e----114	-741.4483114	4.50
116	02-Cuboctaedru-Re2-c----92	-741.4483095	4.50
117	03-Anticuboh-Re3-a----286	-741.4482945	4.51
118	03-Anticuboh-Re3-f----339	-741.4482924	4.51
119	01-Icos-Re-a----6	-741.4482679	4.53
120	02-Cuboctaedru-Re1-a----33	-741.4482560	4.54

121	02-Cuboctaedru-Re1-c----56_r-23	-741.4301205	15.92
122	02-Cuboctaedru-Re2-f----130	-741.4300749	15.94
123	02-Cuboctaedru-Re2-d----109	-741.4300688	15.95
124	03-Anticuboh-Re3-f----336	-741.4294066	16.36
125	03-Anticuboh-Re3-c----306	-741.4294041	16.36
126	03-Anticuboh-Re3-b----294	-741.4293865	16.38
127	03-Anticuboh-Re3-f----333	-741.4293772	16.38
128	02-Cuboctaedru-Re2-j----163	-741.4293664	16.39
129	02-Cuboctaedru-Re2-h----147	-741.4293641	16.39
130	03-Anticuboh-Re3-f----335	-741.4293319	16.41
131	03-Anticuboh-Re3-b----295	-741.4293313	16.41
132	02-Cuboctaedru-Re2-h----150	-741.4292235	16.48
133	02-Cuboctaedru-Re2-i----157	-741.4292092	16.49
134	02-Cuboctaedru-Re2-f----127	-741.4292046	16.49
135	02-Cuboctaedru-Re2-i----158	-741.4292045	16.49
136	02-Cuboctaedru-Re2-d----103_r-54	-741.4291329	16.54
137	03-Anticuboh-Re3-g----343	-741.4290666	16.58
138	02-Cuboctaedru-Re1-d----67	-741.4290573	16.58
139	03-Anticuboh-Re2-b----244	-741.4290127	16.61
140	03-Anticuboh-Re2-e----274	-741.4290117	16.61
141	03-Anticuboh-Re2-e----275	-741.4289975	16.62
142	02-Cuboctaedru-Re1-c----58	-741.4289830	16.63
143	02-Cuboctaedru-Re1-d----62	-741.4289827	16.63
144	03-Anticuboh-Re3-b----293	-741.4289823	16.63
145	03-Anticuboh-Re3-g----344	-741.4289823	16.63
146	03-Anticuboh-Re2-c----255	-741.4289747	16.63
147	03-Anticuboh-Re2-e----276	-741.4289438	16.65
148	02-Cuboctaedru-Re2-f----129	-741.4289261	16.66
149	02-Cuboctaedru-Re2-k----179	-741.4289238	16.67
150	02-Cuboctaedru-Re2-i----159	-741.4289225	16.67
151	02-Cuboctaedru-Re2-k----177	-741.4289217	16.67

152	02-Cuboctaedru-Re2-d----110	-741.4289116	16.67
153	02-Cuboctaedru-Re2-k----180	-741.4289108	16.67
154	02-Cuboctaedru-Re1-c----56_i-23	-741.4288921	16.69
155	02-Cuboctaedru-Re2-h----149	-741.4288451	16.72
156	02-Cuboctaedru-Re2-d----103_i-54	-741.4288446	16.72
157	02-Cuboctaedru-Re2-k----173	-741.4288428	16.72
158	03-Anticuboh-Re3-g----345	-741.4288330	16.72
159	02-Cuboctaedru-Re2-j----167	-741.4286901	16.81
160	03-Anticuboh-Re3-f----334	-741.4285298	16.91
161	02-Cuboctaedru-Re1-d----70	-741.4284978	16.93
162	03-Anticuboh-Re3-g----348	-741.4284748	16.95
163	02-Cuboctaedru-Re2-k----178	-741.4284595	16.96
164	02-Cuboctaedru-Re1-d----69	-741.4284257	16.98
165	03-Anticuboh-Re2-a----234	-741.4261271	18.42
166	03-Anticuboh-Re2-b----248	-741.4261271	18.42
167	02-Cuboctaedru-Re2-c----100	-741.4260989	18.44
168	02-Cuboctaedru-Re2-d----105	-741.4260984	18.44
169	01-Icos-Re-b----19	-741.4260702	18.46
170	02-Cuboctaedru-Re2-f----122	-741.4260695	18.46
171	01-Icos-Re-a----2	-741.4260687	18.46
172	02-Cuboctaedru-Re2-e----118	-741.4260684	18.46
173	03-Anticuboh-Re2-b----243	-741.4260599	18.46
174	02-Cuboctaedru-Re2-d----104	-741.4260408	18.48
175	02-Cuboctaedru-Re2-b----90	-741.4260406	18.48
176	02-Cuboctaedru-Re1-b----50	-741.4258849	18.57
177	02-Cuboctaedru-Re1-c----54	-741.4258750	18.58
178	02-Cuboctaedru-Re1-b----42	-741.4258723	18.58
179	02-Cuboctaedru-Re2-i----152_i-14	-741.4255342	18.79
180	02-Cuboctaedru-Re2-e----116_i-14	-741.4255326	18.79
181	03-Anticuboh-Re2-d----262	-741.4255206	18.80
182	02-Cuboctaedru-Re1-c----53	-741.4255062	18.81

183	02-Cuboctaedru-Re1-a----32	-741.4255028	18.81
184	03-Anticuboh-Re3-a----288	-741.4254935	18.82
185	03-Anticuboh-Re3-e----329	-741.4254935	18.82
186	03-Anticuboh-Re3-a----285	-741.4254842	18.82
187	03-Anticuboh-Re3-b----299	-741.4254842	18.82
188	03-Anticuboh-Re3-c----308	-741.4254751	18.83
189	02-Cuboctaedru-Re2-e----116_r-14	-741.4254575	18.84
190	02-Cuboctaedru-Re2-i----152_r-14	-741.4254550	18.84
191	03-Anticuboh-Re2-c----258	-741.4254546	18.84
192	03-Anticuboh-Re2-a----237	-741.4254545	18.84
193	03-Anticuboh-Re3-a----282	-741.4254183	18.87
194	02-Cuboctaedru-Re2-c----94	-741.4243937	19.51
195	02-Cuboctaedru-Re2-b----84	-741.4243810	19.52
196	02-Cuboctaedru-Re2-b----85_i-14	-741.4243785	19.52
197	02-Cuboctaedru-Re2-a----74	-741.4243531	19.53
198	03-Anticuboh-Re2-a----233	-741.4243507	19.54
199	03-Anticuboh-Re2-b----247	-741.4243476	19.54
200	02-Cuboctaedru-Re2-b----85_r-14	-741.4243250	19.55
201	02-Cuboctaedru-Re1-a----31	-741.4243106	19.56
202	02-Cuboctaedru-Re2-b----82	-741.4242836	19.58
203	02-Cuboctaedru-Re2-e----113	-741.4242831	19.58
204	02-Cuboctaedru-Re1-a----34	-741.4236802	19.96
205	02-Cuboctaedru-Re1-b----44	-741.4236802	19.96
206	03-Anticuboh-Re1-b----194	-741.4236073	20.00
207	03-Anticuboh-Re3-f----340	-741.4219124	21.07
208	03-Anticuboh-Re3-d----316	-741.4219118	21.07
209	03-Anticuboh-Re2-a----240	-741.4144438	25.75
210	03-Anticuboh-Re3-a----290	-741.4144348	25.76
211	03-Anticuboh-Re3-d----320	-741.4144328	25.76
212	03-Anticuboh-Re2-b----249	-741.4085586	29.45
213	01-Icos-Re-a----10	-741.4009226	34.24

214	03-Anticuboh-Re1-b----198	-741.4009182	34.24
215	03-Anticuboh-Re1-a----185	-741.4006447	34.41
216	03-Anticuboh-Re1-e----223	-741.3999037	34.88
217	02-Cuboctaedru-Re2-d----101	-741.3994517	35.16
218	02-Cuboctaedru-Re2-g----131	-741.3994517	35.16
219	01-Icos-Re-b----14	-741.3994362	35.17
220	03-Anticuboh-Re2-b----245	-741.3989969	35.45
221	02-Cuboctaedru-Re2-h----146	-741.3989049	35.50
222	02-Cuboctaedru-Re2-i----153	-741.3989049	35.50
223	01-Icos-Re-b----13	-741.3986446	35.67
224	03-Anticuboh-Re2-c----254	-741.3977438	36.23
225	03-Anticuboh-Re2-b----246	-741.3977414	36.23
226	01-Icos-Re-b----20	-741.3975185	36.37
227	03-Anticuboh-Re2-c----253	-741.3975144	36.38
228	03-Anticuboh-Re1-b----193	-741.3966775	36.90
229	03-Anticuboh-Re2-d----265	-741.3965377	36.99
230	03-Anticuboh-Re1-e----225	-741.3963549	37.10
231	03-Anticuboh-Re1-a----184	-741.3963522	37.11
232	03-Anticuboh-Re1-d----215	-741.3963302	37.12
233	03-Anticuboh-Re2-b----241	-741.3963232	37.12
234	03-Anticuboh-Re1-e----226	-741.3956813	37.53
235	03-Anticuboh-Re1-a----190	-741.3956694	37.53
236	03-Anticuboh-Re1-e----222	-741.3956671	37.54
237	03-Anticuboh-Re2-e----273	-741.3955774	37.59
238	01-Icos-Re-c----27	-741.3955723	37.60
239	03-Anticuboh-Re1-c----203	-741.3955346	37.62
240	03-Anticuboh-Re1-c----207	-741.3955165	37.63
241	03-Anticuboh-Re1-c----209	-741.3947292	38.12
242	03-Anticuboh-Re1-e----224	-741.3945614	38.23
243	02-Cuboctaedru-Re2-g----133	-741.3945577	38.23
244	02-Cuboctaedru-Re2-h----141	-741.3945577	38.23

245	03-Anticuboh-Re1-a----183	-741.3945481	38.24
246	03-Anticuboh-Re2-d----261	-741.3945365	38.25
247	02-Cuboctaedru-Re1-d----61	-741.3943373	38.37
248	03-Anticuboh-Re1-c----204	-741.3939233	38.63
249	03-Anticuboh-Re1-a----186	-741.3938988	38.65
250	03-Anticuboh-Re1-c----202	-741.3938988	38.65
251	01-Icos-Re-c----26	-741.3938739	38.66
252	03-Anticuboh-Re1-c----205	-741.3938075	38.70
253	03-Anticuboh-Re1-c----210	-741.3932649	39.04
254	03-Anticuboh-Re1-e----227	-741.3932649	39.04
255	01-Icos-Re-c----21	-741.3932464	39.05
256	01-Icos-Re-c----25	-741.3932255	39.07
257	02-Cuboctaedru-Re2-k----171	-741.3932235	39.07
258	02-Cuboctaedru-Re2-g----140	-741.3932200	39.07
259	03-Anticuboh-Re1-d----214	-741.3923796	39.60
260	03-Anticuboh-Re1-d----213	-741.3922652	39.67
261	03-Anticuboh-Re1-d----218	-741.3922613	39.67
262	02-Cuboctaedru-Re2-f----121	-741.3892495	41.56
263	02-Cuboctaedru-Re2-g----139	-741.3892495	41.56
264	01-Icos-Re-b----11	-741.3892482	41.56
265	03-Anticuboh-Re1-a----181	-741.3887947	41.85
266	03-Anticuboh-Re1-d----212	-741.3887947	41.85
267	02-Cuboctaedru-Re2-c----91	-741.3887874	41.85
268	02-Cuboctaedru-Re2-g----135	-741.3887874	41.85
269	02-Cuboctaedru-Re1-d----66	-741.3877765	42.49
270	03-Anticuboh-Re3-d----314	-741.3877029	42.53
271	03-Anticuboh-Re3-d----319	-741.3872601	42.81
272	03-Anticuboh-Re2-d----268	-741.3871530	42.88
273	03-Anticuboh-Re2-a----232	-741.3871476	42.88
274	03-Anticuboh-Re1-d----217	-741.3871209	42.90
275	03-Anticuboh-Re2-d----267	-741.3871193	42.90

276	03-Anticuboh-Re1-c----201	-741.3871186	42.90
277	02-Cuboctaedru-Re1-a----36	-741.3870975	42.91
278	02-Cuboctaedru-Re2-a----77	-741.3864328	43.33
279	02-Cuboctaedru-Re2-j----166	-741.3864327	43.33
280	03-Anticuboh-Re2-a----239	-741.3864156	43.34
281	03-Anticuboh-Re3-f----331	-741.3863996	43.35
282	03-Anticuboh-Re2-a----235	-741.3863563	43.38
283	03-Anticuboh-Re2-e----278	-741.3863563	43.38
284	03-Anticuboh-Re2-e----277	-741.3863262	43.40
285	03-Anticuboh-Re3-g----341	-741.3863178	43.40
286	02-Cuboctaedru-Re2-b----81	-741.3856764	43.80
287	02-Cuboctaedru-Re2-g----134	-741.3856738	43.81
288	03-Anticuboh-Re2-b----250	-741.3856540	43.82
289	03-Anticuboh-Re2-a----238	-741.3846373	44.46
290	03-Anticuboh-Re1-b----197	-741.3840193	44.84
291	02-Cuboctaedru-Re1-c----51	-741.3819720	46.13
292	02-Cuboctaedru-Re2-h----143	-741.3819601	46.14
293	02-Cuboctaedru-Re2-b----88	-741.3819061	46.17
294	02-Cuboctaedru-Re2-f----124	-741.3819061	46.17
295	02-Cuboctaedru-Re2-k----174	-741.3811138	46.67
296	01-Icos-Re-b----12	-741.3735840	51.39
297	03-Anticuboh-Re1-c----206	-741.3729202	51.81
298	01-Icos-Re-c----24	-741.3729196	51.81
299	01-Icos-Re-c----23	-741.3728923	51.83
300	01-Icos-Re-b----18	-741.3727258	51.93
301	01-Icos-Re-a----1	-741.3722640	52.22
302	01-Icos-Re-c----28	-741.3717126	52.57
303	01-Icos-Re-c----30	-741.3715883	52.65
304	03-Anticuboh-Re2-e----271	-741.3715042	52.70
305	01-Icos-Re-b----17	-741.3713608	52.79
306	01-Icos-Re-c----22	-741.3713608	52.79

307	01-Icos-Re-a----7	-741.3713320	52.81
308	01-Icos-Re-a----3	-741.3711104	52.95
309	01-Icos-Re-b----16	-741.3710884	52.96
310	03-Anticuboh-Re2-b----242	-741.3709156	53.07
311	03-Anticuboh-Re2-d----263	-741.3709156	53.07
312	03-Anticuboh-Re2-d----269	-741.3697710	53.79
313	02-Cuboctaedru-Re2-a----75	-741.3695059	53.95
314	02-Cuboctaedru-Re2-c----95	-741.3695059	53.95
315	02-Cuboctaedru-Re2-c----93_i-12	-741.3692238	54.13
316	02-Cuboctaedru-Re2-h----144_i-12	-741.3692238	54.13
317	02-Cuboctaedru-Re2-c----93_r-12	-741.3691866	54.15
318	02-Cuboctaedru-Re2-h----144_r-12	-741.3691866	54.15
319	02-Cuboctaedru-Re1-d----65	-741.3691292	54.19
320	02-Cuboctaedru-Re1-b----48	-741.3691279	54.19
321	03-Anticuboh-Re1-b----199	-741.3690713	54.23
322	02-Cuboctaedru-Re1-c----59	-741.3681986	54.77
323	03-Anticuboh-Re2-c----256	-741.3681903	54.78
324	03-Anticuboh-Re1-b----191	-741.3679023	54.96
325	03-Anticuboh-Re1-d----219	-741.3679023	54.96
326	02-Cuboctaedru-Re2-a----73	-741.3678916	54.97
327	02-Cuboctaedru-Re2-h----145	-741.3678916	54.97
328	03-Anticuboh-Re3-e----326	-741.3657342	56.32
329	03-Anticuboh-Re3-f----337	-741.3657259	56.32
330	03-Anticuboh-Re1-d----216	-741.3654017	56.53
331	02-Cuboctaedru-Re2-g----137	-741.3653635	56.55
332	02-Cuboctaedru-Re2-i----151	-741.3653635	56.55
333	02-Cuboctaedru-Re2-g----138	-741.3647376	56.94
334	02-Cuboctaedru-Re2-j----161	-741.3647376	56.94
335	03-Anticuboh-Re1-b----196	-741.3640724	57.36
336	03-Anticuboh-Re1-d----211	-741.3623125	58.47
337	03-Anticuboh-Re1-e----221	-741.3623125	58.47

338	02-Cuboctaedru-Re2-a----71	-741.3622751	58.49
339	02-Cuboctaedru-Re2-g----136	-741.3622751	58.49
340	03-Anticuboh-Re1-d----220	-741.3622660	58.50
341	02-Cuboctaedru-Re1-b----47	-741.3597058	60.10
342	03-Anticuboh-Re3-d----311	-741.3554948	62.74
343	02-Cuboctaedru-Re2-e----111	-741.3551186	62.98
344	02-Cuboctaedru-Re2-g----132	-741.3551186	62.98
345	03-Anticuboh-Re2-c----252	-741.3486355	67.05
346	03-Anticuboh-Re2-d----266	-741.3486355	67.05
347	03-Anticuboh-Re2-e----279	-741.3406244	72.08
348	02-Cuboctaedru-Re2-j----169	-741.3405988	72.09
349	03-Anticuboh-Re1-e----230	-741.3400350	72.45
350	03-Anticuboh-Re1-a----182	-741.3363931	74.73
351	03-Anticuboh-Re1-b----200	-741.3362751	74.81
352	03-Anticuboh-Re1-e----229	-741.3362751	74.81
353	03-Anticuboh-Re3-e----322	-741.3332387	76.71
354	03-Anticuboh-Re3-g----342	-741.3290708	79.33
355	03-Anticuboh-Re1-b----192	-741.3283004	79.81
356	03-Anticuboh-Re1-a----188	-741.3282948	79.81
357	02-Cuboctaedru-Re1-c----60	-741.3257130	81.43
358	03-Anticuboh-Re1-e----228	-741.3237747	82.65
359	02-Cuboctaedru-Re1-b----45	-741.3115816	90.30
360	02-Cuboctaedru-Re2-d----108	-741.2952580	100.54
361	03-Anticuboh-Re2-a----231_i-51	-741.2952028	100.58
362	03-Anticuboh-Re2-a----231_r-51	-741.2951297	100.62
363	03-Anticuboh-Re1-c----208	-741.2948456	100.80
364	03-Anticuboh-Re1-b----195	-741.2933919	101.72
365	03-Anticuboh-Re2-d----264	-741.2884455	104.82
366	03-Anticuboh-Re2-e----272	-741.2884455	104.82
367	03-Anticuboh-Re1-a----187	-741.2737998	114.01

Table 6S. Orbital energies and HOMO-LUMO gaps of the lowest lying structures.

Structure	HOMO energy (Hartree)	LUMO energy (Hartree)	Gap (Hartree)	Gap (eV)
01-08v-ReC2H2BnHn	-0.236287	-0.128431	0.107856	2.93
02-08v-ReC2H2BnHn	-0.237304	-0.128033	0.109271	2.97
03-08v-ReC2H2BnHn	-0.235699	-0.136986	0.098713	2.69
04-08v-ReC2H2BnHn	-0.232647	-0.137983	0.094664	2.58
05-08v-ReC2H2BnHn	-0.222361	-0.130688	0.091673	2.49
06-08v-ReC2H2BnHn	-0.228813	-0.137349	0.091464	2.49
01-09v-ReC2H2BnHn	-0.227717	-0.135226	0.092491	2.52
02-09v-ReC2H2BnHn	-0.238346	-0.133710	0.104636	2.85
03-09v-ReC2H2BnHn	-0.224069	-0.131786	0.092283	2.51
04-09v-ReC2H2BnHn	-0.228472	-0.139580	0.088892	2.42
05-09v-ReC2H2BnHn	-0.221451	-0.139136	0.082315	2.24
06-09v-ReC2H2BnHn	-0.216942	-0.133682	0.083260	2.27
07-09v-ReC2H2BnHn	-0.222651	-0.138458	0.084193	2.29
08-09v-ReC2H2BnHn	-0.221953	-0.136392	0.085561	2.33
09-09v-ReC2H2BnHn	-0.236834	-0.138228	0.098606	2.68
01-10v-ReC2H2BnHn	-0.241560	-0.132646	0.108914	2.96
02-10v-ReC2H2BnHn	-0.247164	-0.136235	0.110929	3.02
03-10v-ReC2H2BnHn	-0.239196	-0.129222	0.109974	2.99
04-10v-ReC2H2BnHn	-0.240348	-0.134791	0.105557	2.87
05-10v-ReC2H2BnHn	-0.240311	-0.130533	0.109778	2.99
06-10v-ReC2H2BnHn	-0.231321	-0.126172	0.105149	2.86
07-10v-ReC2H2BnHn	-0.240301	-0.139946	0.100355	2.73
01-11v-ReC2H2BnHn	-0.238562	-0.150160	0.088402	2.41
02-11v-ReC2H2BnHn	-0.220297	-0.138191	0.082106	2.23
03-11v-ReC2H2BnHn	-0.237305	-0.132087	0.105218	2.86
04-11v-ReC2H2BnHn	-0.226523	-0.147881	0.078642	2.14
05-11v-ReC2H2BnHn	-0.230574	-0.135536	0.095038	2.59
06-11v-ReC2H2BnHn	-0.243503	-0.136742	0.106761	2.91
07-11v-ReC2H2BnHn	-0.234233	-0.134471	0.099762	2.71
08-11v-ReC2H2BnHn	-0.241938	-0.137429	0.104509	2.84
09-11v-ReC2H2BnHn	-0.235438	-0.138952	0.096486	2.63
10-11v-ReC2H2BnHn	-0.233097	-0.140840	0.092257	2.51
01-12v-ReC2H2BnHn	-0.238068	-0.128287	0.109781	2.99
02-12v-ReC2H2BnHn	-0.241725	-0.137008	0.104717	2.85
03-12v-ReC2H2BnHn	-0.246246	-0.138350	0.107896	2.94
04-12v-ReC2H2BnHn	-0.238702	-0.134978	0.103724	2.82
05-12v-ReC2H2BnHn	-0.252472	-0.148645	0.103827	2.83
06-12v-ReC2H2BnHn	-0.236556	-0.126736	0.109820	2.99
07-12v-ReC2H2BnHn	-0.234142	-0.124456	0.109686	2.98
08-12v-ReC2H2BnHn	-0.244257	-0.137933	0.106324	2.89

09-12v-ReC2H2BnHn	-0.251985	-0.151910	0.100075	2.72
10-12v-ReC2H2BnHn	-0.251138	-0.146337	0.104801	2.85
